# Advanced dense nitrides: from discovery to potential applications

**DOI:** 10.1080/14686996.2026.2718044

**Published:** 2026-08-20

**Authors:** Andreas Zerr

**Affiliations:** Laboratoire des Sciences des Procédés et des Matériaux, CNRS UPR 3407, Université Sorbonne Paris Nord, Villetaneuse, France

**Keywords:** Spinel nitrides, Th_3_P_4_-type nitrides, U_2_S_3_-type nitrides, high-pressure synthesis, crystal structure, mechanical properties, optoelectronic properties, energy materials, photoelectrochemical splitting of water, green hydrogen

## Abstract

This work reviews evolution of search for novel high-pressure high-temperature (HP-HT) nitrides with nitrogen-to-metal ratio well above one which has commenced more than 25 years ago and brought today some remarkable blooms. The number of reports on synthesis of such nitrides grows permanently but their majority deals, in the last years, with synthesis at very high pressures where even characterisation of the products (structure, composition etc.) becomes vague. The number of binary nitrides and related ternary compounds or solid solutions, accessible as macroscopic samples using large volume HP-HT apparatuses, appears to have stabilised at a high but still monitorable level. Today, the Gretchen question is whether the outcome of the 25-years activity was rewarding, whether any of the nitrides recoverable at ambient conditions exhibits properties deserving transfer to the industry. An answer is not straight because the efforts needed to obtain reliable property data go beyond those invested during the synthesis and characterisation. Gathering of such data requires either laborious investment in samples quality or development/adaptation of non-traditional, for HP-HT research, *in-* and *ex-situ* techniques. Here, the exposé of the most notable novel nitrides and their HP-HT synthesis routes is followed by examples of several recent developments/adaptations of experimental approaches permitting access to mechanical and optoelectronic properties which were earlier difficult to measure due to imperfect polycrystalline, porous and eventually contaminated samples. The revealed promising properties allow us to hope that the efforts were not futile and, at least, a couple of the discovered HP-HT nitrides could find industrial applications.

## IMPACT STATEMENT

Progress in synthesis, characterisation, measurement of mechanical and optoelectronic properties of the high-pressure nitrides of the group 4, 5, and 14 elements of the periodic table with N:*M* > 1 is reviewed.

Progress and challenges by high pressure – high temperature synthesis, characterisation, and measurement of properties of novel advanced nitrides, found to be hard, stiff but also promising energy materials is reviewed.

## GRAPHICAL ABSTRACT

**Figure:**
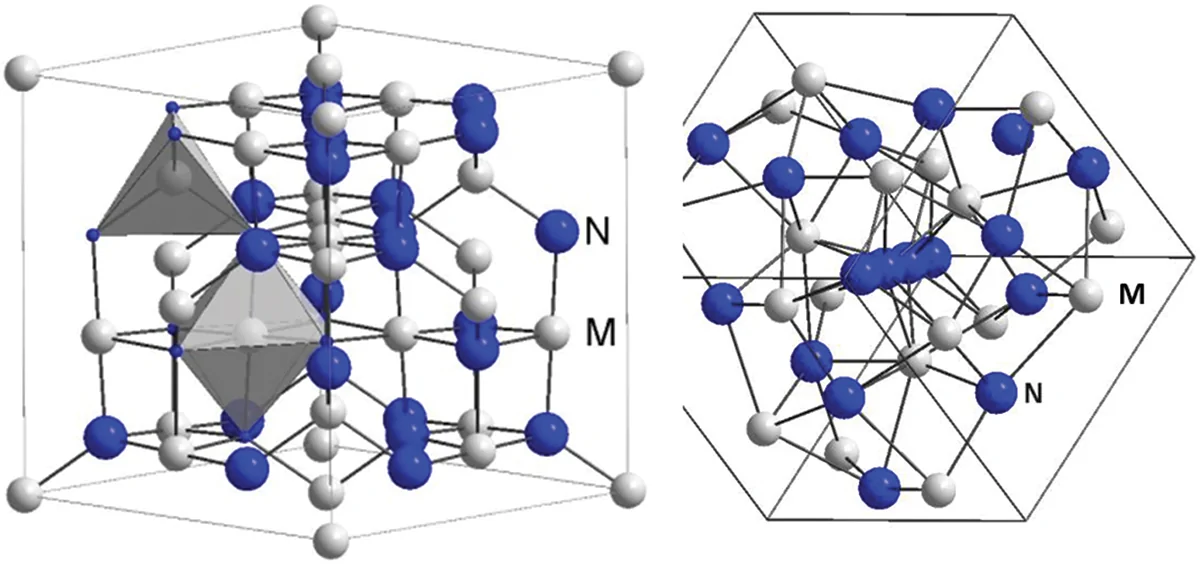
Progress in the HP-HT synthesis, characterisation, and properties’ measurement of novel advanced nitrides is reviewed.

## 1. Introduction

Physical properties of a solid compound are defined by its elemental composition (including contaminations and/or doping), crystal structure and texture/microstructure (e.g. single crystal, polycrystal, film, nano-powder etc.). While the latter characteristic is controlled by processing, the first two are the fundamental ones defining suitability of a compound for applications. A large portion of inorganic solids used by mankind are either minerals and rocks collected from or close to the Earth’s surface or products of chemical reactions performed at low pressures (below 1 GPa = 10 kbar = 10000 atm). Moreover, domination of oxygen in the Earth’s interior and in the atmosphere as well as its higher electronegativity^1^ at low pressures, when compared with nitrogen, explains why the vast majority of known and characterised inorganic solids contain only oxygen anions. According to the quantitative analysis of two databases, JCPDS and Inspec, the number of compounds containing only oxygen anions (O^2-^) is 10–20 higher than that of compounds containing only nitrogen anions (N^3-^). Moreover, most of the known nitrides are synthetic. Furthermore, the lower electronegativity of nitrogen at low pressures often results in sub-nitrides even though nitrogen is available in excess. Here, we define as subnitride a binary or ternary compound with the nitrogen:metal(s) ratio (N:M) below that when the metal is completely oxidised, e.g. all valence electrons of the metal are bonded to nitrogen anions [2]. In the latter case we talk about stoichiometric nitrides. For example, reaction of hafnium with nitrogen at low pressures and high temperatures results in the subnitride δ-HfN (or hafnium mononitride, N:*M* = 1) while the available number of valence electrons in a Hf-atom, namely four, permits formation of the stoichiometric nitride with the composition Hf_3_N_4_. In contrast, reaction of hafnium with oxygen in access leads to HfO_2_. Apparently, the above consideration does not concern (1) azides, MN_3_, forming upon reaction of a metal with hydrazoic acid, HN_3_, (2) products of reactions of metals with ammonia, NH_3_, and (3) compounds obtained via non-equilibrium processes such as deposition of films via, for example, plasma-enhanced reactive magnetron sputtering. For the reader information, Table 1 lists binary M-N compounds reported by end of the 20^th^ century even though their compositions were not always rigorously measured. The main source for this Table 1 was the book series ‘Gmelin Handbook of Inorganic and Organometallic Chemistry’ [3]. Methods of synthesis of nitrides at low pressures, which can also be applied at high pressures, are presented elsewhere [4]. Accordingly, one of the most promising ways to find new compounds exhibiting advanced properties or a previously-not-accessible property combination is synthesis of higher nitrides with the N:*M* > 1. (Here, the definition of higher nitrides is more general than that in an earlier review [5] where such compounds had to have formal oxidation states (OS) attaining those found among corresponding oxides.) Waist majority of ternary and quaternary nitrides is still waiting to be discovered but the recently found binary nitrides are reason for hope.

**Table 1.** Binary nitrides of elements of the groups 1 to 16 of the periodic table and of lanthanides reported by end of the 20th century. The main source is Gmelin Handbook of inorganic and organometallic chemistry [3] indicated by (G). In a few cases, the synthesis methods are indicated: (F) – deposition as a film, (ii) – ion implantation, (HP) – high-pressure synthesis.

**Group 1** Li: Li_3_N (G), LiN_3_ (G) Na: Na_3_N (G), NaN (G), NaN_3_ (G) K: K_3_N (G), KN_3_ (G) Rb: Rb_3_N (G), RbN_3_ (G) Cs: Cs_3_N (G), CsN_3_ (G) **Group 2** Be: Be_3_N_2_ (G) Mg: Mg_3_N_2_ (G), Mg(N_3_)_2_ (G) Ca: Ca_3_N_2_ (G), Ca_2_N_2_ (G), CaN (G), Ca_3_N_4_ (G), Ca(N_3_)_2_ (G) Sr: Sr_3_N_2_ (G), Sr_3_N_4_ (G), Sr(N_3_)_2_ (G) Ba: BaN_6_ (G), Ba_3_N_2_ (G) **Group 3** Sc: ScN (G) Y: YN (G) La: LaN (G) **Group 4** Ti: Ti_2_N (G), TiN (G), TiN_x_ (G), Ti_3_N_4_ (G) Zr: Zr_3_N_2_ (G), ZrN (G), Zr_3_N_4_ (G), Zr_2_N_3_ (G), Zr_3_N_8_ (G) Hf: HfN (G), Hf_3_N_4_ (F) **Group 5** V: V_8_N, V_4_N,V_3_N (G), V_5_N_2_ (G), V_2_N (G), VN (G), VN_x_ (G), VN_2_ (G) Nb: Nb_2_N (G), Nb_4_N_3_ (G), NbN (G), Nb_5_N_6_ (F), Nb_4_N_5_ (F), Nb_3_N_4_ (F), Nb_3_N_5_ (G) Ta: Ta_27_N (G), Ta_2_N (G), TaN (G), TaN_x_ (G), Ta_5_N_6_ (F), Ta_4_N_5_ (F), Ta_3_N_5_ (G) **Group 6** Cr: Cr_3_N (G), Cr_2_N (G), Cr_3_N_2_ (G), CrN (G), CrN_9_ (G) Mo: Mo_2_N (G), Mo_3_N_2_ (G), MoN (HP), Mo_2_N_3_ (F), MoN_2_ (G) W: W_2_N_3_ (G), WN_2_ (G) **Group 7** Mn: Mn_4_N (G), Mn_7_N_2_ (G), Mn_3_N (G), Mn_2_N (G), Mn_5_N_2_ (G), Mn_3_N_2_ (G), Mn_6_N_5_ (G), MnN (G), Mn(N_3_)_2_ (G) Tc: n.a. Re: Re_3_N, ReN (ii) **Group 8** Fe: Fe_12_N (G), Fe_8_N (G), Fe_6_N (G), Fe_4_N (G), Fe_7_N_2_ (G), Fe_3_N (G), Fe_5_N_2_ (G), Fe_2_N (G), Fe_3_N_2_ (G), FeN (G), FeN_9_ (G) Ru: RuN (G) Os: n.a. **Group 9** Co: CoN_0.28_ (G), Co_7_N_2_ (G), Co_3_N (G), Co_2_N (G), Co_3_N_2_ (G), CoN (G), CoN_6_ (G) Rh: RhN_2_ (G) Ir: n.a.	**Group 10** Ni: Ni_4_N (G), Ni_3_N (G), Ni_3_N_2_ (G), NiN_6_ (G) Pd: n.a. Pt: n.a. **Group 11** Cu: Cu_3_N (G), CuN_3_ (G), Cu(N_3_)_2_ (G) Ag: Ag_3_N (G), AgN (G), AgN_3_ (G) Au: Au_3_N (G), AuN_3_ (G), Au(N_3_)_3_ (G) **Group 12** Zn: Zn_3_N_2_ (G), Zn(N_3_)_2_ (G) Cd: Cd_3_N_2_ (G), Cd(N_3_)_2_ (G) Hg: Hg_2_N (G), Hg_3_N_2_ (G), Hg_2_N_4_ (G), HgN_3_ (G), HgN_6_ (G) **Group 13** B: B_3_N (G), BN (G) Al: Al_3_N (G), AlN (G), AlN_9_ (G) Ga: GaN (G) In: InN (F) Tl: Tl_3_N (G), TlN (F), TlN_3_ (G), Tl_2_N_12_ (G) **Group 14** C: C_2_N (G), C_3_N_2_ (G), (CN)_2_ (G), CN (G), CN_2_ (G), CN_4_ (G) Si: (Si_2_N_2_)_x_ (G), SiN (G), Si_3_N_4_ (G), Si_2_N_3_ (G), Si(N_3_)_4_ (G) Ge: Ge_3_N_2_ (G), Ge_3_N_4_ (G) Sn: Sn_3_N_2_ (G), Sn-N-films (F), Sn_3_N_4_ (G), Sn(N_3_)_4_ (G) Pb: Pb_3_N_2_ (G), Pb_3_N_4_ (G), Pb(N_3_)_2_ (G), PbN_9_ (G), PbN_10_ (G), PbN_11.5_ (G); α-Pb(N_3_)_2_ → PbN_3.25_ → PbN_2.85_ → PbN_2.78_ → PbN_2.53_ →PbN_2.5_ (G). **Group 15** P: PN (G), (PN)_n_ (G), P_4_N_6_ (G), P_3_N_5_ (G), (P_3_N_5_)_n_ (G), (PN(N_3_)_2_)_3_ (G) As: AsN (G) Sb: SbN (G), Sb(N_3_)_3_ (G), Sb(N_3_)_5_ (G) Bi: BiN (G) **Group 16** S: S_5_N_2_ (G), S_4_N_2_ (G), (S_2_N)_x_ (G), (SN)_x_ (G), S_4_N_4_ (G), S_2_N_2_ (G) Se: Se_4_N_4_ (G), SeN (G) Te: TeN (G), Te_3_N_4_ (G) **Lanthanides** CeN (G), CeN_2_ (G), PrN (G), PrN_2_ (G), Pr(N_3_)_3_ (G), NdN (G), NdN_2_ (G), NdN_9_ (G), SmN (G), EuN (G), GdN (G), TbN (G), DyN (G), HoN (G), ErN (G), TmN (G), YbN (G)

Chemical reactions at high pressures and high temperatures (HP-HT), performed during the past two and half decades, supported the expectation to find novel higher nitrides due to, most probably, increase of electronegativity of nitrogen with pressure. More precisely, high pressures appear to promote increase of the *relative* electronegativity differences [1] between nitrogen and metals participating in the reactions [6] and, in agreement with the basic concept of L. Pauling (see above), formation of higher nitrides where the metals attain higher oxidation state (for example, +4 instead of +3 for Ce and the group 14 elements) than at lower/atmospheric pressures. It has to be emphasised that oxidation state of an atom is defined, according to the *IUPAC Compendium of Chemical Terminology*, as the charge of this atom after ionic approximation of its heteronuclear bonds [1]. Increase of oxidation state of metals with pressure, also in their compounds with the group 15 elements, is a subject of research since more than six decades [7–12].

Most of the nitrides recoverable to ambient conditions were considered in several review articles [5,10,13–15] which, up to very recently, preferably dealt with their composition, structure and particular *P-T* conditions of the synthesis. Apart from a few exceptions, experimental *P-T* diagrams of these M-N systems and stability regions of the low- and high-pressure nitrides are incomplete. Similarly, there are only sporadic attempts to theoretically elaborate *P-T* diagrams of binary M-N systems [16–18]. There are also reports on existence of pernitrides, diazenides [5,15,19–21] and other N-rich compounds with the N:M-rations far above the stoichiometric ones e.g [22,23]. Such compounds are out of the scope of this review because they are either unrecoverable to ambient conditions or extremely sensitive to air and moisture or the stated compositions need independent confirmation.

One of the main criteria for an unambiguous validation of composition of a reaction product is satisfying one of the basic thermodynamic rules, namely LeChatelier’s principle. The principle states, in the most general form, that [24,25]:

if the equilibrium of a system is disturbed by a change in one or more of the determining factors (as temperature, pressure, or concentration) the system tends to adjust itself to a new equilibrium by counteracting as far as possible the effect of the change.

In the case of a chemical reaction at HP-HT conditions, this basic thermodynamic principle requires that volume of the reaction product must be smaller than that of the stoichiometric mixture of all reactants involved in the reaction. For example, one mole of c-Zr_3_N_4_ (a HP-HT compound considered below), under the *P-T* conditions of its formation from the elements, must occupy a smaller volume than the sum of the volumes of three moles of elemental zirconium, Zr, and two moles of molecular nitrogen, N_2_:

(1) $$v\left(c-Zr_{3}N_{4}\right)<3\cdot v\left(\mathrm{Zr}\right)+2\cdot v\left(N_{2}\right)$$

where v is given in the unit [volume/mole] (e.g. cm^3^/mol or similar). Because zirconium mononitride, ZrN, forms at atmospheric pressure and can also be used in a reaction to synthesise c-Zr_3_N_4_, LeChatelier’s principle requires validity for the alternative path at the *P-T* conditions of the reaction:

(2) $$v(c-Zr_{3}N_{4})<3\cdot v\left(\mathrm{ZrN}\right)+0.5\cdot v\left(N_{2}\right)$$

Unfortunately, validity of LeChatelier’s principle was not verified for most of the HP-HT nitrides with N:*M* > 1 reported in the literature. The stated stoichiometries were neither directly measured (using electron probe microanalysis, for example) nor verified to satisfy this basic thermodynamic principle. Finally, it should be mentioned that in the case of a first-order phase transition, without the stoichiometry change, LeChatelier’s principle simply requires that density of the high-pressure phase must exceed that of the low-pressure phase. For example, the transition from β-Si_3_N_4_ to γ-Si_3_N_4_ (see below) occurs when

(3) $$\rho (\gamma -Si_{3}N_{4})>\rho (\beta -Si_{3}N_{4})$$

Despite a progress in the basic characterisation of composition and structure of the novel binary nitrides as well as information about particular *P-T* conditions of their synthesis e.g [5,10,15,26,27], our knowledge about their properties is limited. This is surprising in view of numerous theoretical works predicting advanced mechanical, optoelectronic or catalytic properties of these nitrides. In a big majority of the existing publications on novel nitrides with N:*M* > 1, the known reliably measured properties are limited by their equation of state (EOS), ρ(*P*), and bulk modulus, *B*(*P*), extracted from the EOS e.g [15]. Fortunately, an EOS can be easily measured during decompression of the reaction product obtained, for example, via a reaction of the elements in a laser-heated diamond anvil cell (LH-DAC) [28]. The approach is very popular and simple and, for this reason, often used in the high-pressure domain. If structure of an examined product is correctly defined, such measurement provides reliable or, at least, preliminary value of bulk modulus for a densified sample at atmospheric pressure, *B*_0_, because the XRD measurements deliver volume of the crystallographic unit cell not affected by porosity, texture, contaminating phases or pressure transmitting medium (PTM). (Here and below, the subscript ‘0’ indicates parameters for an ideally densified solid at atmospheric pressure). However, the XRD measurements upon decompression from very high pressures can lead to systematically under- or overestimated *B*_0_–values if the first pressure derivative of bulk modulus at atmospheric pressure, *B*_0_´, is assumed but not confirmed to be constant, typically selected to be equal to 4 (see also below) [29]. Another property which is traditionally addressed is hardness of novel nitrides [15,28,30–35] because the HP-HT synthesis is industrially used for production of (super)hard solids such as diamond or cubic boron nitride (c-BN) e.g [36–38]. The ceased hype about the existence of a ‘C_3_N_4_ harder than diamond’ is an ample example of focusing on a single property [39–41]. In contrast, the HP-HT synthesis was demonstrated to produce high-quality single crystals of soft hexagonal boron nitride, h-BN, found to be a suitable substrate for the investigation of graphene and architectures based on it e.g [42,43]. On the other hand, reliable measurements of other mechanical and physical properties of the discovered nitrides remain rare despite a large number of theoretical works, mostly based on DFT calculations often combined with empirical or phenomenological approaches. Examples of such difficult-to-measure properties, even at atmospheric pressure, are shear modulus, *G*_0_, of a polycrystalline sample or single crystal elastic moduli, *C*_ij_, electronic structure including type and size of the band gap, refractive index etc. The main obstacles are, even in the case of polycrystalline samples, their small sizes, impurities, texture, and porosity not to mention the banal absence of single crystals with sizes sufficient for examination of dependences on crystallographic orientations. Some technical solutions proposed and applied to overcome these limitations as well as the obtained data are one of the subjects of the present work. Also, it is focused on those high-pressure nitrides whose thermodynamic stability has been confirmed at the *P-T* conditions of their formation (e.g. LeChatelier’s principle is fulfilled), whose composition could be verified experimentally, who are recoverable to ambient conditions, and metastable upon heating to sufficiently high temperatures. Compounds with potential for industrial applications were prioritised.

## 2. Three families of binary and ternary nitrides

### 2.1. High-pressure nitrides of the group 14 elements → spinel nitrides

Binary nitrides of the group 14 elements having cubic spinel structure (γ-M_3_N_4_ where *M* = Si, Ge or Sn) (Figure 1A) are the first family of HP-HT nitrides, after those of the group 13 elements (BN, GaN or InN) who attracted attention as technologically interesting materials. They were discovered almost simultaneously by two independent researcher teams from Germany [46–48] and a team from U.S.A. [49,50]. In a spinel-type structure (space group *Fd*-3*m*, no. 227), the cations occupy special sites without positional degrees of freedom of two types where they are either tetrahedrally (site 8*a*, (1/8,1/8,1/8)) or octahedrally (site 16*d* (1/2,1/2,1/2) coordinated by the N^3-^ anions. In contrast, positions of the anions (site 32*e* (*x*, *x*, *x*) in 1st setting) have to be recovered from structure refinement. Table 2 summarises experimental data on the cubic lattice parameter at ambient conditions, *a*_0_, and position of the anions (value of *x*) for all reported types of the spinel-nitrides.

**Figure 1.**
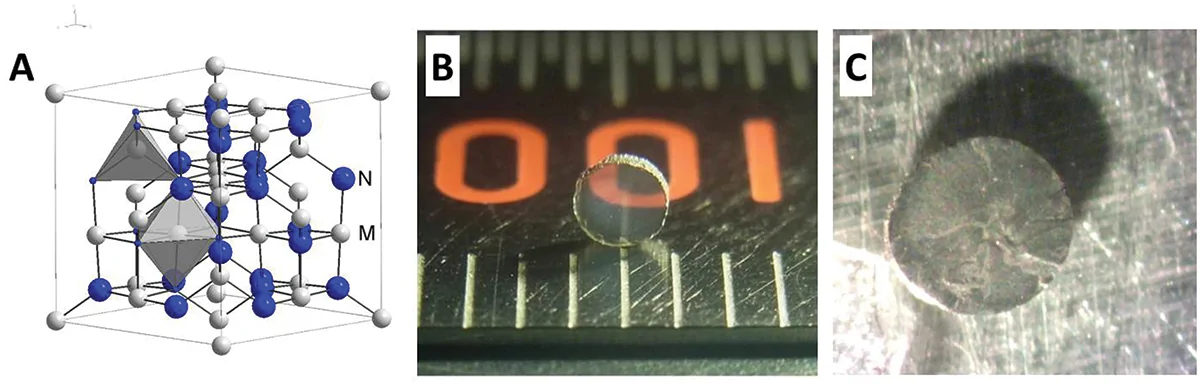
(A) Ball-stick model of spinel structure of nitrides of the group 14 elements, γ-M_3_N_4_ where M = Si, Ge, or Sn. Two types of coordination polyhedra of cations in spinel structure, tetrahedron and octahedron, are shown. (B) Photograph of a bulk transparent sample of nanocrystalline γ-Si_3_N_4_ synthesised by Nishiyama et al. At 15.6 GPa and 2073 K and reported in ref [35]. (C) Photograph of an almost completely densified bulk sample of γ-Ge_3_N_4_ of ~1.25 mm in diameter synthesised at ~13 GPa and ~1500 K [44,45]. Despite the low porosity of only 2.6%, the sample remained opaque in contrast to that of γ-Si_3_N_4_ shown in (B).

**Table 2.** Refined lattice parameters, *a*_0_, and positions of N-anions, *x*(N), in unit cells of spinel nitrides γ-M_3_N_4_ (where *M* = Si, Ge or Sn), at ambient conditions. The reported solid solutions γ-Si_3-z_N_4-y_O_y_ and γ-Si_3-u_Al_u_O_u_N_4-u_ are also included.

Compound	*a*_0_ (Å)	*x*(N)	References & comments
γ-Si_3_N_4_	7.80 (8) 7.7381 (2) 7.7339 (1) 7.742 (9) 7.7373 (6) 7.7318 (2) 7.744 (11) 7.75 (1)	<0.2625 0.25968 (1) 0.2584 (1) n. a. n. a. n. a. n. a. n.a.	[51] from the elements in a LH-DAC, TEM-SAED [52] MAA, pXRD [53] MAA, pXRD [54] [35] [55] [56] shock-wave, pXRD [57] shock-wave, pXRD
γ-Si_2.8_N_3.6_O_0.4_ γ-Si_3-z_N_4-y_O_y_	7.751 7.75	0.2565 (4) 0.26	[58] shock synthesis, pXRD [59] shock synthesis, <35 nm grains, TEM
γ-Si_2_AlON_3_ γ-Si_1.9_Al_1.1_O_1.1_N_2.9_	7.8234 (3) 7.836 (2)	0.2591 (2) n.a.	[32] [60]
γ-Ge_3_N_4_	8.30 (5) 8.2125 (1) 8.206 (2) 8.2048 (6)	<0.2625 0.2577 (1) n. a. n. a.	[47] from the elements in a LH-DAC, TEM-SAED [49,50] MAA, pXRD [61] shock synthesis, pXRD [45] MAA, pXRD
γ-Sn_3_N_4_	9.0187 (5) 9.037 (3) 9.03716 (5) ~9.074* 9.0144 (1) 9.0205 (5) 9.03716 (5) 9.029 9.0549 (2) 9.0221 (5) 9.01–9.05^$^	0.2575 (14) 0.25950 (6) n. a. n. a. n. a. n. a. n. a. n. a. 0.2600 (6) 0.2594 (4) 0.240–0.241^$^	[29] from the elements in a LH-DAC, XRD-Gandolfi [48] ammonolysis, neutron diffraction [62] ammonolysis,pXRD [63] NH_3_ vapour phase epitaxy, pXRD [64] HP-HT metathesis reaction, pXRD [65] HP-HT metathesis reaction, pXRD [62] precursor ammonolysis, pXRD [66] precursor ammonolysis, pXRD [67] solvothermal metathesis reaction, pXRD [68] metathesis reaction, scXRD [68] metathesis reaction, pXRD^$^

*average of *a*_0_ values calculated here for each of the reported interplanar distances.

^$^Summary of measurements for several products.

Synthesis of spinel-nitrides by different HP-HT techniques (see next chapter) was reported multiple times (see Table 2), including reactions of the elements [29,46,47]. γ-Si_3_N_4_ and γ-Ge_3_N_4_ are thermodynamically stable at static pressures above ~10 GPa e.g. [69,70] but the first synthesis experiments were performed at significantly higher pressures. Moreover, these spinel-nitrides persist in a broad range of pressures and temperatures, up to 98 GPa and ~2700 K, respectively [70]. At atmospheric pressure, these compounds appear as one of two hexagonal phases having almost identical densities, α- or β-Si_3_N_4_, and, α- or β-Ge_3_N_4_. In agreement with LeChatelier’s principle, densities of γ-Si_3_N_4_ and γ-Ge_3_N_4_ are, respectively, 26% and 19% higher than those of the hexagonal phases [46,47,49,50]. The latter phases are the most common starting materials for the HP-HT synthesis of macroscopic (porous or densified) bodies of γ-Si_3_N_4_ and γ-Ge_3_N_4_ in large-volume HP-HT apparatuses (Figure 1B,C) or of nanocrystalline powders via shock compression because these techniques require solid starting materials with the N:*M* ≥ 4:3. Short information about various synthesis routes can be found in Table 2.

In contrast to silicon(IV) and germanium(IV) nitrides, tin(IV) nitride is not accessible via nitridation of Sn at low pressures. It is still unclear whether Sn_3_N_4_ with any structure is thermodynamically stable at ambient conditions. In contrast, γ-Sn_3_N_4_ is thermodynamically stable at HP-HT conditions because it forms from the elements when heated in a laser-heated diamond anvil cell (LH-DAC) to *T* ~2300 K at *p* = 15.7 GPa and 20 GPa [29]. According to the literature, obtaining of a densified macroscopic sample of γ-Sn_3_N_4_ using large-volume apparatuses is a difficult task: There is only one report on successful synthesis of such a sample or rather spinel-oxynitride with the approximate composition γ-Sn_3-x_(N_0.8_O_0.2_)_4_, if the reported amount of oxygen of <3 wt.% [34] is taken into account and absence of an amorphous by-product presumed. This bulk sample was obtained via compression of the nanocrystalline powder of γ-Sn_3_N_4_ to *p* = 23 GPa and heating to *T* ≈ 1300 K while the starting material with a much lower oxygen content (O:(N+O) ≈ 3%) was earlier prepared at *p* ≈ 2.45 GPa and *T* ≈ 620 K via the metathesis reaction:

(4) $$3\cdot \mathrm{Sn}I_{4}+4\cdot Li_{3}N+x\cdot NH_{4}\mathrm{Cl}\to Sn_{3}N_{4}+12\cdot L\mathrm{iI}+x\cdot NH_{4}\mathrm{Cl}$$

where NH_4_Cl served as an absorber of heat released during the highly exothermic reaction [64]. It appears that the O-substitution is tolerated by the spinel structure up to O:(O+N) between 20% and 33% because a HP-HT product with composition close to Sn_2_N_2_O was reported to exhibit orthorhombic structure while a minor admixture of γ-Sn_3_(N,O)_4_ was recognised [71]. In that work, the authors synthesised the oxygen-containing Sn-N-O precursors from the Sn[NMe_2_]_4_, where Me = CH_3_, using a two-stage ammonolysis procedure earlier developed by Li et al. [62]. Another two-step process including, at the first step, synthesis of a gel-like tin-urea precursor and its ammonolysis at 700–800 K in the second step, led to nanocrystalline γ-Sn_3_N_4_ [66]. An approach of a metathesis reaction (see Equation 4) in the presence of a heat absorber was presented by Fitch et al. who used liquid benzene instead of solid NH_4_Cl to produce γ-Sn_3_N_4_ [67]. In a recent work, an inert gas served as absorber of heat released during the metathesis reaction of vaporised SnCl_4_ (diluted in the Ar-flow) with Li_3_N, Mg_3_N_2_ or Ca_3_N_2_ [68]. The authors succeeded to scale up the production to 1 g per batch and were able to reduce oxygen contamination to O:(O+N) ≤7% when Mg_3_N_2_ was used as the reactant. They also detected single crystals of γ-Sn_3_N_4_ up to 5 μm in size and, accordingly, were able to perform first structure refinement based on single-crystal XRD measurements. Finally, examination of the products via soft-X-ray spectroscopy suggested that incorporation of oxygen into the spinel-structure of tin(IV) nitride and formation of the charge-neutral *γ*-Sn_3-x_(N_1-x_O_x_)_4_ cannot be excluded [68].

Interestingly, γ-Sn_3_N_4_ was deposited as thin films almost one decade prior to the work of Scotti et a [48]. but not discovered because an incorrect structure solution, namely hexagonal, was proposed in the first and adopted in the subsequent works [72–74]. Texturing of the films, resulting in the absence of two XRD-peaks of moderate intensity (reflexes (400), (620) and/or (622) of the spinel structure) in the powder X-ray diffraction (pXRD) pattern, attributing of a single peak to the clearly visible doublet (the reflexes (533) and (622) of the spinel structure) at 2θ around 68° (see Figure 2) as well as insufficient statistics of the measured XRD-intensities appear to be at the origin of the misleading hexagonal structure solution.

**Figure 2.**
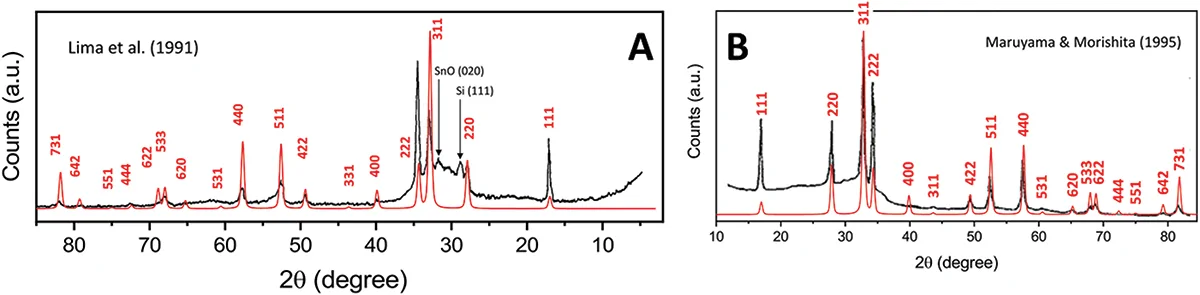
Experimental X-ray powder diffractograms (black lines) collected by (A) Lima et al. [72] and (B) Maruyama et al. [73]. Red lines represent calculated pXRD pattern for an ideal polycrystalline texture-free samples of γ-Sn_3_N_4_ having cubic spinel structure.

Formation of the solid solution γ-(Si_1-x_Ge_x_)_3_N_4_ with 0.18 ≤ x ≤ 0.88 at HP-HT conditions was reported more than 20 years ago [75] but, to our knowledge, not yet reproduced. Partial substitution of nitrogen by oxygen was recognised in γ-Si_3_N_4_ obtained via shock-wave synthesis [58,59] but quantitative measurement of elemental composition of such oxygen-bearing silicon nitride with spinel structure, γ-Si_2.8_N_3.6_O_0.4_, was performed in only one work [58]. Partial substitution of nitrogen by oxygen could also have taken place in γ-Si_3_N_4_ synthesised at static HP-HT conditions in multianvil apparatuses (MAAs). Incorporation of O-anions in the spinel structure was proposed to explain optical transparency of the recovered densified samples of γ-Si_3_N_4_ (Figure 1B) because it prohibits formation of triple pockets between the γ-Si_3_N_4_ crystallites where the inevitable contaminating compounds (e.g. SiO_2_) with refractive index different from that of the main matrix would accumulate [35]. If this were not the case, the sample would remain opaque. Authors of the same work also reported that a part of oxygen segregated in disordered/amorphous intergranular films composed of a silicon oxynitride but their thicknesses were very small, below 1 nm. If opacity of a densified sample is due to the presence of such triple pockets containing foreign admixtures then opacity of the almost-completely-densified samples of γ-Ge_3_N_4_ [45] suggests intolerance of its spinel structure to any detectable substitution of nitrogen by oxygen (Figure 1C). Transparency of γ-Ge_3_N_4_ to visible light was expected because it exhibits a direct band gap of *E*_g_ = 3.65(5) eV [44] (see below).

A distinct family of HP-HT compounds containing nitrogen and crystallising in spinel structure are the so-called SIALONs where the isoelectronic Al-O units substitute the Si-N units in the spinel structure, γ-Si_3-u_Al_u_O_u_N_4-u_. Such quaternary phases with 1.03 ≤ u ≤ 2.09 were reported to form at static pressures above 13 GPa if the starting samples of β-Si_3-u_Al_u_O_u_N_4-u_ are heated for short periods of time to temperatures around 2100 K [32,76]. Their cubic lattice parameters (Table 2) were found to increase with the amount of the Al-O units (quantified via the u-value) and follow the linear dependence [76]:

(5) $$a_{\text{0}}[A{}^{\circ}]\text{=7}.\text{7347}\left(\text{9}\right)\text{+0}.\text{0872}\left(\text{17}\right)\cdot \text{u}$$

However, these γ-Si_3-u_Al_u_O_u_N_4-u_ products decompose to mixtures of the corresponding binary compounds (SiO_2_, Al_2_O_3_, AlN and/or Si_3_N_4_) if heated for long time at the same *P-T* conditions [76,77]. This stepwise process agrees with the Ostwald-Volmer’s step rule e.g [78]. where γ-Si_3-u_Al_u_O_u_N_4-u_ should be considered as an intermediate metastable product forming prior decomposition of the heated at high pressures β-Si_3-u_Al_u_O_u_N_4-u_. γ-Si_3-u_Al_u_O_u_N_4-u_ could be recovered to ambient condition only because kinetics of the transformation is very sluggish. This could also explain availability of large amounts of nanocrystalline powders of γ-Si_3-u_Al_u_O_u_N_4-u_ with u = 0.9 and 1.4 via shock-wave synthesis at *p* > 33 GPa [79]. Examination of the Al *K* X-ray absorption near-edge structure (XANES) of the latter products and theoretical calculations suggested that Al-cations preferably occupy octahedral sited in the spinel structure and that their local environment is close to AlO_5_N and AlO_6_ [80].

### 2.2. High-pressure nitrides of the group 4 elements → Th_3_P_4_-type nitrides

After the discovery of the spinel-nitrides, γ-M_3_N_4_, extension of the search for novel binary high-pressure nitrides to the group 4 elements was obvious. And indeed, chemical reactions of the elements with molecular nitrogen at sufficiently high pressures and temperatures (see below) led to novel nitrides with cubic Th_3_P_4_-type structure, c-A_3_N_4_ where A = Ti, Zr, or Hf [28,81] (Figure 3(A), Table 3). In this structure (space group *I-43d*, no. 220), all cations occupy fixed positions, site 12*a* (3/8,0,1/4), while positions of all anions are of the same type, site 16*c* (*x*, *x*, *x*), but their coordinates have to be extracted from the structure refinement (Table 3). c-Zr_3_N_4_ and c-Hf_3_N_4_ are thermodynamically stable at HP-HT conditions, can be recovered to ambient conditions and remain metastable upon heating in air to ~800 K [28,82]. In contrast, c-Ti_3_N_4_ forms from the elements when heated to *T* > 2400 K at *p* ≈ 70 GPa but becomes unstable upon decompression at room temperature to *p* < 5 GPa [81].

**Figure 3.**
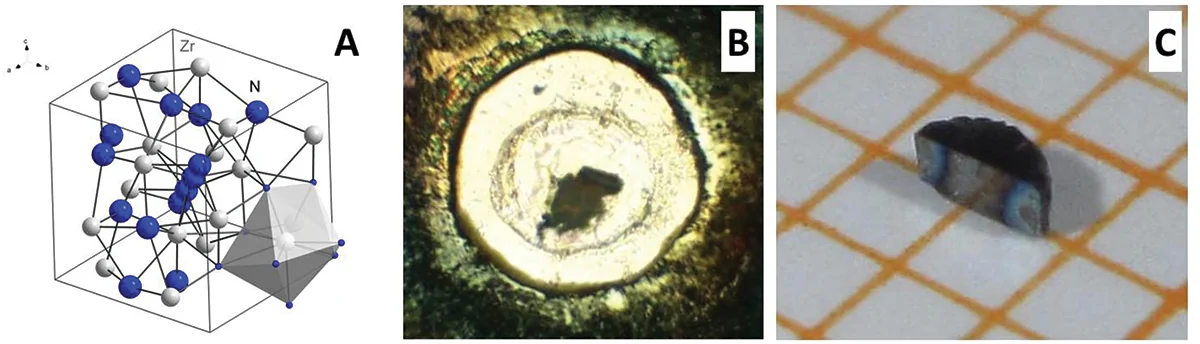
(A) Ball-stick model of cubic Th_3_P_4_–type structure of nitrides of the group 4 elements, c-A_3_N_4_ where A = Ti, Zr, or Hf. Only one type of the coordination polyhedron of cations exists in this structure. It can be described as an irregular octaverticon formed by eight nearest anions. (B) Photograph of the sample chamber in a LH-DAC showing product of reaction of the elements, hafnium and nitrogen, at 18 GPa and 2800 K [28]. Contrast and brightness of the photograph, but not colours, were modified in order to enhance central part of the product which is transparent to dark-red light. This indicated formation of semiconducting c-Hf_3_N_4_ with a narrow band gap. (C) Photograph of a sample of c-Zr_3_N_4_ synthesised in a MAA [82] showing metallic lustre. The initially cylindrical sample was cut radially. Bluish traces at edges of the plane surface appeared after applying high voltage to these sample areas.

**Table 3.** Refined cubic lattice parameters, *a*_0_, and positions of N-anions, *x*(N), in unit cells of nitrides of the group 4 elements having Th_3_P_4_–type structure, c-A_3_N_4_ (where A = Ti, Zr or Hf), at ambient conditions. Values obtained at high pressures are indicated.

Compound	*a*_0_ (Å)	*x*(N)	References & comments
c-Ti_3_N_4_	5.946 (1)^a^	0.07636^a,b^	[81] from δ-TiN and N_2_ in a LH-DAC
c-Zr_3_N_4_	6.740 (6)	0.063 (3)	[28] from the elements in a LH-DAC, pXRD
c-Zr_3_N_4_	6.7596^c^	n. a.	[83] metathesis reaction in a Belt-press
c-Zr_2.86_ (N_0.88_O_0.12_)_4_	6.7549 (1)	0.0661 (3)	[84] from *n*-Zr_3_N_4+x_ in a MAA, pXRD
c-Hf_3_N_4_	6.701 (6)	0.063 (2)	[28] from the elements in a LH-DAC, pXRD
c-Hf_3_N_4_	6.702 (1)	n. a.	[82] from *n*-Hf_3_N_4+x_ in a MAA, pXRD

^a^measured at *p* ≈75 GPa.

^b^derived from structural optimisation based on density functional theory (DFT).

^c^derived from the reported product density.

In the Th_3_P_4_-type nitrides, c-A_3_N_4_, metals are in the highest oxidation state +4, in contrast to the well-known mononitrides of the same metals crystallising in cubic NaCl-type structure, δ-AN. The latter compounds form upon heating of the elements in nitrogen atmosphere at low pressures. Existence of binary nitrides with Zr and Hf in the oxidation state +4 was reported earlier but they formed via a nonequilibrium process of ammonolysis of sublimated tetrachlorides (ZrCl_4_ or HfCl_4_) at high temperatures [85–87]. In such reactants, the metals are already in the oxidation state +4 and nitrogen in NH_3_ is highly reactive when compared with the inert N_2_ molecules. The low-pressure zirconium(IV) nitride exhibits orthorhombic structure, o-Zr_3_N_4_, and decomposes to δ-ZrN and N_2_ when heated to ~1100 K in nitrogen gas at atmospheric pressure [87]. In agreement with LeChatelier’s principle, density of o-Zr_3_N_4_ is smaller than that of the high-pressure c-Zr_3_N_4_ by ~13%. Similarly, specific volume of c-Hf_3_N_4_ was found to be smaller than that of the mixture 3 HfN +0.5 N_2_ at the synthesis pressure of ~18 GPa [28].

The research on HP-HT synthesis of macroscopic samples and examination of properties of c-A_3_N_4_ was constrained because loading of condensed nitrogen (liquid or solid) in large volume HP-HT apparatuses and the above-described element reactions were practically inaccessible. Also, low-pressure nitrides of the group 4 elements with N:A ≥ 4:3 are not commercially available and efforts were invested in the development of a reproducible, simple and scalable method of production of such nitrogen-rich reactants, amorphous or crystalline, with N:*M* ≥ 4:3. Li et al. described production of powders of nanocrystalline nitrides of zirconium and hafnium, *n*-A_3_N_4+x_, weakly contaminated with oxygen and carbon, via ammonolysis of the metal dialkylamides, i.e. A(NEt_2_)_4_, at atmospheric pressure and temperatures below 1000 K [88]. The obtained precursors exhibited rhombohedrally distorted NaCl-type structure which was previously reported for nitrogen-rich films of these nitrides only. These nanocrystalline powders were then used to synthesise macroscopic samples of c-A_3_N_4_ and to explore the *P-T* diagram of Zr-nitrides forming in excess of nitrogen. Even though the reactants had an insignificant excess of nitrogen, obtaining of completely densified bulk objects remained an issue. Using a MAA, bulk porous samples of oxygen-bearing zirconium(IV) nitride having Th_3_P_4_-type structure, c-Zr_3-u_(N_1-u_O_u_)_4_ with u = 0.12 were obtained at *p* = 12 GPa and *T* ≈ 1900 K [84] (Figures 3C and 4). Because the carefully measured elemental composition of the starting *n*-Zr_3_N_4+x_ showed much smaller amount of oxygen of 0.53(8) wt.% than in the high-pressure c-Zr_2.86_(N_0.88_O_0.12_)_4_, namely 2.4 wt.%, the authors concluded that during heating at high pressures the starting material did not only transform to the Th_3_P_4_-phase but also gradually oxidised via substitution of nitrogen by oxygen. Apparently, the oxidic environment of the sample assembly in a MAA (see below) served as a source of additional oxygen and the used Pt-capsule was not sufficiently strong barrier against oxygen diffusion inside the sample volume. The high-quality pXRD measurements showed that the substitution of nitrogen by oxygen led to the formation of cation vacancies which compensated the difference in the number of electrons attracted by N- and O-anions and kept the oxidation state of all Zr-cations equal to +4 [84]. Analogous experiments on the HP-HT synthesis of macroscopic samples of c-Hf_3_N_4_ in a large-volume HP-HT apparatus showed, in contrast, that its Th_3_P_4_-type structure does not tolerate any substitution of nitrogen by oxygen [82]: Oxygen, present in the starting *n*-Hf_3_N_4+x_ as well as that diffused from the surrounding HP-HT assembly into the reaction volume, was accumulated in a contaminating crystalline hafnium oxide or oxynitride which nature, however, could not be determined unambiguously due to low intensities of the corresponding XRD peaks.

**Figure 4.**
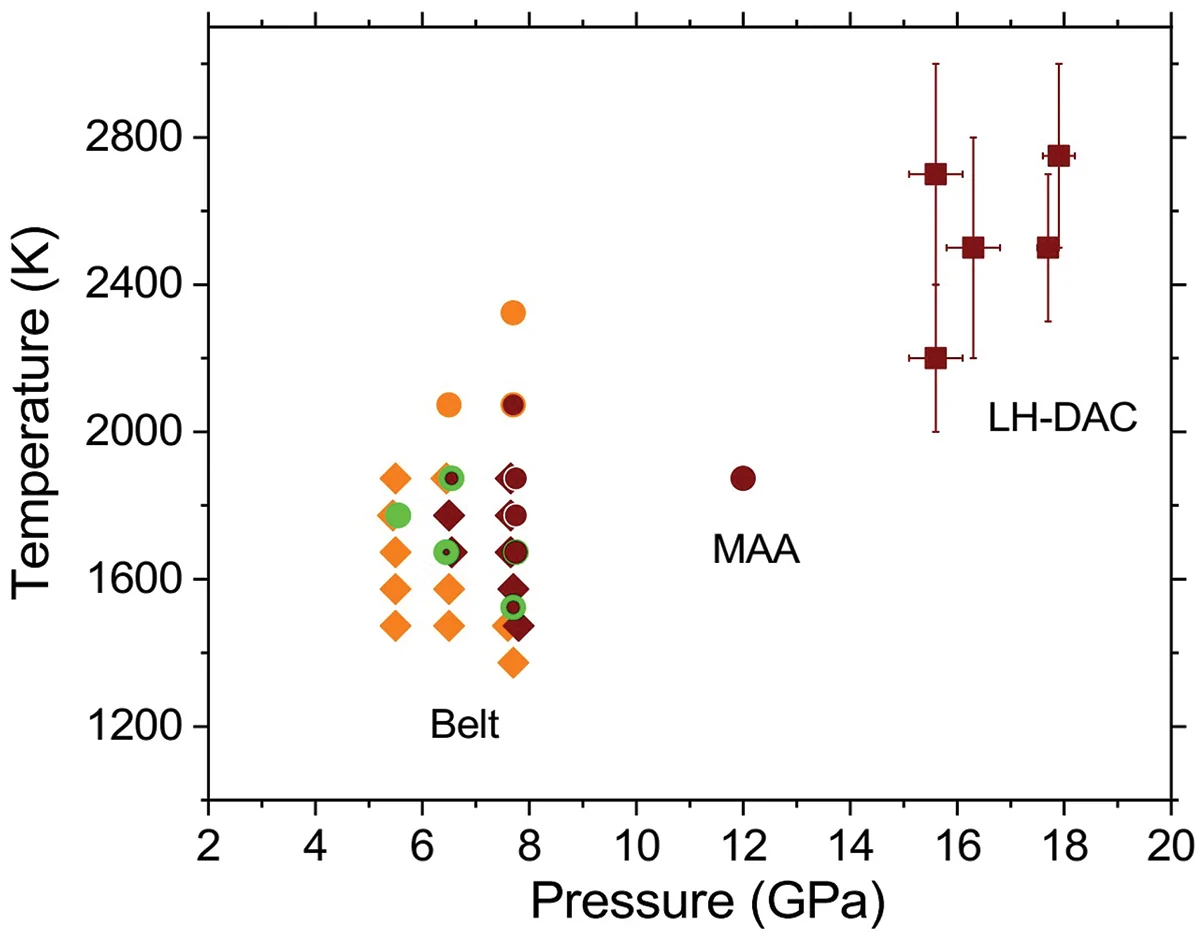
*P-T* conditions of formation of three compounds in the Zr-N system: c-Zr_3_N_4_ (wine symbols), ls-Zr_x_N_y_ (green symbols), and δ-ZrN (orange symbols). Squares indicate synthesis via reaction of the elements [28]; circles – via decomposition of nanocrystalline *n*-Zr_3_N_4+x_ [82,89]; diamonds – via metathesis reaction of ZrCl_4_ with Ca_3_N_2_ [83]. Bicoloured symbols indicate *P-T* conditions where two different phases were found to form while areas occupied by each colour show qualitatively amount of the corresponding compound. If synthesis conditions were the same in different experiments, the symbols were shifted by 0.5 GPa to right, for a high-pressure phase, and to left, for a low-pressure phase.

The powder of *n*-Zr_3_N_4+x_ was also used in the first work on the *P-T* phase diagram of zirconium nitrides forming when nitrogen is available in excess. The experiments were performed in a Belt-type apparatus (BTA) providing an industrial-scale sample volume [89]. The results showed that c-Zr_3_N_4_ and, most probably, c-Hf_3_N_4_ is thermodynamically stable at pressures between 6.5 GPa and 8 GPa and temperatures between ~1600 and ~2000 K (Figure 4) thus indicating that the industrial-scale production of c-Zr_3_N_4_ is feasible. Also, the authors reported appearance of a crystalline phase with a structure having low-symmetry, ls-Zr_x_N_y_, when *n*-Zr_3_N_4+x_ was heated to 1700–1850 K at *p* ≤ 6.5 GPa. According to preliminary unpublished measurements, this phase contains less nitrogen than c-Zr_3_N_4_, i.e. N:Zr < 4:3. At *p* > 6 GPa and *T* > 2050 K, the starting material decomposed to δ-ZrN [89].

Recently, a Japanese team reported on a direct synthesis of c-Zr_3_N_4_ at HP-HT conditions via a metathesis reaction:

(6) $$3\cdot \mathrm{ZrC}l_{4}+2\cdot Ca_{3}N_{2}\to Zr_{3}N_{4}+6\cdot \mathrm{CaC}l_{2}$$

The reactions were ignited at pressures between 5.5 GPa and 7.7 GPa and temperatures between 1373 K and 1873 K [83] (see Figure 4). These *P-T* conditions are overlapping with the range examined in the above considered work were *n*-Zr_3_N_4+x_ was used as the starting material [89]. The authors have tested several other combinations of the reactants, ZrF_4_ and ZrCl_4_, serving as the Zr-source and NaN_3_, Li_3_N, and Ca_3_N_2_, serving as the N-sources, but didn’t obtain c-Zr_3_N_4_. Compared with the previous result, the mildest pressure of formation of pure c-Zr_3_N_4_ decreased from 7.7 GPa to 6.5 GPa which are suitable for an economic industrial HP-HT synthesis of this material and, as a result, testing its tribological properties. Interestingly, synthesis via the metathesis reaction didn’t result in formation of the ls-Zr_x_N_y_ but, at the same *P-T* conditions, of c-Zr_3_N_4_ or δ-ZrN, even though the amount of nitrogen was sufficient for the formation of a compound with N:*M* = 4:3. One of the possible explanations is that ls-Zr_x_N_y_ is, according to Ostwald-Volmer’s step rule, an intermediate metastable stage of the decomposition reaction from c-Zr_3_N_4_ to δ-ZrN and nitrogen. In the case of the constructive metathesis reaction performed by Kawamura et al. [83], such metastable product could not appear and, accordingly, observed. However, the driving force of the decomposition and nitrogen release needs further clarification. It is possible that nitrogen has found a path to escape from the high-pressure volume and partial nitrogen pressure inside the capsules dropped thus promoting further decomposition of c-Zr_3_N_4_. It is imaginable that at temperatures close to melting [90], platinum (used as the capsule material) in Ref. [89] didn’t seal the sample volume and nitrogen could diffuse through the capsule walls. In the work of Kawamura et al. [83], molybdenum having much higher melting temperature was used and the diffusion through the capsule walls appears to be improbable.

### 2.3. High-pressure nitrides of the group 5 elements → U_2_S_3_-type nitrides

After the discovery of nitrides of the group 4 elements with N:A well above one, namely c-A_3_N_4_ where A = Ti, Zr or Hf, the search for novel high-pressure nitrides was extended to those of the group 5 elements. Several nitrides of these elements with various N:E > 1 (where E = V, Nb or Ta) were already reported in the literature (see Table 1 for those known in the past century) but their thermodynamic stability at atmospheric pressure was not clear: Reactions of the metals with excess of nitrogen at high-temperatures do not result in anything higher than mononitrides, EN [91,92]. Most of the nitrides with N:E > 1 were obtained via non-equilibrium processes such as thin-film deposition e.g [93,94], ammonolysis e.g [95–97]. or decomposition of a nitride with a higher N:E ratio then in the product e.g [98]. The most prominent member of the low-pressure nitrides with N:*M* > 1 appears to be orthorhombic Ta_3_N_5_ where all cations are in the highest oxidation state +5. As above, it can be obtained via ammonolysis of different precursors (for example, TaCl_5_ or Ta_2_O_5_) at high temperatures [95,96,99,100]. For example, Ta_3_N_5_ forms via a reaction of Ta_2_O_5_ with NH_3_ at temperatures up to 1173 K [99,101]. However, this compound should be considered as thermodynamically metastable because it decomposes when heated to a lower temperature of *T* = 1123 K even though embedded in nitrogen-gas compressed to 105 Pa [99]. Ta_3_N_5_ attracted some attention as a catalyst for the photoelectrochemical (PEC) splitting of water and production of ‘green’ hydrogen without any noticeable corrosion [102–104].

As is the case for the group 4 elements, high pressures favoured formation of nitrides of the group 5 elements with N:E > 1: The metathesis reaction of TaCl_5_ and Ca_3_N_2_, mixed in the ratio needed to synthesise Ta_3_N_5_, at *T* ~2000K and *p* = 3 GPa led to the hexagonal Ta_5_N_6_ and a partial release of nitrogen [105]:

(7) $$6\cdot \mathrm{TaC}l_{5}+5\cdot Ca_{3}N_{2}\to \;\left(6/5\right)\cdot Ta_{5}N_{6}+15\cdot \mathrm{CaC}l_{2}+\left(7/5\right)\cdot N_{2}↑$$

This reaction can be considered as formation of Ta_5_N_6_ in excess of nitrogen and, accordingly, the obtained compound should be considered as thermodynamically stable at the synthesis conditions. Crystal structure of the hexagonal Ta_5_N_6_, as well as of tetragonal Ta_4_N_5_, was examined long time ago using samples obtained via chemical reaction of the evaporated metal with ammonia e.g [106]. The same synthesis approach led to the isomorphous Nb_4_N_5_ and Nb_5_N_6_ [107].

Further pressure increase led to the formation of a tantalum nitride with previously not reported orthorhombic U_2_S_3_-type structure, η-Ta_2_N_3_, (Figure 5). This phase was result of decomposition of Ta_3_N_5_ after heating to 1800–2000 K at pressures between 11 GPa and 20 GPa [108]. The structure was derived from powder XRD- and selected area electron diffraction (SAED) patterns while the requirement that the product satisfies LeChatelier’s principal was taken into account. In the U_2_S_3_-type structure (space group *Pnma*, no. 62), Ta-cations occupy, in the unit cell, two different positions of the same type, 4*c* (*x*, 1/4, *z*), while N-anions occupy three different positions of the same type, site 4*c* (*x*, 1/4, *z*). For all atoms, the *x*- and *z* coordinates were extracted from quantitative pXRD measurements providing reliable relative intensities of the diffraction peaks (Table 4). Quantitative chemical analysis of the product using electron probe microanalysis (EPMA) revealed partial substitution of nitrogen by oxygen in the U_2_S_3_-type structure. Accordingly, composition of this oxygen-bearing nitride was described as η-Ta_2-2/3x_(N_1–x_,O_x_)_3_, where 0 ≤ x ≤ 0.05 [108]. Considering the above cases of Ta_5_N_6_ and Ta_4_N_5_, formation of η-Ta_2_N_3_ could be interpreted, according to Ostwald-Volmer’s step rule, as a metastable intermediate product of decomposition of Ta_3_N_5_ to nitrogen and Ta_5_N_6_ or other nitride with N:Ta < 3:2. However, η-Ta_2_N_3_ was also synthesised from the elements heated in a LH-DAC to temperatures between 1400 K and 3700 K at pressures between 9 and 59 GPa [110,111]. This result confirmed that η-Ta_2_N_3_ is thermodynamically stable at the indicated *P-T* conditions, at least, while the upper pressure limit of its stability (and potential formation of a Ta_3_N_5_ with unknown structure) is far above the pressure permitting production of macroscopic samples in large-volume HP-apparatuses, not to mention *P-T* range of the industrial synthesis. Contrary to the above results, a brief report about coexistence of high-pressure phases of Ta_3_N_5_ with U_3_Se_5_- and U_3_Te_5_-type structures can be found in the literature [112]. These phases were reported to have appeared after the heating of a solid amorphous precursor with the nominal composition Ta_3_N_5_ in a LH-DAC to ~1500–2000 K at *p* = 20 GPa. Because combustion analysis showed a significant amount of carbon, C/(N+C)~7%, in the precursor and amount of oxygen was not quantified, role of the two elements in stabilisation of the U_3_Se_5_- and U_3_Te_5_-type structures of Ta_3_N_5_ needs further examination.

**Figure 5.**
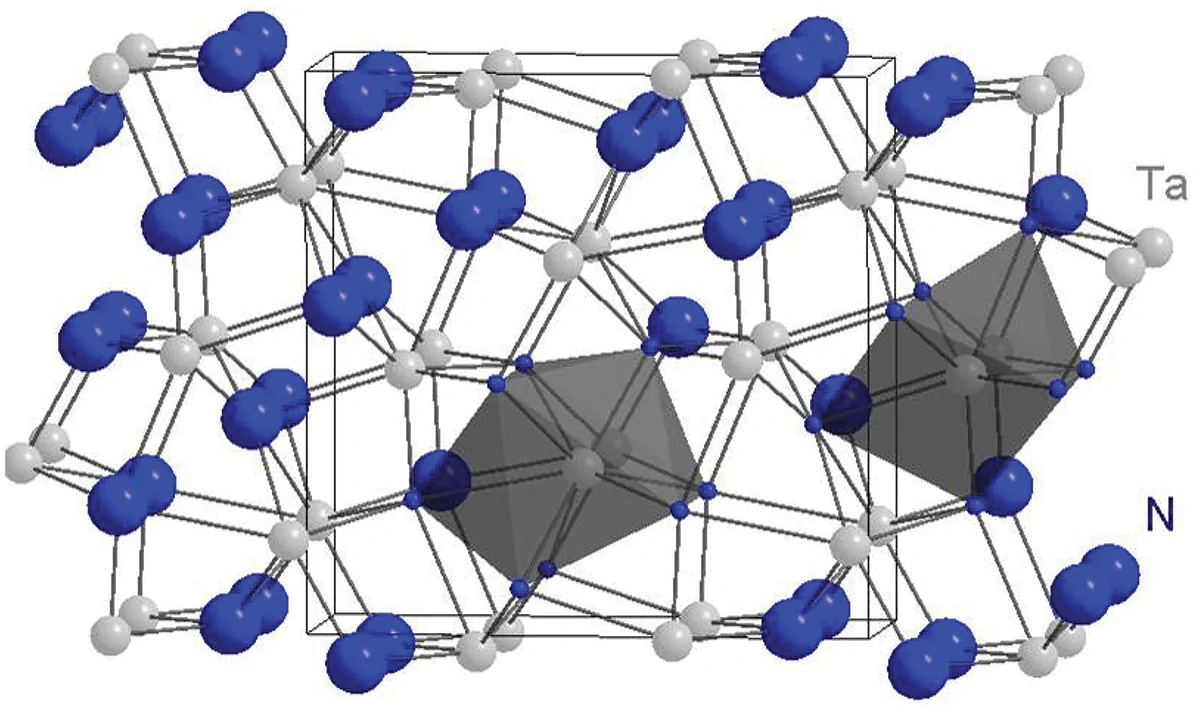
Ball-stick model of orthorhombic U_2_S_3_-type structure of HP-HT nitrides of the group 5 elements, η-E_2_N_3_, where E = Nb or Ta. The coordination polyhedra of the cations can be described as trigonal prisms while either one or two of the three prism faces are capped by N-anions [108].

**Table 4.** Refined lattice parameters, *a*_0_, *b*_0_, *c*_0_, and positions of cations and anions in unit cells of nitrides of the group 5 elements having orthorhombic U_2_S_3_-type structure.

Compound	Lattice parameters (Å)	Sites 4*c* Atom *x z*	References & comments
η-Nb_2_N_3_	*a*_0_ = 8.2056 (2) *b*_0_ = 2.9836 (1) c_0_ = 8.1328 (1)	Nb(1) 0.0213 (1) 0.6871 (1) Nb(2) 0.1940 (1) 0.0046 (1) N(1) 0.4468 (9) 0.377 (1) N(2) 0.1183 (9) 0.450 (1) N(3) 0.280 (1) 0.704 (1)	[109] From the elements in a LH-DAC, pXRD
η-Ta_2_N_3_	*a*_0_ = 8.1830 (17) *b*_0_ = 2.9823 (3) *c*_0_ = 8.1911 (17)	Ta(1) 0.0198 (4) 0.6873 (4) Ta(2) 0.1927 (4) 0.0046 (4) N(1) 0.475 (6) 0.375 (6) N(2) 0.110 (6) 0.415 (6) N(3) 0.252 (6) 0.701 (7)	[108] MA synthesis pXRD-Gandolfi, TEM
η-Ta_2_N_3_	*a*_0_ = 8.1804 (8) *b*_0_ = 2.9683 (2) *c*_0_ = 8.1474 (8)	n.a.	[110] Synthesis from the elements in a LH-DAC, pXRD

One decade after the discovery of η-Ta_2_N_3_, Asano et al. reported on synthesis of niobium nitride with U_2_S_3_-type structure, η-Nb_2_N_3_, via chemical reaction of δ-NbN with molecular nitrogen at high temperatures and pressures above ~30 GPa [109]. Powder XRD patterns of the sample recovered to ambient conditions were used to extract its structure, including coordinates of the cations and anions in the unit cell (Table 4). Very recently, formation of η-Nb_2_N_3_ via reaction of the elements after laser heating at pressures between 56 GPa and 86 GPa confirmed thermodynamic stability of this solid at high pressures and temperatures [113].

To our knowledge, existence of a vanadium nitride with the stoichiometry V_2_N_3_ was not reported in the literature (see also Table 1). In contrast, chemical reaction of metallic vanadium with nitrogen heated in a LH-DAC at *P* ~20–30 GPa leads to δ-VN [114]. Furthermore, the authors reported that the mononitride coexists with the pernitride VN_2_ after heating in excess of nitrogen at *P* ~73–85 GPa. It was stated that the pernitride exhibits CuAl_2_-type crystal structure.

## 3. HP-HT synthesis methods and their limitations

This chapter provides a brief overview of HP-HT techniques that enable the synthesis of samples composed of grains/crystallites whose surfaces contribute much less to the material properties than their interior. In particular, it is focused on the techniques of laser heated diamond anvil cell (LH-DAC) and large-volume HP-HT devices such as Belt-type apparatus (BTA) or multianvil apparatus (MAA). However, only those technical details will be considered that exclude or significantly reduce contamination of the samples with foreign phases and/or elements and thus ensure reliable measurements of the properties of interest and their association with the HP-HT phase/compound with a verified structure and composition. Readers interested in details of these techniques and various set-ups, starting from different types of DACs or large-volume modules, heating of samples in a LH-DAC or in MAA, measurement of pressures and temperatures, are referred to the original and review papers mentioned below.

### 3.1. Laser-heated diamond anvil (LH-DAC)

LH-DAC appears to be the technique of choice when synthesis of pure nitrides (free of oxygen) is aimed provided the below considered precautions are taken into account. Reviews of the LH-DAC technique can be found elsewhere e.g [27,115–122]. Basic elements of a LH-DAC is a diamond anvil cell (DAC) and radiation of a powerful IR-laser focused on the sample in a DAC (Figure 6). The set-up can be extended by a spectroscopic unit to measure temperature using thermal radiation of the heated sample. Basic elements of a DAC are two conically shaped diamonds (e.g. brilliant cut) with flat culets of 0.1–1.0 mm in diameter (Figure 6A). A hole in a pre-indented gasket (typically ~60 μm in thickness) represents the reaction volume containing two or more reactants or a low-pressure phase imbedded in a pressure transmitting medium (PTM). Transparency of diamond to light in a broad spectral range permits heating of samples with the focused radiation of an IR-laser, measurement of pressure using, for example, the R1-fluorescence line of ruby and *in-situ* sample analysis using different spectroscopic techniques (e.g. Raman) and/or X-ray diffraction (XRD).

**Figure 6.**
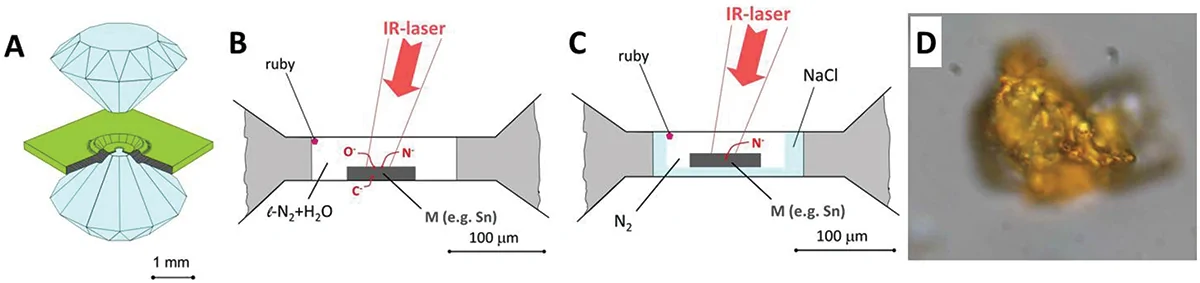
(A) Schematic of a DAC showing two opposed diamond anvils and a pre-indented gasket with a hole in the indentation centre. (B-C) Schematic cross-section of the sample assembly in the reaction volume of a DAC heated with focused IR-radiation of a Nd:YAG- or CO_2_ laser. The sample is either (B) placed on the bottom anvil and embedded in the cryogenically-loaded nitrogen contaminated with H_2_O–ice or (C) isolated from the bottom anvil by a NaCl-layer who precludes diffusion of carbon from the anvil into the sample and imbedded in pure nitrogen condensed using a gas-loader. (D) Optical image of a sample of δ-TiN with lateral size of ~20 μm obtained after the reaction of metallic Ti with N_2_ at 22 GPa and 2850 K, see also [28].

The simplest arrangement of reactants for a nitride synthesis is a metal disk (e.g. Sn, Zr or Ta), on one of the culets, in contact with the cryogenically-loaded liquid nitrogen (*l*-N_2_) (Figure 6B). Such arrangement provokes, however, the sample contamination via two paths: (1) The reaction product can be heavily contaminated with oxygen present in *l*-N_2_ in form of dispersed nano- or micro-grains of H_2_O-ice. This is because the air humidity condenses near the *l*-N_2_ surface to ice grains which sink into the liquid because the H_2_O-ice density is higher than that of *l*-N_2_. Convection of the boiling *l*-N_2_ promotes distribution of the ice grains throughout the whole volume and, as a result, a suspension of H_2_O-ice in *l*-N_2_ forms. Apparently, the H_2_O-ice concentration increases with the duration of exposure of *l*-N_2_ to air and achieves extreme values for the industrial *l*-N_2_. Upon the laser heating in a LH-DAC, the H_2_O-ice grains preferably react with the starting metal and thus lead to formation of contaminating oxides and/or oxynitrides, e.g. Sn_3-x_(N_1-x_O_x_)_4_ with eventually spinel structure, Sn_2_N_2_O with orthorhombic structure [71] or even to SnO_2_. As already mentioned above, the substitution of nitrogen with oxygen was documented in several studies on high-pressure nitrides.

Also, it has been verified that heating of a sample in contact with a diamond anvil leads to a strong contamination of the reaction product with carbon [123–125]. Interestingly, if a starting metal foil is placed directly on one of the diamond anvils and surrounded by the cryogenically loaded suspension of H_2_O–ice in *l*-N_2_ then both carbon from the diamond anvil and oxygen from the H_2_O-ice can participate in the reaction with the metal and formation of a carboxynitride, via the substitution of the N-N species by the isoelectronic C-O species, is very probable. In this case, lattice parameter and intensities of XRD peaks of a reaction product with spinel structure, e.g. γ-Sn_3_(N_1-2x_(CO)_x_)_4_, will be indistinguishable from those of γ-Sn_3_N_4_. Only quantitative analysis via electron-probe microanalysis (EPMA) would allow distinguishing of these compounds. However, measurement of composition of a compound composed of elements with a strong contrast in atomic weights is a very difficult task even for *binary* compounds, such as γ-Sn_3_N_4_ or η-Ta_2_N_3_ [108], not to mention the mentioned Sn-C-N-O compounds. It should be also noted that chemical compositions extracted from the energy-dispersive X-ray (EDX) spectra are not reliable.

The described contamination sources can be excluded if the following precautions are implemented: (i) Starting flake/disk of a metallic reactant should be taken from freshly cleaned/scratched surface of a bulk piece of this metal. (ii) If starting material is not an elemental metal but, for example, powder of a mononitride or of a low-pressure phase, chemical analysis prior to the synthesis is mandatory in order to exclude presence of oxides or oxynitrides. The metallic reactant should be isolated from the bottom diamond anvil by a layer of dry NaCl (or another inert solid) e.g [28]. (Figure 6C) and the remaining reaction volume should be filled with high-purity N_2_ using a gas-loader which condenses the gas directly from a gas-bottle into the vessel containing the DAC. It must be also verified that after the sealing of the condensed N_2_ in the DAC and compression to the desired pressure, thickness of the solid N_2_ above the metallic reactant is significant, This would preclude contamination of the product with carbon from the opposite/upper diamond anvil.

Another challenge by the structural characterisation of the products obtained in a LH-DAC is a small sample containing relatively big crystallites. This makes 2D-XRD patterns collected from such a sample nearly useless for the structure refinement due to the domination of strong diffraction spots from individual crystallites around the optimal diffraction 2θ-angles of an ideal pXRD pattern, (Figure 7A,B). Even though, the 2D-XRD pattern in Figure 7A contains more-or-less continuous rings or arcs, integration of different sections of this pattern resulted in distinct diffractograms where some of the XRD-peaks artificially split in two, three of even four peaks, depending on the selected section (Figure 7B).

**Figure 7.**
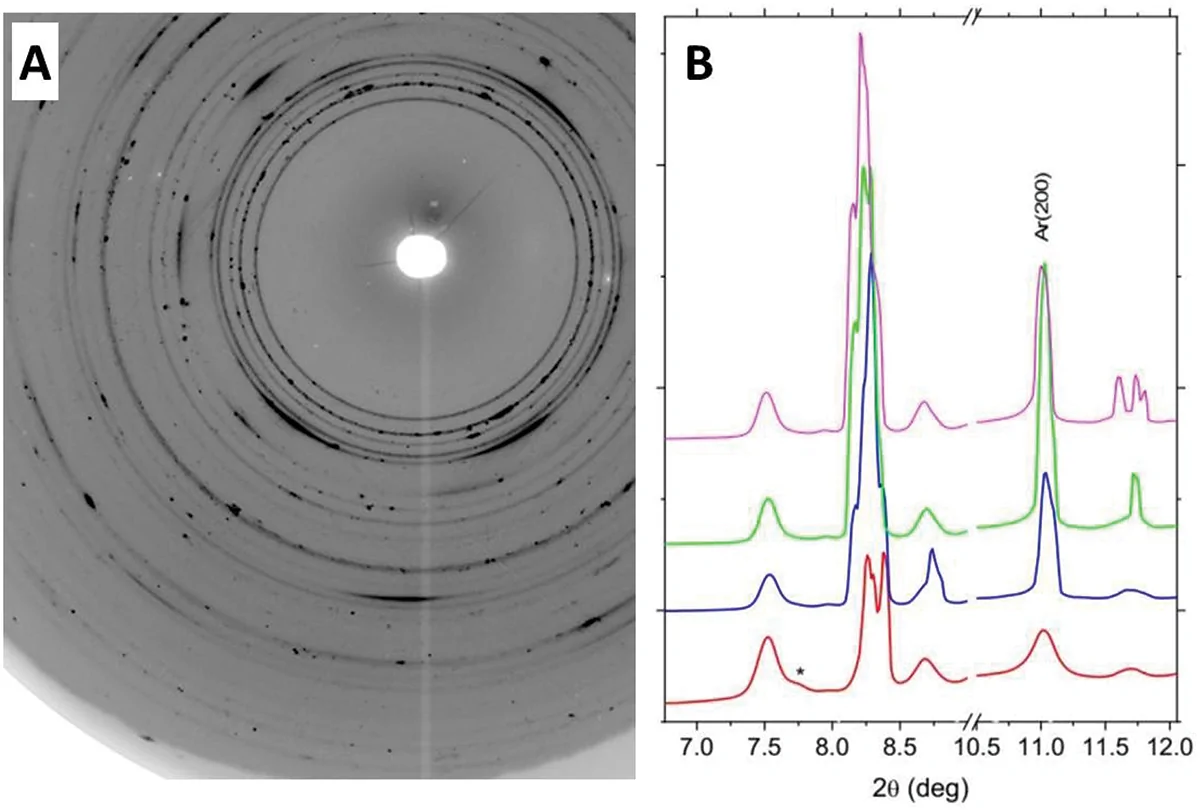
(A) 2D-XRD pattern from a stationary sample of a post-spinel phase of tin nitride, imbedded in the argon PTM, collected at *p* = 55 GPa using a monochromatic X-ray beam. This new phase was obtained after the laser heating of γ-Sn_3_N_4_ in a DAC to ~2500 K and ~50 GPa. (B) Red line shows a pXRD diffractogram, *I*(2θ), of γ-Sn_3_N_4_ obtained via integration of the 2D-XRD pattern shown in (A). Other lines show diffractograms obtained via integration of different sections of this pattern. Reproduced by permission from Supplemental material of the article: Hong C., Deng Y., Xu F., Huang H., Zerr A. *Phys. Rev. B*, **111**, 184,110 (2025) [126]. DOI: https://doi.org/10.1103/PhysRevB.111.184110. Copyright 2025 by the American physical society.

The most promising way to obtain powder diffractograms with correct intensities from such small samples is their rotation and precession around two nonparallel axes using a Gandolfi stage [127]. This device (Figure 8C) insures, even for a single crystal, generation of an ideal 2D-XRD pattern with continuous diffraction rings (Figure 8B). Such diffractograms permit not only refinement of crystal structure for a sample recovered from a LH-DAC or a large-volume HP-HT apparatus [28,29,32,108,128] but also quantitative analysis of each crystalline phase/compound present in the product, if any. It has to be noted that a multiaxis oscillation system providing similarly smooth 2D-XRD patterns from samples compressed in a DAC is available [129]. Another approach requiring, however, presence of a larger single crystal in a polycrystalline product and a high-brilliance X-ray source promises extraction of crystal structure if applied to samples after their recovery [130].

**Figure 8.**
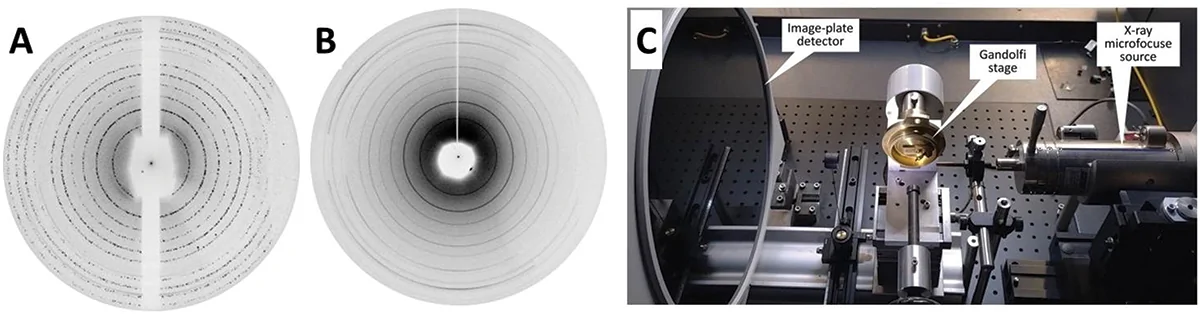
(A-B) 2D-XRD patterns of a powder sample of LaB_6_ containing a small number of grains within the volume irradiated with the Mo *K*α radiation collimated to a spot of ~150 μm in diameter (Incoatec GmbH, Germany). The pattern was collected using a MAR345 image-plate detector (marXperts GmbH, GERMANY). The patterns were obtained for (A) the stationary sample, (B) the sample rotated on a Gandolfi stage. Reproduced Figures (A) and (B) by permission from Supplemental material of the article: Hong C., Deng Y., Xu F., Huang H., Zerr A. *Phys. Rev. B*, **111**, 184,110 (2025) [126]. DOI: https://doi.org/10.1103/PhysRevB.111.184110. Copyright 2025 by the American physical society. (C) Photograph of the experimental set-up used to collect the XRD patterns shown in (A) and (B).

### 3.2. Large-volume HP-HT apparatuses

Large volume HP-HT apparatuses are devices permitting synthesis of macroscopic samples with sizes between ~0.5 mm and several millimetres. Such samples permit detailed characterisation (structure, composition etc.) and measurement of physical properties of the compounds discovered using a LH-DAC. However, the *P-T* conditions accessible using such apparatuses limit the repertory of novel materials which can be examined and subsequently proposed for industrial synthesis. Detailed descriptions of the most relevant HP-HT devices, such as Belt-type apparatus (BTA) and multi-anvil apparatus (MAA) (Figure 9), can be found elsewhere [132–139]. Here, only the details relevant to the synthesis of oxygen-free samples of binary and/or ternary nitrides will be considered.

**Figure 9.**
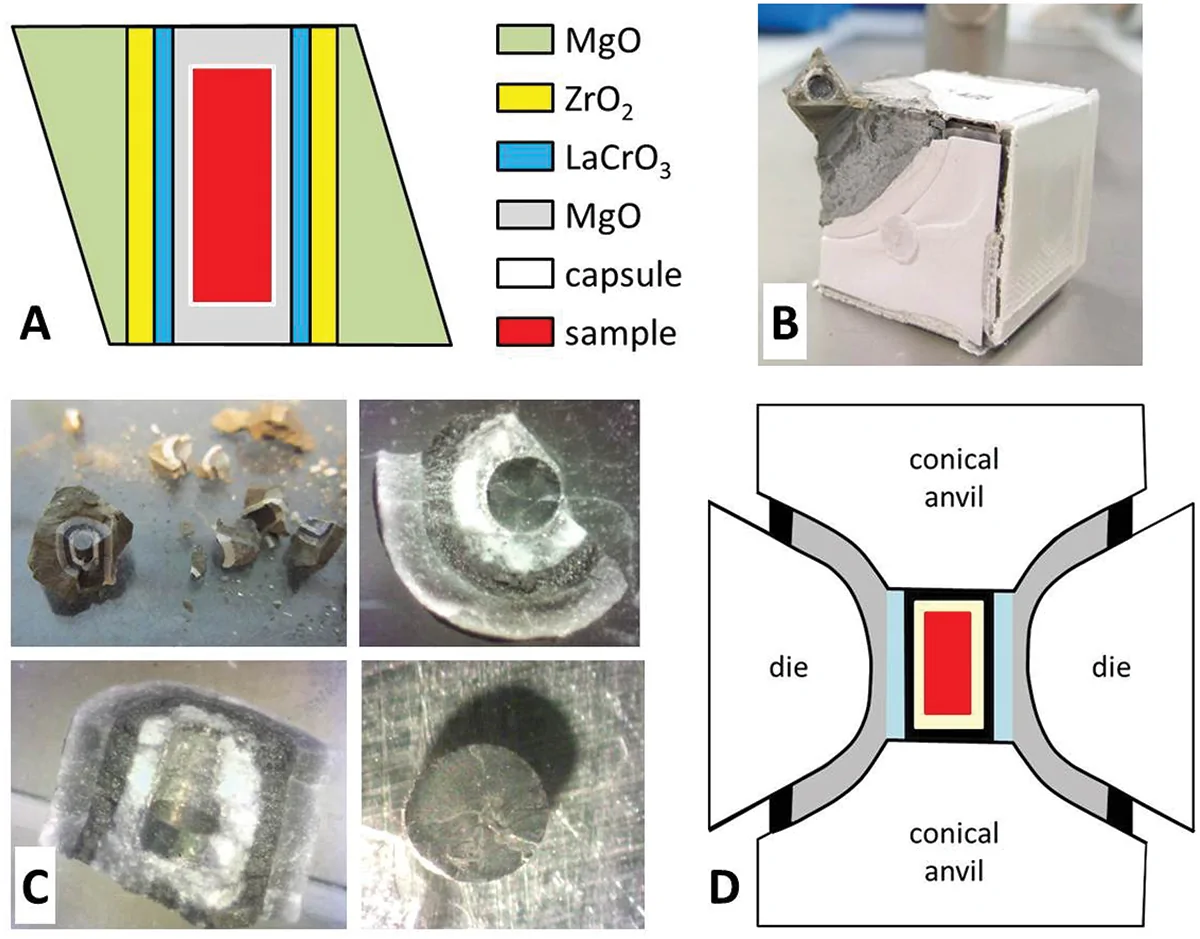
(A) Schematic cross-section of a simplified sample assembly inside an MgO octahedron which was used for the synthesis/densification of the novel nitrides in a MAA. The MgO octahedron is squeezed between eight truncated cubes made of cemented carbide (WC-Co composite described in more detail elsewhere [131]) who serve as anvils in a MAA; (B) Photograph of an MgO octahedron after the HP-HT synthesis experiment sticking to one of the anvils. The grey material on the left upper corner of the cube is remnants of pyrophyllite gaskets. (C) Recovery of a sample of γ-Ge_3_N_4_ from such a MgO-octahedron. (D) Schematic cross-section of the central part of a BTA composed of two opposed anvils (modified pistons) and a die (modified cylinder) made of cemented carbide. This simplified example of the sample assembly in a BTA includes pyrophyllite gaskets (grey); pressure medium, e.g. Al_2_O_3_ + NaCl (blue); graphite heater (black); pressure medium, e.g. NaCl (yellow); and an encapsulated (or not) sample (red).

Firstly, we are not aware of the synthesis of a binary nitride from the elements using any known large-volume HP-HT apparatus, in contrast to a LH-DAC. This is because cryogenic loading of (purified) *l*-N_2_ in the reaction volume of such an apparatus is a demanding procedure e.g [140,141]. If binary precursors with the needed N:M ratio are not available, the HP-HT nitrides with N:*M* > 1 can be synthesised via one of the following ways: (i) decomposition of a compound having higher N:M ratio than that in the intended compound, e.g. 2 Ta_3_N_5_ → 3η-Ta_2_N_3_ +0.5N_2_ [108] or nanocrystalline A_3_N_4+x_ (where x > 0) to c-A_3_N_4_ (where A = Zr or Hf), obtained via the high-temperature ammonolysis of the metal dialkylamide, i.e. A(NEt_2_)_4_ [88], (ii) decomposition of a compound containing only metal, nitrogen and hydrogen while the N:M ratio exceeded that in the intended product, e.g. 3 Si_2_N_2_(NH) → 2 γ-Si_3_N_4_ + NH_3_ [52], and (iii) metathesis reaction similar to that given by Equation (4) [64,83,128]. The paths (i) and (ii) permitted fabrication of completely or partially densified macroscopic bodies which sizes were sufficient for the reliable property measurements described below. However, porous samples (with porosity up to 30%, *p* ≤ 0.3) were rather usual for the here-considered nitrides. For this reason, less-common methods of property measurements not requiring well-densified samples were developed and presented below.

Even though the precursors were oxygen-free, the recovered HP-HT products are not necessarily as pure as the precursors. This is because main elements of the HP-assemblies surrounding the samples in large-volume apparatuses are oxides (MgO, LaCrO_3_, Al_2_O_3_, ZrO_2_ etc.) and a hydrous aluminium silicate, known as the mineral pyrophyllite, Al_2_Si_4_O_10_(OH)_2_ (Figure 9). Upon heating, oxygen from the hot regions of the HP-HT assembly was observed to diffuse into the sample volume through capsules made of noble or inert metals (e.g. Pt or Re). Depending on the capsule material, temperature and heating duration, oxygen contamination can be significant and distribution of the oxidised material throughout the volume not uniform (Figure 10A). In order to obtain nitride samples with a low degree of oxygen contamination (Figure 10B), several versions of diffusion barriers were proposed, including double or triple capsulation [52,76]. Furthermore, high porosity of the recovered products, up to *p* = 0.3, was typical for the recovered HP-HT nitrides (e.g Figure 10C). This can be explained by high hardness of the precursors and products but condensed nitrogen and hydrogen (if present), released upon decomposition of the over-stoichiometric precursors, could also have contributed to the porosity formation.

**Figure 10.**
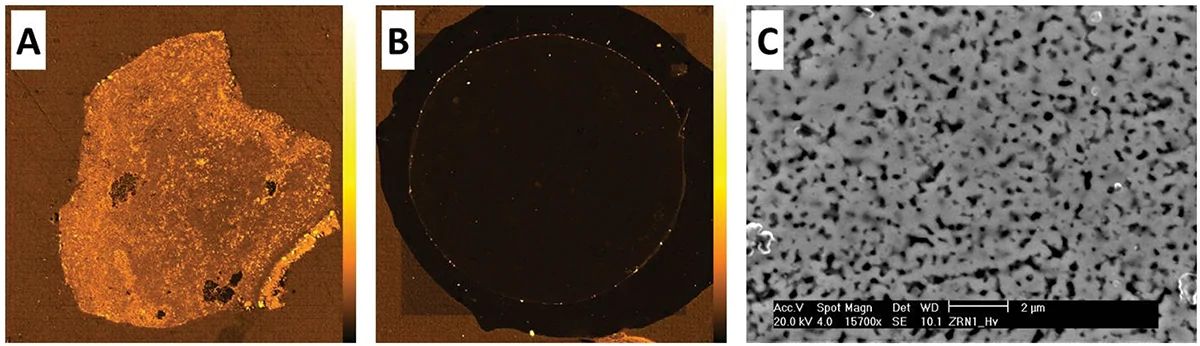
(A-B) Colour maps of oxygen distribution in two samples of γ-Si_3_N_4_ synthesised in a MAA [52,76]. Courtesy of Dr. Marcus Schwarz. (A) the sample of ~1.6 mm in diameter was enclosed in a single-wall Pt-capsule. The highest value of the colour scale corresponds to a complete sample oxidation. (B) The sample of ~2 mm in diameter was enclosed, during the HP-HT synthesis, in a triple-capsule with the innermost Pt-capsule, outermost h-BN capsule and molybdenum between them. In this sample, the highest oxygen content was measured to be < 4 wt.%. (C) SEM image of polished surface of a sample of oxygen-bearing c-Zr_3_N_4_ revealing high porosity, up to *p* = 0.3.

## 4. Mechanical properties

### 4.1. Elastic moduli

Measurement of complete tensor of single crystal elastic moduli at ambient conditions and, eventually, at high pressures and temperatures, *C*_ij_(*P,T*), would cover the theme of this section. This is because the knowledge of all *C*_ij_(*P,T*) is sufficient for the extraction of bulk and shear moduli (*B* and *G*, respectively) of isotropic polycrystalline bodies using well-known approaches [142–146]. However, growing of sufficiently big single crystals of novel compounds is laborious, especially at high pressures, and wouldn’t be honoured until their properties were proved to be of exceptional interest promising technological advancement. Accordingly, techniques and/or their combinations permitting access to the reliable values at reasonable costs using imperfect samples (e.g. porous polycrystalline bodies) are presented below. Except measurement of *B*(*P*), the presented here approaches are not common in the high-pressure research even though their reliability was confirmed.

Long-term progress in investigation of solids compressed in a DAC using XRD permits straightforward measurement of equation of state (EOS) of any crystalline compound, i.e. dependence of its density on pressure, ρ(*P*), with sufficiently high precision provided its crystal system is known e.g [147,148]. Important is that the extracted dependence ρ(*P*) corresponds to that of an ideally densified single-phase sample because the XRD method provides crystallographic density of the examined compound.

For nearly all of the above mentioned novel nitrides, bulk modulus, *B*_0_, and its first pressure derivative, *B*_0_′, were derived from fits of various empirical and semi-theoretical EOSs to the experimental ρ(*P*)-data points. However, the third-order Birch-Murnaghan EOS [149]

(8) $$P=\frac{3}{2}B_{0}\left({\left(\frac{\rho }{\rho_{0}}\right)}^{\frac{7}{3}}-{\left(\frac{\rho }{\rho_{0}}\right)}^{\frac{5}{3}}\right)\left(1-\frac{3}{4}\left(4-B_{0}^{\prime }\right)\left({\left(\frac{\rho }{\rho_{0}}\right)}^{\frac{2}{3}}-1\right)\right)$$

appears to be the most useful by extraction of properties of the HP-HT materials. The main advantage of this EOS is the possibility to easily recognise deficits of the collected ρ(*P*) data and of the selected fitting procedure when the data are represented in terms of normalised pressure, *F* = *P*/[3*f*(1 + 12*f*)^5/2^], versus effective strain, *f* = [(ρ/ρ_0_)^2/3^-1]/2 (see Figure 11 and discussion therein). The reported values of *B*_0_ and *B*_0_′ of the novel binary nitrides are summarised in Table 5. Reliability of the EOS-data depends on the following experimental details: (i) a sample should contain a sufficiently large number of grains within the volume irradiated by an X-ray beam thus excluding coarsening of the collected 2D-XRD patters (see examples in Figures 7 and 8A). Failure to do so can cause artificial displacement of peaks in the X-ray diffractograms obtained using a linear detector or after azimuthal integration of the collected 2D-XRD patterns and thus incorrect ρ(*P*) (see Supplemental Information to Ref. [126]). (ii) The sample should be imbedded in a quasi-hydrostatic pressure transmitting medium (PTM) and the latter should be as thick as possible. This minimises uncertainties due to pressure gradients if pressure is measured using a gauge (e.g. fluorescence of a ruby grain) located at some distance from the sample. This is because pressure gradients depend not only on yield strength, σ_y_(*P*), of the PTM but also on its thickness, *h*_PTM_(*P*), as follows from the simplified equation [160]:

(9) $$\frac{\mathrm{dP}}{\mathrm{dr}}=-\frac{\sigma_{y}\left(P\right)}{h_{\mathrm{PTM}}\left(P\right)}$$

**Figure 11.**
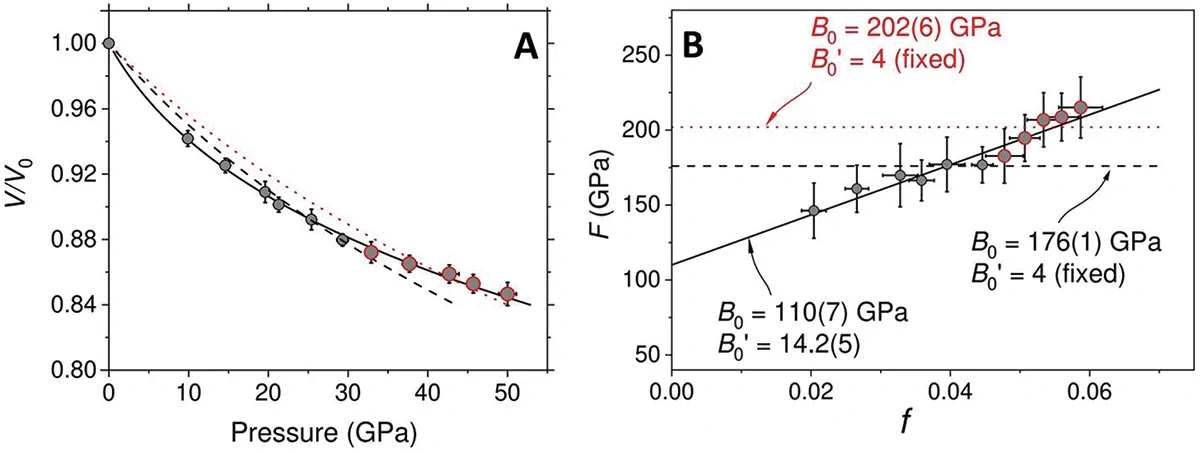
Visualisation of mistakes in *B*_0_ derived from (A) a synthetic *V*(*P*)/V_0_ data-points (grey circles) with a strong dependence of bulk modulus on pressure. (B) the same data-points represented in terms of *F* versus *f*. Fitting of the third order Birch-Murnaghan EOS (black solid line) provided *B*_0_ = 110(7) GPa and *B*_0_´=14.2(5). Assumption of *B*_0_´=4 (black dashed line) led to *B*_0_ = 176(1) GPa which is 60% higher than the previous value. Use of a part of the *V*(*P*)/V_0_ data-points at the high-pressure end (highlighted by red circles) and keeping *B*_0_′=4 resulted in *B*_0_ = 202(6) GPa which is 83% higher than *B*_0_ = 110(7) GPa.

**Table 5.** Experimental elastic moduli of polycrystalline high-pressure nitrides metastable at atmospheric pressure. A big majority of the shown *B*_0_ values were extracted from experimental ρ(*P*) while those extracted from the Birch-Murnaghan EOSs assuming fixed *B*_0_′=4 are not included if results for the third order Birch-Murnaghan EOS were provided. Data obtained or derived using results of other techniques such as NIT, BLS or psLU are included.

	*B*_0_ (GPa)	*B*_0_´	*G*_0_ (GPa)	References
γ-Si_3_N_4_	300 (10)^a^ 308 (5) 290 (5) 303 (4)^c^ 317 (16) 334 (7)	3.0 (1) 4.0 (2) 4.9 (6) n.a. 6.0 (8) 4.1 (9)	≥146(16)^b^ ≥145(15)^b^ ≥148(16)^b^ 248(1)^c^ ≥143(17)^b^ ≥141(14)^b^	[150] [54] [31] [35] [70] [151]
γ-Ge_3_N_4_	295 (5) 254 (13) 322 (44)^e^	3.8 (2) 6.0 (7) n.a.	≥124(17)^d^ ≥130(20)^d^ 188(7)^e^	[50] [70] [45]
γ-Sn_3_N_4_	149 (1) 191.8 (5) 209 (1)^g^ 158 (11)	4 - fixed 3.97 (3) 4 – fixed 5.4 (1.1)	≥68(23)^f^ ≥63(20)^f^ ≥62(20)^f^ ≥67(22)^f^	[65] [152] [153] [29]
c-Zr_3_N_4_	219 (13) 217 (20)^e^ 213 (1)	4.4 (1.0) n.a. 4 - fixed	≥96(13)^h^ 163(9)^e^ >97(11)^h^	[154] [155] [83]
c-Hf_3_N_4_	227 (7)	5.7 (6)	n.a.	[156]
η-Ta_2_N_3_	319 (1) 311 (6) 281 (15)^e^	4 – fixed 4.67-fixed n.a.	124–139^i^ 124–141^i^ 123(2)^e^ 129–144^j^	[110] [110] [157] [158]

^a^isentropic shock compression.

^b^calculated using Equations 13 and 14 and *E*_r0_ ≥ 303(17) GPa from NIT of a polycrystalline sample synthesised at HP-HT conditions in a MAA [31].

^c^BLS measurements on a transparent polycrystalline sample densified at HP-HT conditions in a MAA.

^d^calculated using Equations 13 and 14 and *E*_r0_ ≥ 275(21) GPa from NIT of a polycrystalline sample synthesised at HP-HT conditions in a MAA, see [34,159].

^e^psLU measurements where the sample porosity was taken into account.

^f^calculated using Equations 13 and 14 and *E*_r0_ ≥ 167(36) GPa from NIT of a polycrystalline sample preliminary compressed and heated in a MAA [34].

^g^the value could have been biased due to uncertainties in chemical composition of the sample and experimental data (see Supplemental Material to Ref [29].).

^h^calculated here using Equations 13 and 14 and *E*_r0_ ≥ 224(16) GPa from NIT of a polycrystalline sample synthesised at HP-HT conditions in a MAA [154].

^i^derived using *E*_r,p_ between 222 GPa and 238 GPa from NIT [158] where the contribution of porosity was taken into account.

^j^obtained from NIT data using *B*_0_ = 281(15) GPa [157] and taking porosity contribution into account.

This equation indicates that even usage a soft PTM, such as helium, does not guaranty correct *B*_0_ and *B*_0_′ because this solidified noble gas is typically loaded in gas-loaders at pressures around 0.12 GPa. Due to a high compressibility of helium at the low pressures, the sample volume becomes very thin when compressed to several GPa and strong lateral pressure gradients appear upon further compression even though σ_y_ of helium is smaller than that of the less-hydrostatic PTMs such as NaCl or solid argon [126,161,162].

In contrast to bulk modulus, a similarly simple and straightforward approach to measure *G*_0_ (not to mention the pressure dependence *G*(*P*)) not sensitive to the sample state (e. g. powder, porous body, admixture of another contaminating solid) is not available. However, several methods permitting measurement of the material property, involving bulk modulus, exist. For this reason, an experimental uncertainty in bulk modulus should be as small as possible because it contributes to that of the derived shear modulus. Examples of such classical techniques are the resonance ultrasonic spectroscopy and Brillouin light scattering (BLS) [163–167] who typically provide only longitudinal sound velocities, *V*_L_, given by the equation

(10) $$V_{L}=\sqrt{\frac{3B+4G}{3\rho }}$$

which requires knowledge of the sample density, ρ. However, only single crystals or highly densified polycrystalline samples are suitable for these techniques e.g [35]. Unfortunately, such samples are rarely available for novel materials including the HP-HT nitrides considered in this paper.

For this reason, two advanced techniques, less known in the high-pressure domain, were adapted and further developed, namely picosecond laser ultrasonics (psLU) and nanoinentation testing (NIT). It was demonstrated that they permit extraction of reliable *B*_0_ and *G*_0_ using polycrystalline bodies having high porosities, up to *p* = 0.3.

The psLU technique permits measurement of velocities of surface acoustic waves (SAWs) for samples with lateral dimensions below 500 μm (Figure 12) and, thus, is well suitable for those synthesised in large volume HP-HT apparatuses. It is a relatively simple all optical non-contact and non-destructive approach developed more than 50 years ago e.g [168]. Detailed theoretical and technical descriptions of the technique including mechanisms of the SAW generation, efficiency of the energy transfer, shape of the surface perturbation, propagation and detection of SAWs (Figure 12B) can be found elsewhere e.g [169,170]. The main conditions imposed on a sample are lateral sizes of at least 150 μm, optical quality of its surface and sufficiently strong absorption of the pump-laser radiation used to excite the SAWs and reflection of the probe-laser beam used to detect their arrivals. The latter conditions are fulfilled for metals, such as η-Ta_2_N_3_ [157,171] and low band-gap semiconductors such as c-Zr_3_N_4_ [155]. The technique did also work for a large band-gap semiconductor, γ-Ge_3_N_4_, due to the presence of impurities or the presently-not-identified optical-absorption centres [45]. Interestingly, the technique provided sufficiently strong signals even for samples with porosities approaching *p* = 0.3. For a transparent sample, a thin metallic film should be deposited on the polished surface to ensure absorption of the pump-laser radiation and reflection of the probe-laser beam.

**Figure 12.**
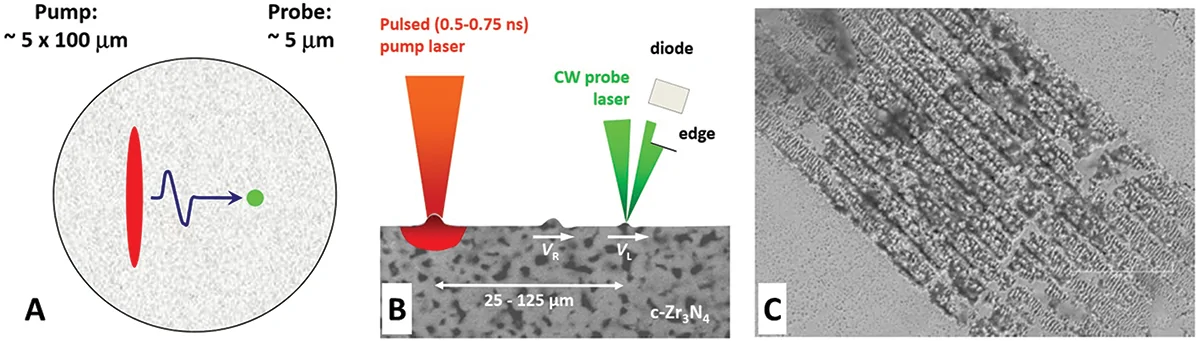
Principals of the psLU method of the SAW velocity measurement. (A) schematic of geometry of spots of a pump- (red) and probe (green) laser beams used to excite the SAW pulses and detect their arrivals, respectively. (B) schematic of propagation of the SAW pulses on surface of a porous sample of c-Zr_3_N_4_. (C) SEM image of traces of the material ablation when energy of the pump-laser pulses exceeded the threshold value. Distances between the traces of ablation caused by the pump-laser beam are identical and equal to 8.3 μm.

In order to excite SAW-pulses on polished surface of a sample, pulsed radiation of a laser (e.g. Nd:YAG-laser, λ = 1064 nm, pulse duration 0.5–0.75 ns, Teem Photonics, France) focused to a circular spot of several micrometres in diameter by an objective (for example NACHET (France), magnification × 20), can be used. The SAW-pulses propagate then radially and the related surface perturbations can be used to detect their arrivals at a particular distance from the pump-laser spot (Figure 12B). If duration of propagation of the detectable SAW-pulses, Δ*t*, due to the surface Rayleigh waves (R) or skimming longitudinal waves (L) is known then the corresponding sound velocities, *V*_R_ or *V*_L_, can be extracted. However, in the above described geometry the SAW-pulses decay rapidly with distance to the pump-laser spot. Because the laser-pulse energy cannot be increased infinitely due to the material ablation (Figure 12C), mounting of a cylindrical lens in front of the objective transforms the pump-laser radiation to a line (with dimensions 5 μm × 100 μm, for example) which permits stronger pump-laser pulses and, accordingly, amplification of the SAW amplitudes (Figure 12A). As a result, the SAW-pulse arrivals can be detected at much longer distances from the pump-laser line which is especially important for quickly fading longitudinal waves (Figure 13A). In the above mentioned works, the knife-edge method was used to detect arrivals of the SAW-pulses as schematically shown in Figure 12B. Changing the distance between the pump- and probe laser spots and measuring the arrival times, *t*, for each type of the SAW-pulses is sufficient to extract their velocities, *V*_R_ and/or *V*_L_ (Figure 13B). Further methods of detection of the SAW-pulse arrivals are described elsewhere [170].

**Figure 13.**
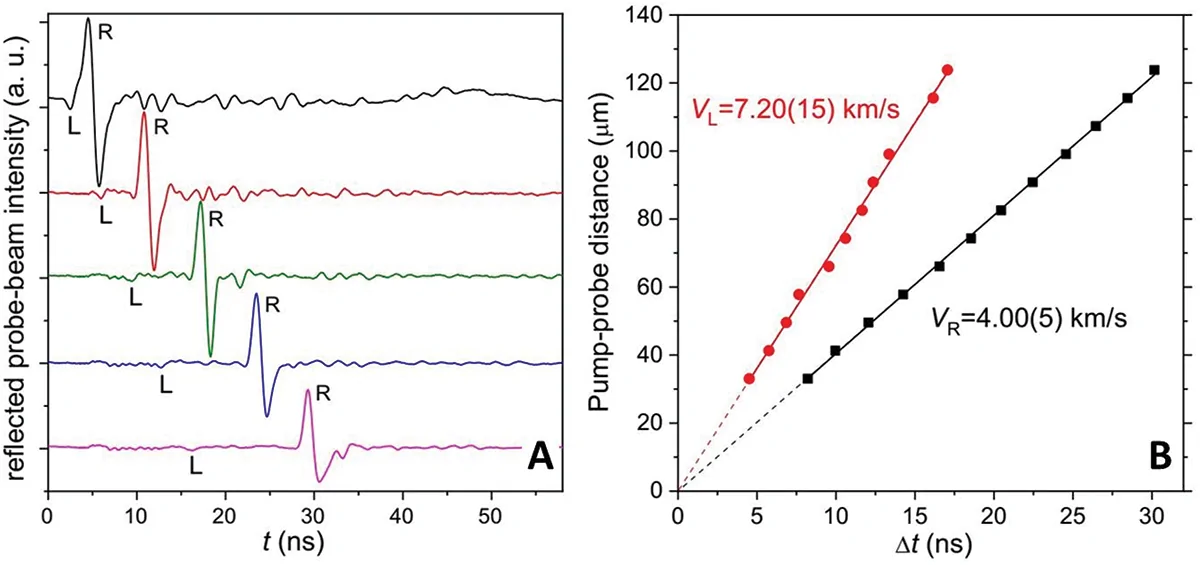
(A) examples of photoacoustic signals collected from the surface of a porous sample of the oxygen-bearing c-Zr_3_N_4_ [155]. They were recorded at the pump-probe distances from 24.9 μm to 123.9 μm in steps of 8.3(5) μm (only each third record is shown). Signals due to arrivals of the surface Rayleigh waves (R) are much stronger that those due to the longitudinal waves (L). (B) distance between the pump- and probe laser spots as a function of propagation time, Δ*t*, of each type of the SAW-pulses (results at the distance below 24.9 μm were not considered due to a strong overlapping of the two pulse types). Solid lines represent results of the linear least-square fits to the experimental data which slopes provide the sound velocities, *V*_R_ and *V*_L_. Dashed lines are extensions of the linear fits confirming the same origin of both types of the SAW-pulses.

If *V*_L_ and *V*_R_ are known then elastic moduli of the examined material can be extracted using Equation 10 combined with the following well-known equations:

(11a) $$V_{T}=\sqrt{\frac{G}{\rho }}$$

(11b) $${\left(V_{T}^{2}-\frac{V_{R}^{2}}{2}\right)}^{2}=\frac{V_{T}^{3}}{V_{L}}\sqrt{V_{L}^{2}-V_{R}^{2}}\sqrt{V_{T}^{2}-V_{R}^{2}}$$

where *V*_T_ is velocity of the transversal acoustic waves.

As already mentioned above, the psLU technique permits detection of both types of SAW-pulses for porous polycrystalline samples and, accordingly, determining of the sound velocities, *V*_R.p_ and *V*_L,p_. The elastic moduli, *B*_p_ and *G*_p_, can be extracted if the sample porosity, *p*, is known for the calculation of the density ρ_p_ = ρ_0_*p*. Deduction of the elastic moduli, *B*_0_ and *G*_0_, requires evaluation of the porosity influence on the elastic response under load. In general, this is a complex problem because (i) it requires solving a boundary-value problem taking into account spatial fluctuations of the elastic properties and (ii) microstructure of a porous material exhibits random character precluding a comprehensive description. However, when the average pore size is much smaller than the sample and the acoustic wavelength, λ_a_, macroscopic elastic properties can be estimated using homogenisation approaches, and verified by comparison with the *B*_0_ obtained independently from an experimental EOS. Hashin–Shtrikman approach [145] has been used to estimate the effective elastic behaviour of the examined porous nitrides by taking into account the pores shape and spatial distribution jointly through the pores autocorrelation function (PAF). The latter parameter provides information on the pores spatial distribution, shape and characteristic length scale [172]. The PAFs can be extracted from SEM images via fast Fourier transform algorithms thanks to the Wiener–Khinchin theorem and/or by numerical analysis (Figure 14).

**Figure 14.**
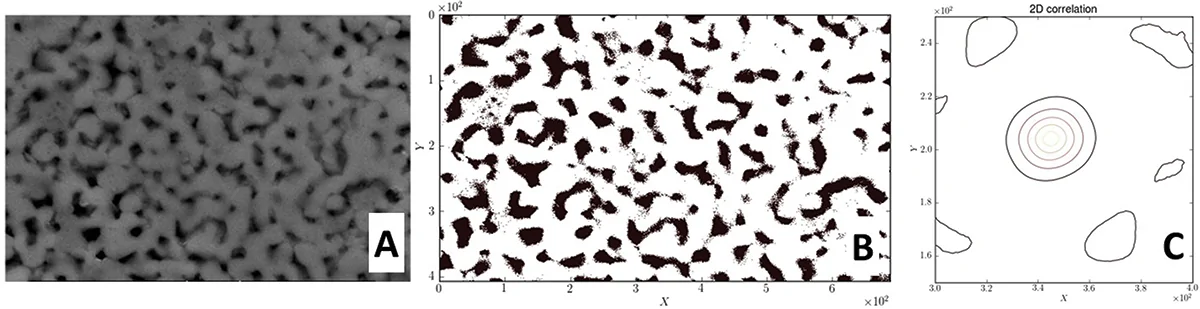
Extraction of a PAF from an SEM image: (A) SEM image of polished surface of a porous polycrystalline sample of oxygen-bearing c-Zr_3_N_4_. (B) black-white representation of the image shown in (A) providing visualisation of pores in the sample. The scales show pixel numbers. The image in (B) was used to derive a projection of the PAF showing only a weak deviation from isotropy (C).

If PAFs obtained from several SEM images for two or more nonparallel surfaces show only weak deviation from the isotropy and no preferential orientation can be recognised for the sample cross-sections than a spherical shape of the PAF can be used to compute *B*_p_ and *G*_p_ using Hashin–Shtrickman approach:

(12a) $$B_{p}=\frac{\left(1-p\right)B_{0}}{1+p\left(3B_{0}/4G_{0}\right)}$$

(12b) $$G_{p}=\frac{\left(1-p\right)G_{0}}{1+p\left(8-10\nu_{0}\right)/\left(7-5\nu_{0}\right)}$$

Apparently, the same equation can be used to deduce *B*_0_ and *G*_0_ if *B*_p_, *G*_p_ and *p* are known. Agreement of the *B*_0_ = 217(20) GPa, obtained for the oxygen-bearing c-Zr_3_N_4_ from such psLU measurements on a porous polycrystalline sample, with *B*_0_ = 219 (13) GPa from the EOS measurements (Table 5) confirms reliability of the psLU approach. The agreement is also reasonable for γ-Ge_3_N_4_ if *B*_0_ = 322(44) GPa from the psLU measurements [45] is compared with *B*_0_ = 295(5) GPa derived from the EOS obtained using argon as a soft PTM [50]. In the case of η-Ta_2_N_3_, the psLU-measurements provided somewhat lower *B*_0_ = 281(15) GPa [157] when compared with *B*_0_ = 319(1) GPa obtained from the experimental ρ(*P*) under assumption of fixed *B*_0_´ = 4.0 [110]. Interestingly, the difference is smaller if we compare with *B*_0_ = 311(6) GPa obtained for the same ρ(*P*) data but with *B*_0_´ fixed at 4.67 predicted in the DFT calculations [110]. In summary, the psLU technique permits extraction of reliable *B*_0_- and *G*_0_ values for ideally densified samples even though the measurements were performed on porous ones.

Another method of determining shear modulus is suitable for any solid samples (conducting or not) including polycrystalline completely or partially densified ones obtained in large-volume HP-HT apparatuses. However, the method requires knowledge of *B*_0_ from an experimental EOS, for example. In this approach, reduced elastic modulus, *E*_r_, is first determined via nano-indentation testing (NIT) (Figure 15):

(13) $$\frac{1}{E_{r}}=\frac{1-\nu^{2}}{E}+\frac{1-\nu_{d}^{2}}{E_{d}}$$

**Figure 15.**
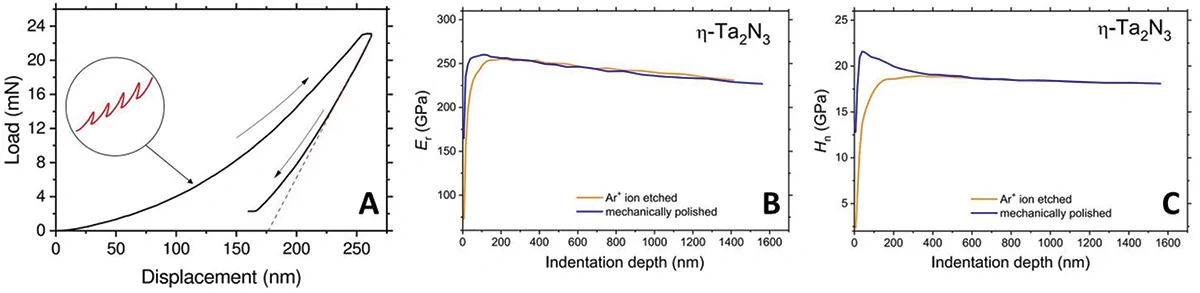
(A) illustration of two types of nano-indentation testing (NIT) where black solid line shows a load-unload curve: in the Oliver-Pharr method, the Berkovich pyramid is continuously pressed in the sample and then unloaded while the applied load and displacement are measured. The first third of the unloading curve is then used to determine *E*_r_ and interception of its linear extrapolation with the abscissa provides nano-indentation hardness, *H*_n_. In the CSM method, the loading-unloading cycles are performed in small steps while the total load is successively increased (see insert) and *E*_r_ and *H*_n_ are recovered at each step as functions of the indentation depth. The CSM method permits revealing of property changes with depth as was the case for *E*_r_ and *H*_n_ of η-Ta_2_N_3_: blue lines show change of *E*_r_ (panel B) and *H*_n_ (panel C) with indentation depth for mechanically polished surface of a porous sample of η-Ta_2_N_3_. Orange lines show *E*_r_ and *H*_n_ values for the same sample after the surface etching by the Ar^+^ ion beam which removed the strongly densified material at shallow depths, up to ~400 nm, formed during the polishing.

where *E* is Young’s modulus and ν Poisson’s ratio of the examined material, *E*_d_ = 1141 GPa and ν_d_ = 0.07 are the values for diamond, material of the Berkovich pyramid used as the indenter. As soon as *E*_r_ is measured, shear modulus can be recovered using the well-known equations:

(14a) $$E=\frac{9\mathrm{BG}}{3B+G}$$

(14b) $$\nu =\frac{1}{2}\frac{3B-2G}{3B+G}$$

In case of a porous sample, the contribution of porosity to Young’s modulus and Poisson’s ratio can be evaluated using the above described approach based on the PAF shape and Equations 12. Unfortunately, in a big majority of examinations of novel nitrides using NIT, porosity was not examined and, accordingly, its contribution to the samples’ elastic response ignored. The only exception is the work of Bourguille et al. on η-Ta_2_N_3_ [158] where two indentation methods were used, the classical Oliver-Pharr (OP) and the more recent continuous stiffness mode (CSM) (Figure 15). Depth resolution of the CSM method permitted not only determining of *E*_r_ and of nano-indentation hardness, *H*_*n*_, of a porous polycrystalline sample of η-Ta_2_N_3_ but also revealed densification of the upper sample layer caused by mechanical polishing (Figure 15B,C). As can be seen in Table 5, shear modulus extracted using the OP indentation method, *G*_0_ = 129(22) GPa, agrees with *G*_0_ = 123(2) GPa obtained from the independent psLU-measurements on the same sample. The agreement indicates reliability of the here-considered approaches of determining *G*_0_ of novel nitrides applying the NIT- and/or psLU techniques to porous samples from the HP-HT synthesis in large-volume apparatuses.

Finally, the recently adopted technique of time-domain Brillouin scattering permits determining single crystal elastic moduli, *C*_ij_, of a cubic solid using a transparent polycrystalline sample provided *B*_0_ is known. A detailed description of this technique and modus operandi was presented in several recent publications [161,173,174]. Moreover, the technique permits 3D-visualisation of texture and phase transitions taking place in the sample interior [175–177].

### 4.2. Hardness and other inelastic properties

Traditionally, the search for novel HP-HT materials was motivated by the hope to discover a novel compound exhibiting hardness superior to that of diamond or, at least, of cubic BN. Other properties might also be of interest but are not specifically pursued even though a combination of not exceptional but advanced properties could open new perspectives. Such advanced properties could be stability in air at elevated temperatures or in tribological contact with a particular class of materials, advantageous thermal-shock- or fracture resistance or resistance against irradiation with energetic particles such as neutrons from a thermonuclear reactor. It was also proposed that some of the above discussed HP-HT nitrides could be used as anodes in batteries [62,67] or for sensing of ethanol vapour [66]. Apparently, if a compound is evaluated as interesting, it should be available on industrial scale at reasonable costs or, at least, available as macroscopic objects independent of costs if the advancement in functionality is exceptional. It should be mentioned that potential methods of the industrial production of novel nitrides are not limited by HP-HT processes but include various approaches of deposition of films and coatings.

With respect to mechanical properties, two novel nitrides, namely γ-Si_3_N_4_ and c-Zr_3_N_4_, appear to be industrially valuable. Polycrystalline γ-Si_3_N_4_ was reported to exhibit high Vickers microhardness, similar or exceeding *H*_V_ = 35 GPa and thus to compete with a few traditional compounds for the rank of the third hardest solid (Table 6). Its indentation fracture toughness *K*_Ic-if_ = 3.2–3.8 MPam^1/2^ [35,76] is comparable with the certified value for β-Si_3_N_4_ of *K*_Ic-if_ = 4.6(2) MPam^1/2^ [76], the well-known tough ceramic material. However, *K*_Ic_ values up to 8.2 MPam^1/2^ were also reported for β-Si_3_N_4_, depending on the microstructure and volume fraction of the needle-like grains of this hexagonal low-pressure phase [209]. Furthermore, γ-Si_3_N_4_ persists heating in air to 1773 K [55] and was estimated to exhibit thermal shock resistance insignificantly below that of β-Si_3_N_4_ (also known as an exceptional thermal-shock resistant ceramics) [29]. This property has been established at a relatively early stage of exploration of γ-Si_3_N_4_ (and of γ-Sn_3_N_4_) due to a recently proposed approach requiring data from standard high-pressure measurements. In particular, thermal-shock resistance was shown to be proportional to the ratio of hardness to Grüneisen parameter, *H*_V_/γ, where γ can be derived from pressure dependences of the Raman band frequencies and bulk modulus of the examined compound [29]. Interestingly, none of the needed values require exact knowledge of composition and/or structure of the sample material, just its crystal symmetry. Finally, samples of γ-Si_3_N_4_ transparent in a broad range of light (see below) with sizes up to 2 mm in diameter are available [35,203]. All these properties make γ-Si_3_N_4_ suitable for fabrication of passive optical elements (windows or lenses) in front of (opto)electronics/detectors used under a wide range of extreme thermal and/or mechanical attacks.

**Table 6.** Author’s selection of the most reliable values of Vickers microhardness, *H*_V_, of several (super)hard solids including those which can be obtained as macroscopic objects of ~1 mm in size via the HP-HT synthesis or as thin films. Only reports indicating applied loads (shown between / … /) were considered. Hardness values (instead of microhardness) obtained with high loads and, thus, almost independent of it are marked as /H/. Temperatures prior to decomposition, conversion to a low-pressure phase or oxidation (ox.) are included.

Compound	*H*_V_ (GPa) /load in N/	References	thermal (oxidation) stability, K	References
Diamond	78–99/H/	[178]	800–1200 (ox.)	[179–181]
			900	[182]
c-BN	49/H/	[183]	973–1623 (ox.) 1473–1673	[184] [178]
B_4_C	36/H/	[185]	<873 (ox.)	[186]
α-SiC	27/5/	[187]	1773 (ox.)	[188]
β-SiC (3C)	23/2/	[189]	1773 (ox.)	[188]
WC	17–21/H/	[190]	873 (ox.)	[191]
α-Si_3_N_4_	31/3/	[192]	1373 (ox.)	[193]
β-Si_3_N_4_	15^a^/H/ 20/5/	[194] [195]	1373 (ox.) 1673 (ox.)	[193] [196]
δ-TiN	17/3/	[197]	1073	[197]
δ-ZrN	<13/H/	[198]	1073–1273	[199]
Al_2_O_3_	<19/H/ 18/10/	[200] [201]	2345^b^	[202]
γ-Si_3_N_4_	35/10/ 37/10/	[35] [203]	1773 (ox.) 1673 (ox.)	[55] [30]
γ-Ge_3_N_4_	28/1/ 25/5/	[34] [204]	<1000	[61]
γ-Sn_3_N_4_	11/1/	[34]	673	[68]
c-Zr_3_N_4_	>12^c^/10/ ~37^d^/0.005/	[82] [205]	<773	[82]
c-Hf_3_N_4_	>11^c^/10/	[206]	≥473	[207]
η-Ta_2_N_3_	>16^c^/10/	[208]	n.a.	

^a^with a densification aid; ^b^melting; ^c^porous sample; ^d^dense film with thickness of 0.78 μm.

Furthermore, optical elements made of γ-Si_3_N_4_ could be considered for application in nuclear fusion because it is expected to exhibit significant radiation resistance [210]. Such optical materials could be substantial elements in various diagnostic systems of future deuterium-tritium fusion reactors, which have to withstand 14 MeV neutron irradiation of unprecedented intensity. Radiation-induced lattice defects strongly affect functionality of optical components (windows, lenses, fibres, LEDs, etc.) and therefore understanding of the defects creation and annealing processes in solids is of fundamental importance. It was recognised that ternary oxides of the type TQ_2_O_4_ having spinel structure possess an increased neutron tolerance [211,212] due to an efficient self-healing interstitial-vacancy (*i-v*) recombination process, the prerequisite of which is the presence of a huge density of so-called structural vacancies in the lattice: metal cations T and Q occupy only one-eighth of the available tetrahedral sites and one-half of the available octahedral sites, respectively, so that the overall cation to anion ratio is 3:4. In solids with spinel structure, an additional efficient mechanism of accommodation of radiation induced defects exists, namely swapping of positions of cations between tetrahedral and octahedral sites (cation antisite defect formation). It has been shown in several papers e.g [213]. that the energy costs for antisites formation in MgAl_2_O_4_ is much lower than for all other elementary lattice defects, which means that the cation sublattice easily accommodates disorder or, in other words, is radiation tolerant. The so-called site preference energy for cations has been introduced for analysis of enthalpy increase due to such cations swapping in the spinel lattice, and it is not zero if the cations T and Q are of different type [214]. Accordingly, higher site preference energy signifies the higher stability of the ordered cation sublattice. The latter has already some experimental verification e.g [215]. where higher enthalpy increase due to the cation swapping, e.g. caused by radiation, occurs. That is why one can expect a higher radiation tolerance of solids with spinel structure which have only one type of cations because in this case swapping the positions of cations does not change anything in the lattice. Except for the rare Fe_3_O_4_ and a special case of the less-useful nanocrystalline γ-Al_2_O_3_, oxygen based solids with spinel structure contain two different types of cations. This is why the high-pressure nitrides of the group 14 elements, and especially γ-Si_3_N_4_, should be considered as an alternative solution by the search for radiation tolerant materials representing a new class of single cation solids with spinel structure.

Suitability of c-Zr_3_N_4_ for industrial applications as an advanced wear-resistant coating became evident after a report on successful deposition of this nitride as thin films/coatings on various substrates and testing of its wear resistance [205]. In particular, films of c-Zr_3_N_4_ were deposited on silicon, NaCl, quartz, and cemented carbide. The films were deposited using only slightly modified version of a broadly used in the industry technique of physical vapour-deposition, namely filtered cathodic arc (FCA) method. Its standard version is used to deposit well-known coatings of mononitrides of the group 4 and 5 elements (e.g. δ-TiN) and related compounds (e.g. δ-Ti_1-x_Al_x_N) on cutting tools with the aim to improve their wear resistance in general and by cutting of steels in particular: For example, coating of a tool made of cemented carbide with δ-TaN improves its wear resistance by milling of steels by ~2 times and coating with δ-Ti_1-x_Al_x_N by 3–5 times, depending on the way of the wear measurement (Figure 16). In contrast, coating of a hard-metal tool with c-Zr_3_N_4_ leads to the wear reduction by ~10 times when compared with δ-TiN [205], and by ~5 times, if compared with the best δ-Ti_1-x_Al_x_N coating considered in Figure 16. Thus, application of coatings based on c-Zr_3_N_4_ promises significant advancement in productivity and efficiency in the metal-working industry not to mention advantages due to reduced consumption of costly tungsten, energy, investments in occupational health (due to the presence of cobalt [131]) and mitigating of environmental impact associated with fabrication of novel and recycling of discarded tools based on cemented carbide.

**Figure 16.**
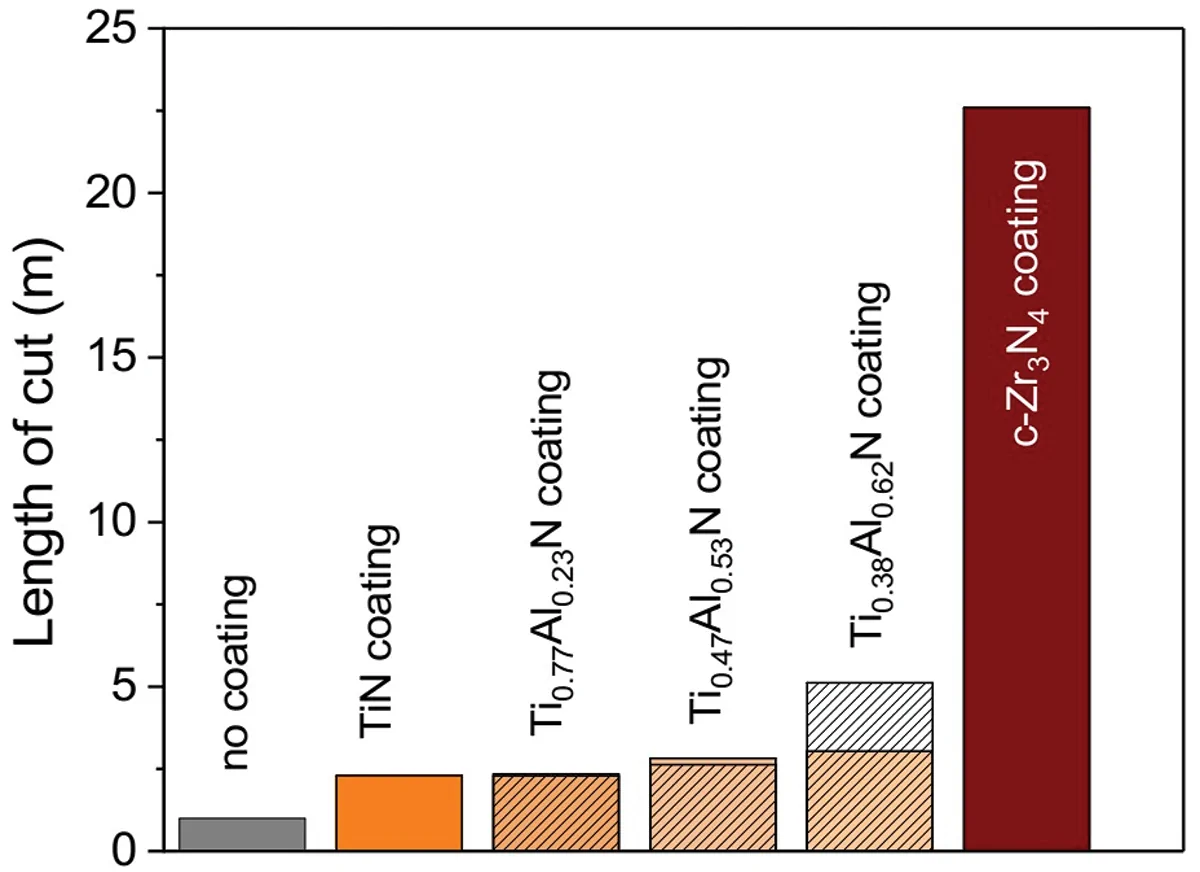
Wear resistance of a selection of compounds used as coatings on cutting tools made of cemented carbide. Here, wear resistance is expressed in terms of lenth of cut (*L*_cut_) needed to achieve the same width of worn flank and is normalised with respect to that of a noncoated cemented-carbide tool, e.g. *L*_cut_ = 1 m (grey box). The *L*_cut_ values for the tools without coating, coated with δ-TiN (orange box) and c-Zr_3_N_4_ (dark-red box) are exctracted from ref [205]. The *L*_cut_ values for the three δ-Ti_1-x_Al_x_N coatings (with ×=0.23, 0.53 and 0.62) are extracted from ref [216]. where examination of wear of a δ-TiN coating was also presented. In order to compare the data reported for c-Zr_3_N_4_, wear resistance of the δ-TiN coatings was assumed to be equal in both works. In the work of Moreno et al. [216], wear resistance was quantified via analysis of the worn areas because the coating removal was not uniform while Chhowalla and unalan measured width of worn part of the tool flank [205]. For this reason, the results for the δ-Ti_1-x_Al_x_N coatings (normalised with respect to δ-TiN and then to uncoated cemented carbide) are presented in two ways: (1) light-orange boxes show *L*_cut_ obtained from widths of the flank regions where the non-uniformly worn areas were recognised after turning with the cutting speed of 80 m/min (Figure 4 in ref [216].). (2) hatched ‘transparent’ boxes show *L*_cut_ values obtained from normalised ratios of worn areas observed after turning with the same cutting speed, i.e. from the values given in Figure 5 of ref [216].

It should be mentioned here that not only hardness and wear resistance define performance of cutting tools but also fracture toughness, the property describing ability of a material to prevent failure of the coating and withstand mechanical and/or thermal stresses over time. This property is crucial by high-speed- or interrupted cutting, especially by machining of hardened metals. Indentation fracture toughness, *K*_Ic-if_, of a porous polycrystalline sample of η-Ta_2_N_3_, found to exhibit metallic conductivity [171], was measured to be 4.6(2) MPa⋅m^1/2^ [208]. This value is similar to that of sintered monoliths of such well-known fracture-resistant materials as β-Si_3_N_4_, β-SiC and their composites considered as promising tough ceramics: Polycrystalline bodies of hexagonal Si_3_N_4_ sintered using several rear-earth oxides RE_2_O_3_ (RE = La, Nd, Y, Yb, Lu) were reported to exhibit *K*_Ic-if_ = 5.2–7.1 MPa⋅m^1/2^ but addition of 5 vol.% of SiC led to *K*_Ic-_if = 4.3–5.4 MPa⋅m^1/2^ [217]. Hot-pressed Si_3_N_4_, sintered with 5 wt.% of Y_2_O_3_, showed *K*_Ic-if_ = 5.2 MPa⋅m^1/2^ while addition of 8 wt.% of SiC resulted an increase to *K*_Ic-if_ = 5.4–5.8 MPa⋅m^1/2^ [218]. For hot-pressed β-SiC with 9 wt.% of Y_2_O_3_ and Al_2_O_3_, *K*_Ic-if_ between 2.9 and 4.5 MPa⋅m^1/2^ was measured [219]. Because pores-free monoliths and coatings are expected to exhibit higher fracture toughness, *K*_Ic-if_ of densified η-Ta_2_N_3_ could surpass the above values for sintered β-Si_3_N_4_ and β-SiC. This conclusion was drawn from NITs on samples of polycrystalline η-Ta_2_N_3_ having mechanically densified surface layers where contribution of the dense material to the total sample response was significant (Figure 15). In particular, *K*_Ic-if_ was found to be 10–20% higher than for the same sample after removing the densified top layer [158]. Also, hardness of densified η-Ta_2_N_3_ was estimated, after the analysis of porosity, to be similar to that of the densified c-Zr_3_N_4_. Accordingly, all the necessary requirements for a wear-resistant material, except one, are fulfilled. The remaining step in the evaluation of suitability of η-Ta_2_N_3_ for machining of hardened steels and other alloys would be measurement of its wear resistance using a densified bulk sample or coating on a cemented-carbide- or ceramic tool. It is expected that chemical wear will not affect performance of such cutting tools because coatings of tantalum mononitride are already used for machining of high-strength alloys, titanium, stainless steel, and other hard-to-machine materials.

## 5. HP-HT nitrides as energy materials

Soon after their discovery in 1999, multiple calculations of electronic structure of nitrides of the group 14 elements with spinel structure, γ-M_3_N_4_, were published and interesting electronic properties predicted. In one of the earliest first-principals calculations, γ-Si_3_N_4_ was predicted to exhibit direct band gap, *E*_g_, equal or above 3.45 eV, a small effective mass of charge carriers at the conduction band but a very flat top of the valence band and, accordingly, large effective mass [220]. In that work, γ-Si_3_N_4_ was proposed to be suitable as basic material for blue or ultraviolet lasers or light emitting diodes (LEDs) due to its favourable band gap. However, a competing group predicted an indirect and larger band gap of *E*_g_ = 3.68 eV and effective mass of the *p*-type carriers to be similar to that of the free electron along at least one of the crystallographic directions in its single crystal [221]. Then, DFT calculations of electronic structures were extended to γ-Ge_3_N_4_ and γ-Sn_3_N_4_ and direct band gaps of 2.22 eV and 1.3–1.4 eV, respectively, and small effective masses of the *n*-type carriers predicted [222–224]. Later theoretical works confirmed that band gaps of the spinel-nitrides, γ-M_3_N_4_, are direct but their sizes depend (i) on the applied calculation method and (ii) on the lattice parameter selected in the calculations even when one and the same method is used [225,226]: For example, *E*_g_ of γ-Sn_3_N_4_ was predicted to be 0.58 eV, 1.53 eV and 1.42 eV using the DFT-LDA method, G_0_W_0_ with the plasmon-pole approximation (PPA) and G_0_W_0_ with the numerical integration (NI) method, respectively [225]. An even stronger difference in the calculated *E*_g_ of γ-Sn_3_N_4_, between 0.54 eV and 2.19 eV, were demonstrated in a recent theoretical work [226]. Increase of the cubic lattice parameter of γ-Sn_3_N_4_ from 97% to 103% of the optimal value (corresponds to the ‘uncertainty’ of ±3%) led to a decrease of *E*_g_ from 1.45 eV to 0 eV [225]. By using the G_0_W_0_ approach, *E*_g_ of γ-Sn_3_N_4_ also decreases with increasing unit cell size but the compound remains a semiconductor [225]. The most suitable elements for the *n*- and *p*-type doping were proposed both for γ-Si_3_N_4_ and γ-Ge_3_N_4_: phosphorus and oxygen were suggested as *n*-type dopants and aluminium as a *p*-type dopant for γ-Si_3_N_4_, while antimony and oxygen as *n*-type dopants and, again, aluminium as a *p*-type dopant for γ-Ge_3_N_4_ [227,228]. Also, stability and electronic structures of other imaginable binary and ternary compounds of the type γ-TQ_2_N_4_, including elements of the groups 4 and 14 were considered. It was predicted that among the ternary nitrides those with smaller cations in tetrahedral sites and larger cations in octahedral sites of the spinel structure should be stable [222]. However, in another theoretical work Ge atoms were stated to have a strong preference to reside at the tetrahedral sites [229]. More important outcome of this work was, however, the prediction that solid solution γ-(Si_x_Ge_1-x_)_3_N_4_ could form at temperatures above ~1400 K and, apparently, high pressures.

The first attempts to measure band gap of a spinel nitride were devoted to γ-Si_3_N_4_, soon after its discovery, but precise values and experimental conformation of a direct band gap were provided 17 years later (see below). Interpretation of the data obtained for a powder sample cold-compacted by squeezing between anvils of a DAC to the thickness of ~30 μm using optical absorption and cathodoluminescence (CL) spectra led to an underestimated *E*_g_ = 3.25 eV [230]. In the case of optical absorption spectra (Figure 17A), it was assumed that the band gap is direct and photon-energy dependence of the optical density, **OD**(*E*), can be described as

(15) $$-\mathrm{lg}\left(\frac{I\left(E\right)}{I_{0}\left(E\right)}\right)=\mathrm{OD}\left(E\right)=\frac{\sqrt{E-E_{g}}}{E}$$

**Figure 17.**
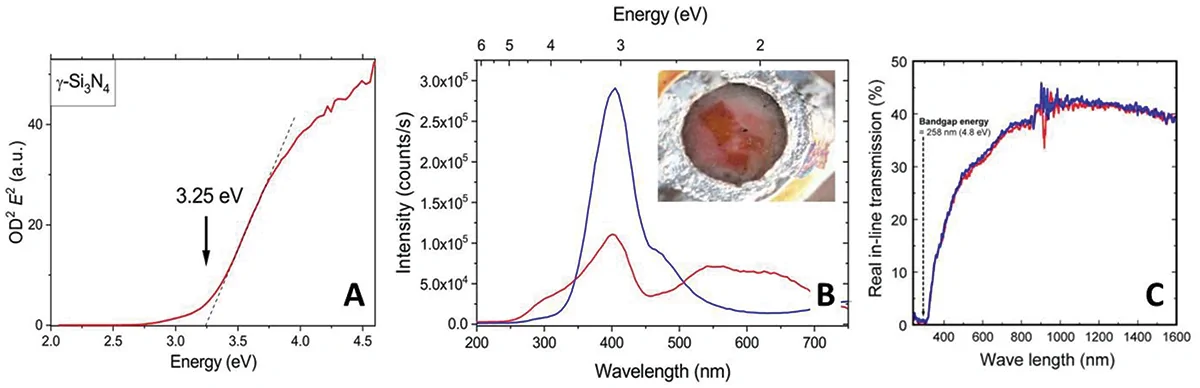
Optical transmission and CL spectra of two types of polycrystalline samples of γ-Si_3_N_4_: (A-B) powder compacted in a gasket hole via compression between the diamond anvils of a DAC; (C) transmission spectrum for a transparent sample obtained via compression and heating of α-Si_3_N_4_ in a MAA [35,231]. (A) Experimental value of the product OD^2^*E*^2^, as a function of photon energy, *E*, is shown by red solid line (see Equation 15). Dashed black line represents a linear fit to the experimental data whose interception with the abscissa would be equal to *E*_g_ provided the absorption data were not biased by any impurity- or defect states. (B) The CL spectra of the same compacted powder of γ-Si_3_N_4_ at room temperature excited by an electron beam accelerated by 20 keV. The two spectra were collected from different places on the sample surface shown in the insert which displays an optical photograph of the sample of ~100 μm in diameter after the irradiation with the electron beam. Red-brownish colouring of the initially white-greyish sample surface was observed after the irradiation with the electrons. (C) Real in-line transmission spectra measured for a transparent γ-Si_3_N_4_ sample and originally published in ref [35]. The onset of a strong absorption takes place at the photon energies of ~4.1 eV (λ=300 nm).

where *I*_0_(*E*) and *I*(*E*) are intensities of the incident and passed-through-the-sample light, respectively, at the photon energy *E*. The subsequent analysis of the absorption spectra suggested that the absorption coefficient recovered from these measurements was too low when compared with the values typical for a direct band gap. This could be attributed to passing of the incident light along grain boundaries because the cold compression in a DAC didn’t completely densify the γ-Si_3_N_4_ powder due to its high hardness. Also, the absorption at photon energies above ~3.8 eV may be caused by impurity- or defect states within the band gap while the true band gap absorption was out of the range of the set-up sensitivity [232]. Here, the contribution of oxygen contamination in the used samples of γ-Si_3_N_4_ [52] to the optical absorption and CL spectra may be the primary source. Position of the strongest peak in the CL spectra at ~3.1 eV as well as the bands between ~1.6 eV and ~2.6 eV (Figure 17B) appear to agree with the latter interpretation of the optical absorption measurements and should be attributed to states within the bad gap associated with impurities and/or defects. The luminescence onset at much higher photon energies of ~4.8 eV, indicating the *E*_g_ = 5.05(5) eV, was extracted 14 years later from the synchrotron-based photoluminescence (PL) and PL-excitation (PLE) measurements at low temperatures [233]. Transmission spectra measured for a transparent sample of γ-Si_3_N_4_, obtained from α-Si_3_N_4_ compressed to *p* = 15.6 GPa and heated to *T* = 2073 K in MAA (Figure 17C), showed the onset of a strong absorption at ~4.1 eV (300 nm) [35,231] which is still below the value from the PL-PLE measurements [233].

The element-selective soft X-ray spectroscopy (SXS) promised delivering of less uncertain results on the band-gap size when compared with the methods described above because it overcomes the limitations due to possible sample contamination with oxides and unavailability of the absolutely transparent defect-free samples. X-ray absorption near edge spectroscopy and X-ray emission spectroscopy (XANES and XES, respectively) allow probing the localised partial densities of occupied and unoccupied states near the Fermi level. For crystalline compounds, XANES and XES probe conduction band (CB) and valence band (VB), respectively. The photon energy difference between onsets of the XANES- and XES signals provides a value close to *E*_g_ which, however, should be corrected for the core-hole effect (e.g. 0.5 eV for γ-Si_3_N_4_), spin–orbit interactions, quantum confinement by examination of nm-sized grains, selective excitation of the two non-equivalent sites (e.g. tetrahedral and octahedral sites in spinel structure), broadening effects and excitonic states e.g [234–236]. Values of some of these corrections can be determined from theoretical calculations only. However, the approach does not provide any experimental information about the band-gap type, direct or indirect.

In the first SXS measurements on γ-Si_3_N_4_, the authors collected spectra for the Si L_2,3_ transition and extracted *E*_g_ = 4.30(25) eV [234]. However, the spin-orbit splitting, core-hole effect, and nonequivalent site splitting were not considered [235]. In the latter work, the SXS method was used to measure *E*_g_ values for several members of the solid solution γ-(Si_1-x_Ge_x_)_3_N_4_ with x = 0, 0.178, 0.347, 0.524, 0.875, and 1.0, stated to have been synthesised in a MAA at *p* = 23 GPa and *T* = 1773 K, except γ-Ge_3_N_4_ (x = 1.0) obtained at *p* = 12 GPa and *T* = 1473 K. According to the authors, the sample stoichiometries were measured using the EPMA technique generally accepted to be the most reliable non-destructive method of chemical analysis of small solid samples. In contrast to the previous work, the N *K*α XES and N 1*s* XANES spectra were measured in order to avoid uncertainties due to the analysis of the spectra for different cation sites (Si or Ge), because the solid solutions and the end members all contain nitrogen in tetrahedral coordination, and because there is a large number of N *p*-states in the CB and VB [235]. The obtained experimental *E*_g_ values for the solid solutions γ-(Si_1-x_Ge_x_)_3_N_4_ (red squares in Figure 18A) revealed a decreasing band gap size when the Germanium content increases from x = 0 to x = 1.0: from *E*_g_ = 4.85(20) eV for γ-Si_3_N_4_ to 3.5(2) eV for γ-Ge_3_N_4_.

**Figure 18.**
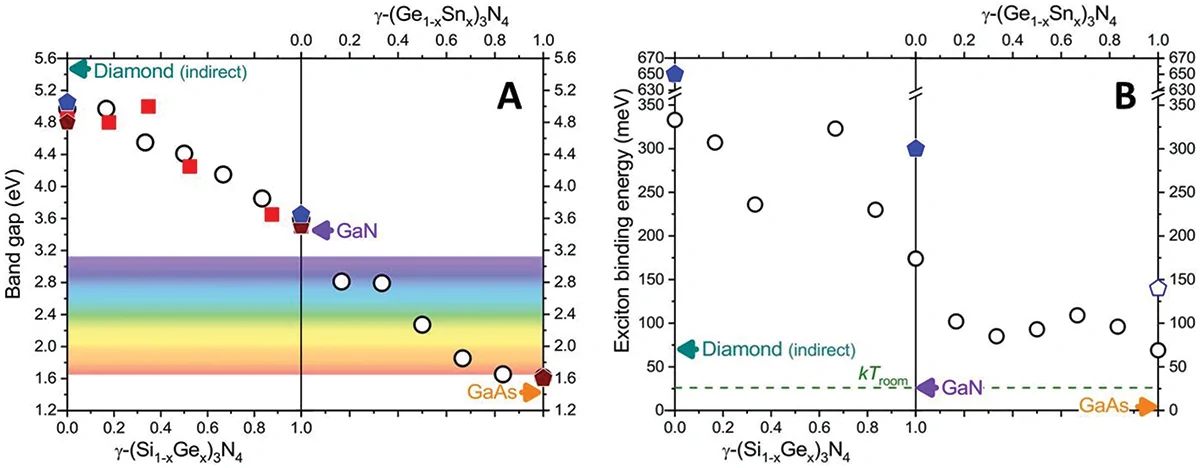
(A) Direct (or nearly direct) band gaps of nitrides of the group 14 elements having spinel structure, γ-M_3_N_4_ where M = Si, Ge, or Sn, and of the solid solutions γ-(Si_1-x_Ge_x_)_3_N_4_ and γ-(Ge_1-x_Sn_x_)_3_N_4_. Experimental *E*_g_ values from the SXS measurements are shown by solid red squares [235] and solid wine pentagons [236] while those obtained from the PL- and PLE spectroscopy are shown by solid blue pentagons [44,233]. Open black circles indicate results of theoretical calculations [236]. In the latter work, direct band gaps of the solid solutions were calculated, in a few cases, to be slightly wider than the indirect ones. Because the difference was very small and in order to avoid the figure overloading, averages of the two values were plotted here. Experimental *E*_g_ values for GaAs, GaN and diamond are shown for comparison. For the first two compounds, the band gaps are direct while that of diamond is indirect. The rainbow band spans photon energies of the visible light. (B) Exciton binding energies, *D*_e_, of nitrides of the group 14 elements, γ-M_3_N_4_ where M = Si, Ge, or Sn, and of the solid solutions γ-(Si_1-x_Ge_x_)_3_N_4_ and γ-(Ge_1-x_Sn_x_)_3_N_4_. Open black circles indicate results of the theoretical the calculations [236]. Blue solid pentagons show *D*_e_ values for γ-Si_3_N_4_ and γ-Ge_3_N_4_ obtained from the PL- and PLE spectroscopic measurements using synchrotron radiation [44,233] which are ~2 times stronger than the theoretical ones. Open blue pentagon shows an expected *D*_e_ value for γ-Sn_3_N_4_ if the tendency holds for this compound. Green horizontal dashed line shows average energy of thermal phonons at room temperature, *kT*_room_. Experimental values reported for GaAs (*E*_g_ = 1.423 eV [237] and *D*_e_ = 4.2 meV [237,238]), GaN (*E*_g_ = 3.452(1) and *D*_e_ = 26 meV [239]) and diamond (*E*_g_ = 5.470(5) and *D*_e_ = 70(4) meV [240]) are shown for comparison.

### 5.1. For energy consumption

The above described progress in understanding of electronic structure of the spinel nitrides presented results strongly supporting investment in development of technologies based on the new nitrides with the aim to substitute the presently used compounds. It appears that, in the present days of international tensions and actual state of technology, one of the most promising ways to establish new materials in the industry would be extending the products durability and efficiency. This would also lead to reduction of energy consumption needed for the products’ fabrication and application. Somewhat higher energetic and investment costs required by their production can be compensated by a significant increase of life-times of the devices and tools based on the advanced materials. An additional outcome of the latter approach would be reduction of costs for raw materials as well as collection and handling of waste. Suitability of advanced materials for applications in very different, today maybe unforeseeable, fields of application should also be considered. For example, if one and the same material can be used as a hard and wear resistant material suitable for machining tools but also for fabrication of IR-LEDs. As follows from the discussions above and below, c-Zr_3_(N,O)_4_ should be considered as a realistic candidate.

In the past, such successful transition to novel materials took place in lighting applications which were and maybe still remain the most energy consuming sector after heating/cooling and transportation. The light sources based on LEDs dominate the present-day market due to their efficiency of conversion of electric current in light which strongly exceeds that of traditional light bulbs and of fluorescence lamps requiring relatively complex and expensive electronic ballasts. After extension of optical fibres in private households, LEDs also dominate communication and visualisation electronics. Accordingly, fabrication of LEDs based on hard, wear- and chemically resistant materials (in contrast to GaAs, (Ga,In)N or ZnO) permitting tuning of the band-gap size would extend fields of their application and simplify their design. It should be also mentioned that Gallium and Indium are relatively expensive because they are less abundant: Most of Gallium is produced as a by-product of treating bauxite, an important aluminium ore. Production of Gallium is a labour- and energy-consuming process which is profitable only in low-cost countries. Due to its very low content in bauxites (50 ppm), large amounts of Gallium will never be available. Also, GaN and/or GaAs have relatively low efficiencies of transformation of electric power to light due to their low exciton binding energies, D_e_ (see below). Finally, they exhibit limited stability against hydrolysis and oxidation in air, especially at elevated temperatures.

Strong indications that some of the above-considered novel nitrides are realistic candidates for industrial applications were presented in 2013–2021. As will be shown below, application of nitrides of the group 4 and 14 elements as basic LED materials promises an increase of efficiency of conversion of electric energy in light by 10–1000 times when compared with the presently used semiconducting compounds of the groups 13 and 15 elements (also denoted as III-V compounds), e.g. GaN or GaAs. Because the novel nitrides are highly (meta)stable and their conversion efficiency remains high at elevated temperatures, the novel devices will require much less material for the same level of the light intensity as for the GaN-based compounds. Another indication of their suitability is the confirmed deposition of γ-Sn_3_N_4_ and c-Zr_3_N_4_ as thin films. The possibility to continuously tune band gaps of the nitrides and oxinitrides of the group 4 and 14 elements in a broad range of light, from near-IR to UV, is of further advantage (Figure 18). Products based on these nitrides are expected to have much simpler designs due to their high thermal stability in air making any kind of protection against oxidation or hydrolysis unnecessary. Finally, elements of groups 4 and 14 are more abundant and, accordingly, less expensive when compared with Ga.

In 2013, results of another SXS measurements of electronic structure and band gap size of the three binary nitrides of the group 14 elements recoverable to ambient conditions, namely γ-M_3_N_4_, where M = Si, Ge, or Sn was published [236]. The work also presented theoretical predictions of *E*_g_ of the binary spinel-nitrides as well as of the solid solutions γ-(Si_1-x_Ge_x_)_3_N_4_ and γ-(Ge_1-x_Sn_x_)_3_N_4_. The calculations for the end members agreed reasonably well with the *E*_g_ extracted from the SXS spectra (Figure 18A). Moreover, calculated band gaps of the two solid solutions were found to change continuously with composition thus covering a very broad range of photon energies, from near-IR- to germicidal UV light. More important were, however, predictions of very high exciton binding energy, *D*_e_, for all spinel-nitrides exceeding by several to more than 10 times the product *kT* (where *k* is Boltzmann constant) not only at room- (*T*_room_) but also at elevated temperatures (Figure 18B). The subsequent measurements of PL- and PLE spectra of γ-Si_3_N_4_ and γ-Ge_3_N_4_ did not only confirm high *D*_e_-values but showed them being ~2 times higher than the predicted ones: For γ-Si_3_N_4_, *D*_e_ was predicted to be 333 meV [236] but measured to be ~650 meV [233], for γ-Ge_3_N_4_, the same *D*_e_-values were 174 meV [236] and ~300 meV [44], respectively. Until now, *D*_e_ of γ-Sn_3_N_4_ is not measured due to experimental limitations. If the tendency holds for γ-Sn_3_N_4_, then its true *D*_e_ value should be ~140 meV or ~2 times higher than the predicted *D*_e_ = 69 meV [236], and 5.4 times higher than energy of thermal phonons at room temperature, *kT*_room_ = 26 meV. In such case, light emission from a LED should be dominated by the excitonic mechanism even for γ-Sn_3_N_4_. Upper limit of efficiency of an LED material can be estimated from the probability of a radiative decay of excitons using the equation

(16) $$\eta =\frac{R_{r}}{R_{r}+R_{d}}$$

where *R*_r_ =τ_r_^−1^ and *R*_d_=ν_T_exp(-*D*_e_/*kT*) are, respectively, radiative and thermal-dissociation rates of excitons [233]. In the case of γ-Si_3_N_4_, the measured τ_r_ ~100 ps and the estimated frequency ν_T_ = 610^13^ s^−1^ resulted in *η*(300 K)~1.0 (or 100%) [233]. Interestingly, even at the maximal temperature of stability of γ-Si_3_N_4_ (see Table 6), efficiency of the radiative decay of the excitons would be *η*(1773 K)~0.1 (or 10%) provided the parameters τ_r_ and ν_T_ remain unchanged. For comparison, GaN with τ_r_ ~295 ps and GaAs with τ_r_ ~443 ps exhibit, at room temperature, *η* ~0.015% and η~0.004%, respectively. Assuming for γ-Ge_3_N_4_ and γ-Sn_3_N_4_ the same τ_r_ and ν_T_ as for γ-Si_3_N_4_, efficiency of the radiative decays of their excitons is estimated here to be *η* ≤85% and *η* ≤2%, respectively. The latter values are much higher than those for GaN and GaAs having comparable *E*_g_ values, respectively (see Figure 18). Thus, the end-members γ-M_3_N_4_ and the solid solutions γ-(Si_1-x_Ge_x_)_3_N_4_ and γ-(Ge_1-x_,Sn_x_)_3_N_4_ could have much higher performance as basic LED materials than binary and ternary compounds of the group 13 and 15 elements (e.g. GaN, InN, GaAs, GaP) used today in the industry. Because the here-estimated *η*-values provide only upper limit for the efficiency of conversion of electric current in light, other limiting parameters such as material quality, defects, device design etc. can influence final efficiencies of the end-products. Nevertheless, the spinel nitrides appear to be suitable for production of highly efficient LEDs withstanding harsh chemical, thermal, radiative and/or mechanical attacks.

Even though fewer efforts were invested in examination of optoelectronic properties of nitrides of the group 4 elements having cubic Th_3_P_4_–type structure, c-A_3_N_4_, experimental information permitting judgment about suitability of the oxygen bearing c-Zr_3_N_4_ and c-Hf_3_N_4_ as optoelectronic materials is available. One of the reasons for the delay in examination of these compounds could be the earliest theoretical predictions that both nitrides exhibit indirect bad gaps, between 0.7 eV and 1.1 eV for c-Zr_3_N_4_ and between 1.0 eV and 1.8 eV for c-Hf_3_N_4_ [241,242]. However, more recent first-principles calculations predicted direct band gaps for these compounds but the calculated *E*_g_ values remained in the same range [243–246].

In two independent SXS experiments, band gaps of an oxygen bearing c-Zr_3_N_4_ (with the composition c-Zr_2.86_(N_0.88_O_0.12_)_4_, see Table 3) and of c-Hf_3_N_4_ were determined [247,248]. In the earlier work, the N 1s X-ray absorption and N 2p → 1s resonant X-ray emission spectra were used and *E*_g_ values of 1.6(2) eV for the oxygen-bearing c-Zr_3_N_4_ and 1.8(2) eV for c-Hf_3_N_4_ found [247]. In the later work, the measurements were extended by XANES- and XES spectra from oxygen in order to obtain the local unoccupied/occupied partial density of states not only of the N p-states but also of the O p-states [248]. In these measurements, the *E*_g_ values were found to be ~ 10% higher than in the previous ones, namely 1.75(25) eV for c-Zr_2.86_(N_0.88_O_0.12_)_4_ and 2.05(25) eV for c-Hf_3_N_4_. Moreover, Boyko et al. presented first-principles calculations of electronic structure of the end-members c-Zr_3_N_4_ and c-Hf_3_N_4_ and much more complicated modelling of the solid solution c-Zr_3−x_(N_1−x_O_x_)_4_ with x = 0.0625, 0.125, and 0.25. To perform the latter calculations, the authors had to develop a model reasonably well reproducing the structure of c-Zr_3−x_(N_1−x_O_x_)_4_ with uniformly distributed Zr vacancies and substitutional O atoms in the crystal lattice. The latter calculations have shown that *E*_g_ of c-Zr_3−x_(N_1−x_O_x_)_4_ increases from 1.47 eV to 1.84 eV when the amount of oxygen increases from x = 0 to 0.25 [248]. The calculations have also confirmed direct band gaps for all simulated compositions, including the solid solutions. Finally, an important outcome of these works is prediction of ~10 times higher *D*_e_, between 32 meV and 43 meV, when compared with *D*_e_ = 4.2 meV of the industrially used GaAs having a similar *E*_g_ = 1.423 eV [248]. If DFT calculations systematically underestimate *D*_e_ values by ~2 times, as is the case for the spinel nitrides, then cubic (oxy)nitrides of Zr and Hf having Th_3_P_4_–type structure exhibit exciton binding energies exceeding 60 meV. Accordingly, we could expect efficiencies of the energy conversion in the LEDs based on c-A_3_(N,O)_4_ to be ~ 100 times higher than those of the presently used LEDs based on GaAs. Application of these new compounds in the industry is feasible because c-Zr_3_N_4_ and, most probably, c-Hf_3_N_4_ were deposited as thin films on different substrates, including economic glass plates, using two different techniques, modified filtered cathodic arc [205] and high-power impulse magnetron sputtering (HIPIMS) [249], respectively. The only contradiction requiring clarification is a significantly higher energy of the absorption edge of the c-Hf_3_N_4_ film of 2.53 eV when compared with *E*_g_ from the above-considered SXS-measurements, between 1.8(2) eV and 2.05(25) eV.

### 5.2. For energy production

Photovoltaic (PV) technology based on silicon approached already a high level of solar energy conversion, up to 26%, close to the theoretical limit of ~30% [250]. For this reason, further efficiency improvement requires novel types of PV-cell. Also, the high energy investment needed to obtain elemental Si via reduction of SiO_2_ can be hardly compensated during lifetime of the corresponding PV-module not to mention the irreversibly consumed natural energy resources and CO_2_ emission [251]. One of the proposed solutions to increase efficiency of solar light conversion to electricity is fabrication of tandem PV-cells composed of two layers of absorbing materials with different band gaps. Silicon could remain the bottom layer while overlaying material should exhibit a wider band gap. If the latter is direct then only thin layer of the second absorber will be necessary. The possibility to deposit it on silicon or any other established PV-material via economic techniques, e.g. magnetron sputtering, would bring us closer to the solution. Even though significant progress in development of tandem PV-cells was achieved by application of organic-inorganic perovskites e.g [252], these compounds typically show limited stability when exposed to solar light, temperature variations and/or air. γ-Sn_3_N_4_ with its direct band gap of *E*_g_ = 1.6 eV appears to be suitable for the second absorber in the tandem PV-cells because its electronic structure is similar to those of such well-known PV-materials as CdTe or GaAs. The latter is considered to be the most efficient established photovoltaic material approaching, in single-junction solar cells, efficiencies up to 30%. However, it is rare (see above) and thus available only for demanding PV applications where the performance but not the price is crucial. As was shown above, *E*_g_ of γ-Sn_3_N_4_ exceeds that of silicon (*E*_g_ = 1.12 eV) and, for this reason, is well suitable for applications as the PV-cell material. Also, it should be kept in mind that highly adhesive films of γ-Sn_3_N_4_ can be deposited on silicon, borosilicate glass and other substrates via different versions of magnetron sputtering (see above Section 2.1 and below). Summarising, application of the γ-Sn_3_N_4_ as an absorber material can be of great interest as it is environmentally stable due its structure and composition; the main elements have high overall Earth abundance and are concentrated in easy accessible deposits [253]. γ-Sn_3_N_4_ can be processed and recycled with less efforts and harm for the environment. Also, most of the tin compounds are less or not soluble in water and non-toxic.

Another unexpected but promising direction of application of γ-Sn_3_N_4_ is production of hydrogen via photoelectrochemical (PEC) splitting of water (Figure 19):

(17) $$2\cdot H_{2}O\overset{\mathrm{solarlight}}{\to }2\cdot H_{2}+O_{2}$$

**Figure 19.**
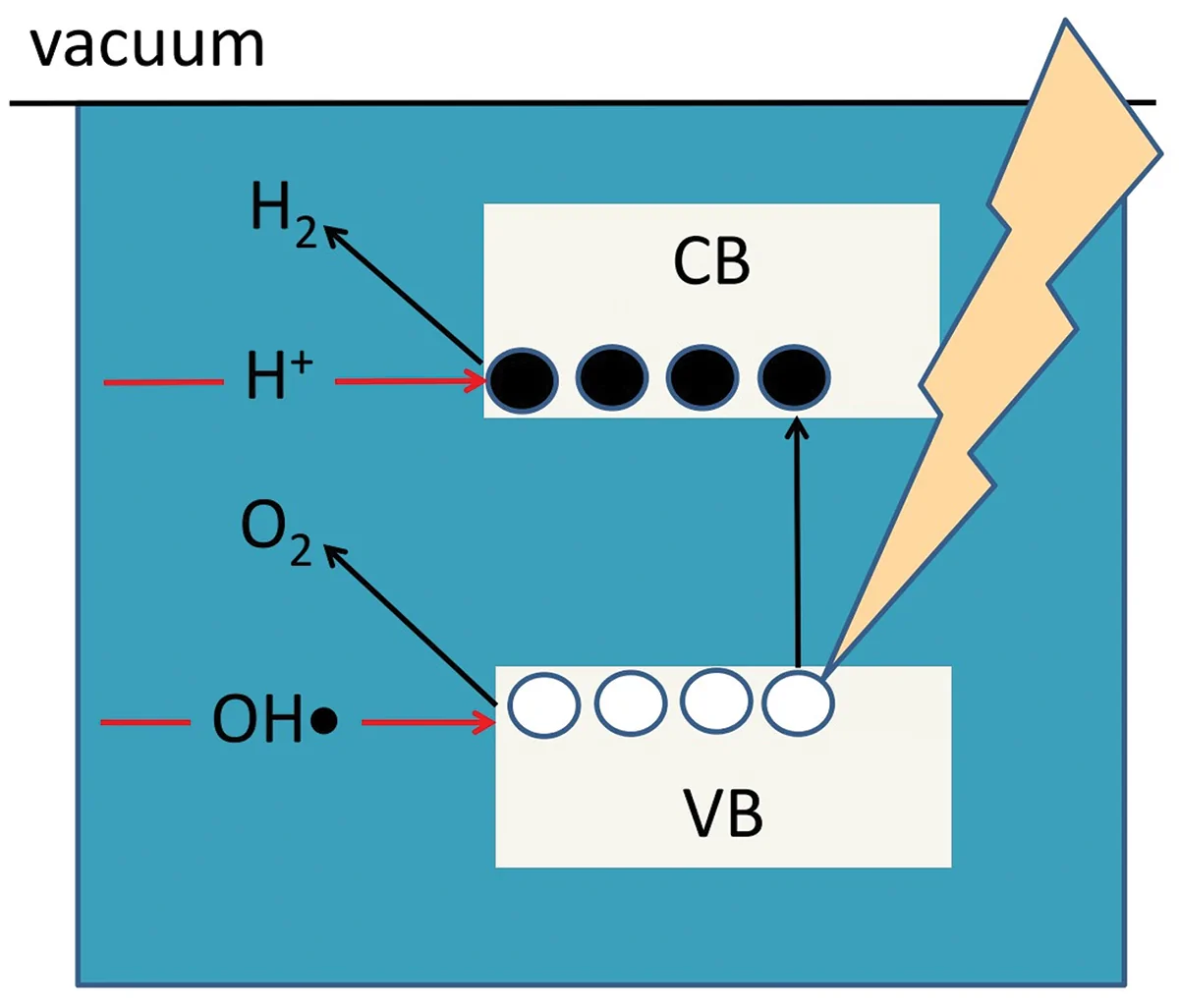
Illustration of production of ‘green’ hydrogen (and oxygen) via photoelectrochemical water splitting.

Harvesting of green hydrogen via this process attracts again strong interest in these days due to (i) abundance of water as a renewable feedstock; (ii) environmental neutrality; (iii) applicability at ambient conditions; (iv) suitability as fuel for transportation of goods with motor-lorries via the existing network of roads and highways and, maybe more important, (v) adaptable and reliable production of electricity in the periods of peak consumption which cannot be insured by other environment-friendly energy sources such as solar-cell plants and wind parks. Also, green hydrogen can be potentially stored in reservoirs and transported through pipelines used today for the natural gas. However, requirements on electronic band structure of a PEC-material strongly limit the number of potential candidates. The requirements include a direct band gap between 1.2 eV and 2.2 eV while the valence and conduction bands should match the redox potentials of water. Most of the presently considered PEC-materials contain rare- or noble elements as co-catalysts (e.g. La, Rh). Progress in investigation of different semiconductors considered to be suitable for the PEC splitting of water has been reviewed elsewhere [254]: The known drawbacks of the examined compounds were (i) only a small part of the solar spectrum could be used, e.g. just UV radiation; (ii) less suitable CV- and/or VB band positions; (iii) photo-corrosion (e. g. CdS, GaAs); (iv) production of either hydrogen or oxygen; (v) the PEC splitting efficiency was insufficient; (vi) use of rare elements; (vii) economically unviable synthesis routes. Thus, the search for PEC materials will continue until an efficient and sustainable approach for the PEC splitting of water is developed [255].

As already mentioned above, the main requirement for an efficient PEC-splitting of water is availability of semiconducting compounds having direct band gaps, *E*_g_, between 1.2 eV and 2.2 eV which valence band (VB) and conduction band (CB) straddle (or nearly straddle) the water oxidation and proton reduction potentials (~5.7 eV below vacuum for oxidation and ~4.5 eV below vacuum for reduction) which is almost never the case, e.g. Ref [256]. Since that work, published in 1998, the situation didn’t change and today there are only a few compounds with *E*_g_ < 2.2 eV showing a close band alignment with water redox potentials [257]. Such compounds should also exhibit other important properties: It should be possible to dope them to ensure *p*- and/or *n*-type conductivity and the created charge carriers should exhibit a high mobility. The specific requirement for PEC-materials is their corrosion stability during functioning in an electrolytic solution (e.g. see water). Finally, industrial production of green H_2_-fuel implies that the basic semiconducting PEC-material is easily available at low costs (which excludes use of gallium and other rare elements) and can be produced via energetically economic high-volume methods such as deposition of thin films on large surfaces made of cheap materials, e.g. ordinary glass or even plastics. The latter implies a strong adhesion of the films to substrates, also at different temperatures.

In 2015, a team of the former National Renewable Energy Laboratory (U.S.A.) confirmed deposition of the γ-Sn_3_N_4_ thin films by radio-frequency (RF) reactive sputtering using metallic Sn-targets in a nitrogen and argon atmospheres [257]. The films were found to exhibit *n*-type conductivity explained by an unintended doping with oxygen but the *p*-type conductivity was not reported. Important are energetically favourable conditions of deposition of γ-Sn_3_N_4_ films: the RF magnetron sputtering requires low powers of ~50W and the substrates were either not or just slightly heated [73]. A good adhesion of γ-Sn_3_N_4_ to different low-cost substrates such as Si-wafers and borosilicate glass were also stated. The CB of γ-Sn_3_N_4_ was measured to be 4.3–4.6 eV below vacuum thus straddling the proton reduction potential of ~4.5 eV and its VB is located at 5.9–6.0 eV below vacuum, close to the water oxidation potential of ~5.7 eV [257]. Also, a high mobility of the negative charge carriers (concentration up to 10^18^ cm^−3^) approaching 1.2 cm^2^·V^−1^·s^−1^ was measured. The latter was explained by a small electron effective mass of *m*_e_* = 0.18·*m*_*0*_ calculated using a DFT approach. The hole transport properties are not known because their diffusion length was limited by boundaries of the grains having sizes of <100 nm. Thus, larger grain sizes or reduction of the barrier potential between the grains of unknown origin should be found. The first attempt to perform electrochemical examination of the films was not successful due to an insufficient thickness of the films transforming to a passivating non-conducting layer. This suggests that thick samples or deposition of a passivating layer is needed in order to examine the electrochemical behaviour of γ-Sn_3_N_4_ [257]. The authors proposed, however, a large hole-mass of *m*_h_* = 12.9·*m*_0_ which appears to be strongly overestimated because in subsequent DFT calculations a much smaller *m*_h_*= 1.08·*m*_0_ was predicted [258].

Accordingly, experimental investigation of the electronic band structure of γ-Sn_3_N_4_ should be pursued: (i) An experimental evidence of a direct transition between its VB and CB has to be provided; (ii) precise *E*_g_ and *D*_e_ values should be determined using optical spectroscopy; (iii) influence of structural defects and impurities on electronic structure and mobility of charge carriers in γ-Sn_3_N_4_ examined.

In 2017 the same group from the National Renewable Energy Laboratory (U.S.A.) extended their search to ternary spinel-nitrides and reported on deposition of the solid solution γ-(Sn_1-x_Ti_x_)_3_N_4_ exhibiting a tuneable band gap from *E*_g_ ~2.0 eV, for x = 0.2, down to *E*_g_ < 1.0 eV, for x > 0.35 [259]. This permitted not only optimisation of positions of the VBs and CBs and tuning of *E*_g_ but also reduction of the effective hole mass, as followed from their DFT calculations, to *m*_h_*=1.4*m*_0_, for x = 2/3. At the same time, the electron effective mass remained low, *m*_e_*≤0.5*m*_0_. The authors reported a similar free carrier concentration as for γ-Sn_3_N_4_ and an improved hole mobility: Their PEC experiments with γ-(Sn_1-x_Ti_x_)_3_N_4_ films supported indirectly the theoretical prediction that the Ti substitution should decrease its hole effective mass. They have used the relative current of the photo-generated holes as a proxy for their effective masses and found that increasing the concentration of Ti leads to the photocurrent increase by as much as five times [259]. However, the measured photocurrent values could not be considered as quantitative indications of the hole mobility due to a small film thickness. Thus, bulk samples of the solid solution are required in order to quantify PEC-related properties of γ-(Sn_1-x_Ti_x_)_3_N_4_ and estimate its performance.

An experimental evidence of the existence of γ-(Sn_1-x_Ge_x_)_3_N_4_ was not provided until now. Accordingly, the potential of this compound for PEC-splitting of water follows, for small x, from the above observations for γ-Sn_3_N_4_ and from DFT calculations which predict a continuous increase of *E*_g_ from 1.6 eV (for x = 0) to 2.0 eV (for x = 0.4) and to 3.5 eV (for x = 1) [235,236]. Thus, this solid solution together with γ-(Sn_1-x_Ti_x_)_3_N_4_ covers the whole range of interest with respect to the *E*_g_-sizes. Compared with γ-Sn_3_N_4_, the VB maximum of γ-(Sn_1-x_Ge_x_)_3_N_4_ with x = 0.5 decreases by moderate ~0.6 eV while the CB minimum remains at nearly the same energy level below vacuum [258]. This moderate displacement of the VB position with respect to the water oxidation potential of ~5.7 eV is compensated by a lower *m*_h_*=0.73*m*_0_ and unaffected *m*_e_*=0.18*m*_0_ promising a high mobility of both types of charge carriers in γ-(Sn_1-x_Ge_x_)_3_N_4_ [258]. The possibility of doping was not theoretically examined for the solid solution but, as already mentioned above, potential candidates for the two related nitrides, γ-Si_3_N_4_ and γ-Ge_3_N_4_, were already proposed. The fact that the presence of oxygen appeared to be responsible for the *n*-conductivity of the γ-Sn_3_N_4_ films suggests the same element as the candidate for *n*-doping of the solid solution γ-(Sn_1-x_Ge_x_)_3_N_4_.

Summarising, γ-Sn_3_N_4_ and two solid solutions having spinel structure exhibit or are expected to exhibit properties satisfying the requirements for an efficient PEC-material. These compounds are thermodynamically stable at high pressures but can be deposited as thin films (except γ-(Sn_1-x_Ge_x_)_3_N_4_ which existence is not yet verified) via economic industrial methods. The main purpose of future research could be obtaining these compounds as macroscopic densified 3D objects with the aim to experimentally measure their mechanical and optoelectronic properties, including those needed for the PEC splitting of water. Developing of controlled *p*- and *n*-doping using chemical methods or implantation of accelerated ions is another task. Successful accomplishment of these tasks, for one of the discussed compounds at least, promises an efficient PEC-material deserving investment in large-scale tests at realistic environmental conditions.

## 6. Summary

Chemical reactions at HP-HT conditions, earlier considered as adverse effects hindering reliable measurements of properties of laser-heated solids compressed in a DAC, such as melting curves or phase boundaries of minerals and other solids e.g [260,261], became today a fruitful research direction not only in high-pressure mineralogy but also in chemistry of nitrides delivering new objects of investigation to the science of materials. Without any doubt, development of LH-DAC boosted the search for new compounds which origins, however, date back to the 1930s [262–264]. The first steps in application of LH-DAC as a chemical reactor were successful demonstrations of the capability to ignite chemical reactions and recognise the products most of which were already known. Examples of such reactions were synthesis of β-Si_3_N_4_ and of four phases of boron nitride from the elements at *p* ≤ 15 GPa [265,266], of amorphous sp^2^-bonded carbon nitrides via decomposition of an organic precursor at *p* ≤ 42 GPa [267], and of (Mg,Fe)SiO_3_ from the stoichiometric mixture of the oxides, (Mg,Fe)O and SiO_2_, at *p* ≤ 100 GPa [268] providing an ultimate evidence of thermodynamic stability of these compounds. Even the synthesis of γ-Si_3_N_4_ from the elements in a LH-DAC [46] was motivated by the quest to obtain a single-phase product despite the availability of the low-pressure α- or β- phases of Si_3_N_4_ as starting materials. Since that time, numerous reports on chemical reactions and discoveries of novel HP-HT compounds using LH-DAC, multianvil apparatuses, Belt-type apparatuses etc. were published. Methods of assessment of composition and crystal structure also developed thanks to progress of the experimental tools especially at large-scale facilities. Unfortunately, only a fragment of these achievements could be considered here.

On the other hand, reports on measurement of properties of the novel compounds recoverable at ambient conditions are much less numerous and rarely go behind measurement of their EOSs even though accompanying calculations or general considerations promise interesting properties. Experimental confirmation of the promises is, however, laborious especially if methods of property measurements approaching the industrially certified ones are considered. They pose high requirements to samples such as uniformity of composition, absence of porosity, defects, of contaminating byproducts on 1 mm-scale at least etc. Obviously, anyone would be very hesitant to take on this kind of work if not confident of achieving outstanding results. This review shows that some of the properties of interest can be revealed with reasonable investment in the sample quality using approaches lesser-known or not used in the high-pressure domain. The here-discussed examples of application of nano-indentation testing, picosecond laser ultrasonics, soft X-ray spectroscopy, or synchrotron-based PL- and PLE spectroscopy showed that at least some of the HP-HT nitrides exhibit properties deserving deeper examination requiring investment in the synthesis of bulk high-quality samples or in their deposition as thin films/coatings. Adaptation of these techniques to high-pressure samples was only possible thanks to cooperation with colleagues whose professional skills were far from the high-pressure research. It was also shown that high-pressure compounds could be of value not only as hard and/or stiff materials but offer interesting functional properties from predicted and, for this reason, expectable applications as LEDs to less foreseeable as potential catalyst for the photoelectrochemical splitting of water and production of green hydrogen.

## Note

1. According to the concept of L. Pauling, in the most general form, electronegativity is ‘the power of an atom to attract electrons to itself’ [1]. Several scales were proposed to quantify the absolute- or dimensionless relative electronegativity differences [1].

## References

[1] IUPAC Compendium of Chemical Terminology. 5.0.0 ed. International Union of Pure and Applied Chemistry (IUPAC); 2025.

[2] Simon A. Group 1 and 2 suboxides and subnitrides - metals with atomic size holes and tunnels. Coord Chem Rev. 1997;163:253–37.

[3] Gmelin L, Meyer RJ, Becker R, et al. Gmelin handbook of inorganic and organometallic chemistry. 8th ed. Weinheim, Berlin, Heidelberg, New York, London etc: Verlag Chemie, Spinger, Deutsche Nationalbibliothek; 1924.

[4] Ningthoujam RS, Gajbhiye NS. Synthesis, electron transport properties of transition metal nitrides and applications. Prog Mater Sci. 2015;70:50–154. doi: 10.1016/j.pmatsci.2014.11.004

[5] Salamat A, Hector AL, Kroll P, et al. Nitrogen-rich transition metal nitrides. Coord Chem Rev. 2013;257(13–14):2063–2072. doi: 10.1016/j.ccr.2013.01.010

[6] Dong X, Oganov AR, Cui H, et al. Electronegativity and chemical hardness of elements under pressure. Proc Natl Acad Sci. 2022;119(10):e2117416119. doi: 10.1073/pnas.2117416119 PMC8915985 35238642

[7] Neuhaus A. Synthese, Strukturverhalten und Valenzzustände der anorganischen Materie im Bereich hoher und höchster Drucke. Chimia (Aarau). 1964;18(3):93–103. doi: 10.2533/chimia.1964.93

[8] Jayaraman A, Narayanamurti V, Bucher E, et al. Continuous and discontinuous semiconductor-metal transition in samarium monochalcogenides under pressure. Phys Rev Lett. 1970;25(20):1430–1433. doi: 10.1103/PhysRevLett.25.1430

[9] Jayaraman A, Lowe W, Longinotti LD. Pressure-induced valence change in cerium phosphide. Phys Rev Lett. 1976;36(7):366–369. doi: 10.1103/PhysRevLett.36.366

[10] Zerr A, Riedel R, Sekine T, et al. Recent advances in new hard high-pressure nitrides. Adv Mater. 2006;18(22):2933–2948. doi: 10.1002/adma.200501872

[11] Joseph B, Torchio R, Benndorf C, et al. Experimental evidence of an electronic transition in CeP under pressure using Ce L_3_ XAS. Phys Chem Chem Phys. 2017;19(27):17526–17530. doi: 10.1039/C7CP03022C 28657083

[12] Ceppatelli M, Serrano-Ruiz M, Morana M, et al. High-pressure synthesis of pnictogen nitrides. Acc Chem Res. 2025;58(19):2927–2938. doi: 10.1021/acs.accounts.5c00309 PMC12509270 41004390

[13] Horvath-Bordon E, Riedel R, Zerr A, et al. High-pressure chemistry of nitride-based materials. Chem Soc Rev. 2006;35(10):987–1014. doi: 10.1039/b517778m 17003902

[14] McMillan PF, Shebanova O, Daisenberger D, et al. Metastable phase transitions and structural transformations in solid-state materials at high pressure. Phase Transitions. 2007;80(10–12):1003–1032. doi: 10.1080/01411590701473010

[15] Friedrich A, Winkler B, Juarez-Arellano EA, et al. Synthesis of binary transition metal nitrides, carbides and borides from the elements in the laser-heated diamond anvil cell and their structure-property relations. Materials. 2011;4(10):1648–1692. doi: 10.3390/ma4101648 PMC5448873 28824101

[16] Kroll P. Assessment of the Hf–N, Zr–N and Ti–N phase diagrams at high pressures and temperatures: balancing between MN and M_3_N_4_ (M = Hf, Zr, Ti). J Phys Condens Matter. 2004;16(14):S1235–S1244. doi: 10.1088/0953-8984/16/14/035

[17] Kroll P, Schröter T, Peters M. Prediction of novel phases of tantalum(v) nitride and tungsten(vi) nitride that can be synthesized under high pressure and high temperature. Angew Chem Int Ed. 2005;44(27):4249–4254. doi: 10.1002/anie.200462980 15937961

[18] Alkhaldi H, Kroll P. Chemical potential of nitrogen at high pressure and high temperature: application to nitrogen and nitrogen-rich phase diagram calculations. J Phys Chem C. 2019;123(12):7054–7060. doi: 10.1021/acs.jpcc.9b00476

[19] Vajenine GV, Auffermann G, Prots Y, et al. Preparation, crystal structure, and properties of barium pernitride, BaN_2_. Inorg Chem. 2001;40(19):4866–4870. 10.1021/ic010263+ 11531432

[20] Auffermann G, Prots Y, Kniep R. SrN and SrN_2_: diazenides by synthesis under high N_2_-pressure. Angew Chem Int Ed. 2001;40(3):547–549. doi: 10.1002/1521-3773(20010202)40:3<547::AID-ANIE547>3.0.CO;2-X 29712033

[21] Li Y, Bu H, Wang Q, et al. Synthesis and high-pressure studies of strontium diazenide by synchrotron X-ray diffraction and DFT calculations. RSC Adv. 2020;10(44):26308–26312. doi: 10.1039/D0RA00789G PMC9055401 35519776

[22] Yao Y, Adeniyi AO. Solid nitrogen and nitrogen-rich compounds as high-energy-density materials. Phys Status Solidi B. 2021;258(6):2000588. doi: 10.1002/pssb.202000588

[23] Mikhailov OV. Higher metal nitrides: current state and prospects. Russ Chem Rev. 2024;93(8):RCR5129. doi: 10.59761/RCR5129

[24] Atkins P, Jones L, Laverman L. Chemical principles. 7th ed. NY: WH Freeman and Company; 2016.

[25] Wikipedia. Le Chatelier’s principle: Wikimedia foundation, Inc; Available from: https://en.wikipedia.org/wiki/Le_Chatelier%27s_principle#cite_note-6

[26] Horvath-Bordon E, Riedel R, McMillan PF, et al. High-pressure synthesis of crystalline carbon nitride imide, C_2_N_2_(NH). Angew Chem Int Ed. 2007;46(9):1476–1480. doi: 10.1002/anie.200603851 17221897

[27] Alabdulkarim ME, Maxwell WD, Thapliyal V, et al. A comprehensive review of high-pressure laser-induced materials processing, part III: laser reactive synthesis within diamond anvil cells. J Manuf Mater Process. 2023;7(2):57. doi: 10.3390/jmmp7020057

[28] Zerr A, Miehe G, Riedel R. Synthesis of cubic zirconium and hafnium nitride having Th_3_P_4_-structure. Nat Mater. 2003;2(3):185–189. doi: 10.1038/nmat836 12612677

[29] Zerr A, Miehe G. Synthesis of tin(IV) nitride with spinel structure, γ-Sn_3_N_4_, from the elements and its Raman-spectroscopic examination at high pressures. Phil Trans R Soc A. 2023;381(2258):20220330. doi: 10.1098/rsta.2022.0330 37634541

[30] Jiang JZ, Kragh F, Frost DJ, et al. Hardness and thermal stability of cubic silicon nitride. J Phys Condens Matter. 2001;13(22):L515–L520. doi: 10.1088/0953-8984/13/22/111

[31] Zerr A, Kempf M, Schwarz M, et al. Elastic moduli and hardness of cubic silicon nitride. J Am Ceram Soc. 2002;85(1):86–90. doi: 10.1111/j.1151-2916.2002.tb00044.x

[32] Schwarz M, Zerr A, Kroke E, et al. Spinel sialons. Angew Chem Int Ed. 2002;41(5):789–793. doi: 10.1002/1521-3773(20020301)41:5<789::AID-ANIE789>3.0.CO;2-W 12491337

[33] Tanaka I, Oba F, Sekine T, et al. Hardness of cubic silicon nitride. J Mater Res. 2002;17(4):731–733. doi: 10.1557/JMR.2002.0105

[34] Shemkunas MP, Petuskey WT, Chizmeshya AVG, et al. Hardness, elasticity, and fracture toughness of polycrystalline spinel germanium nitride and tin nitride. J Mater Res. 2004;19(5):1392–1399. doi: 10.1557/JMR.2004.0186

[35] Nishiyama N, Ishikawa R, Ohfuji H, et al. Transparent polycrystalline cubic silicon nitride. Sci Rep. 2017;7(1):44755. doi: 10.1038/srep44755 PMC5355983 28303948

[36] Olson DW. Diamond (industrial). Mineral commodity summaries. Reston (VA): U. S. Geological Survey; 2011. p. 50–51.

[37] Bailey MW, Davies GJ, Burns RC, et al. Diamond materials and their applications. In: Riedel R, editor. Handbook of ceramic hard materials. Vol. 2. Weinheim: Wiley-VCH; 2000. p. 479–572.

[38] Wang Z, Shi L. Xinhua headlines: made-in-china diamonds poised to shape global market Xinhua. 2019 [Available from: https://www.xinhuanet.com/english/2019-02/06/c_137803170.htm

[39] DeVries RC. Inventory on innovative research: the case of C_3_N_4_. Mater Res Innovat. 1997;1(3):161–162. doi: 10.1007/s100190050035

[40] Malkow T. Critical observations in the research of carbon nitride. Mater Sci Eng A. 2000;292(1):112–124. doi: 10.1016/S0921-5093(00)00960-6

[41] Chaudhri MM, Lim YY. Harder than diamond? Just fiction. Nat Mater. 2005;4(1):4. doi: 10.1038/nmat1288

[42] Taniguchi T. Materials science research of boron nitride obtained at high pressure and high temperature. J Ceram Soc Jpn. 2020;128(9):620–626. doi: 10.2109/jcersj2.20116

[43] Tebbe D, Schütte M, Watanabe K, et al. Tailoring the dielectric screening in WS_2_–graphene heterostructures. npj 2D Mater Appl. 2023;7(1):29. doi: 10.1038/s41699-023-00394-0

[44] Feldbach E, Zerr A, Museur L, et al. Electronic band transitions in γ-Ge_3_N_4_. Electron Mater Lett. 2021;17(4):315–323. doi: 10.1007/s13391-021-00291-y

[45] Li C, Djemia P, Chigarev N, et al. Elastic moduli and refractive index of γ-Ge_3_N_4_. Phil Trans R Soc A. 2023;381(2258):20230016. doi: 10.1098/rsta.2023.0016 37634530

[46] Zerr A, Miehe G, Serghiou G, et al. Synthesis of cubic silicon nitride. Nature. 1999;400(6742):340–342. doi: 10.1038/22493

[47] Serghiou G, Miehe G, Tschauner O, et al. Synthesis of a cubic Ge_3_N_4_ phase at high pressures and temperatures. J Chem Phys. 1999;111(10):4659–4662. doi: 10.1063/1.479227

[48] Scotti N, Kockelmann W, Senker J, et al. Sn_3_N_4_, a tin(IV) nitride - syntheses and the first crystal structure determination of a binary tin-nitrogen compound. Z Anorg Allg Chem. 1999;625:1435–1439.

[49] Leinenweber K, O’Keeffe M, Somayazulu M, et al. Synthesis and structure refinement of the spinel, γ-Ge_3_N_4_. Chem Eur J. 1999;5(10):3076–3078. doi: 10.1002/(SICI)1521-3765(19991001)5:10<3076::AID-CHEM3076>3.0.CO;2-D

[50] Somayazulu MS, Leinenweber K, Hubert H, et al. High pressure - high temperature synthesis of spinel Ge_3_N_4_. In: Manghnani M, Nellis WJ Nicol M, editors. Science and Technology of High Pressure, Proceedings of AIRAPT-17; 25–30 July, 1999; Honolulu, (HI), USA:. Universities Press, Hyderabad, India; 2000. p. 663–666.

[51] Zerr A, Miehe G, Serghiou G, et al. A new cubic silicon nitride phase. In: Manghnani M, Nellis WJ, Nicol M, editors. International Conference on High Pressure Science and Technology (AIRAPT-17). Honolulu, (HI), USA. Universities Press (India); 1999 July 25–30; p. 914–917.

[52] Schwarz M, Miehe G, Zerr A, et al. Spinel-Si_3_N_4_: multi-anvil press synthesis and structural refinement. Adv Mater. 2000;12(12):883–887. doi: 10.1002/1521-4095(200006)12:12<883::AID-ADMA883>3.0.CO;2-C

[53] Jiang JZ, Stahl K, Berg RW, et al. Structural characterisation of cubic silicon nitride. Europhys Lett. 2000;51(1):62–67. doi: 10.1209/epl/i2000-00337-8

[54] Soignard E, Somayazulu M, Dong J, et al. High pressure - high temperature synthesis and elasticity of the cubic nitride spinel γ-Si_3_N_4_. J Phys Condens Matter. 2001;13(4):557–563. doi: 10.1088/0953-8984/13/4/302

[55] Nishiyama N, Fujii K, Kulik E, et al. Thermal expansion and P-V-T equation of state of cubic silicon nitride. J Eur Ceram Soc. 2019;39(13):3627–3633. doi: 10.1016/j.jeurceramsoc.2019.05.003

[56] Sekine T, He H, Kobayashi T, et al. Shock-induced transformtion of β-Si_3_N_4_ to a high-pressure cubic-spinel phase. Appl Phys Lett. 2000;76(25):3706–3708. doi: 10.1063/1.126756

[57] Blank VD, Deribas AA, Lvova NA, et al. Properties of cubic Si_3_N_4_ obtained by shock synthesis. Mater Sci Forum. 2008;566:129–134.

[58] Köhler A, Schlothauer T, Schimpf C, et al. The role of oxygen in shockwave-synthesized γ-Si_3_N_4_ material. J Eur Ceram Soc. 2015;35(12):3283–3288. doi: 10.1016/j.jeurceramsoc.2015.05.009

[59] Zhang M, He H, Xu FF, et al. High-resolution transmission electron microscopy of cubic Si_3_N_4_. J Appl Phys. 2000;88(5):3070–3072. doi: 10.1063/1.1287765

[60] Gross T, Schwarz M, Knapp M, et al. Thermal expansion study of spinel-sialon. J Eur Ceram Soc. 2007;27(5):2163–2169. doi: 10.1016/j.jeurceramsoc.2006.07.007

[61] He H, Sekine T, Kobayashi T, et al. Phase transformation of germanium nitride (Ge_3_N_4_) under shock wave compression. J Appl Phys. 2001;90(9):4403–4406. doi: 10.1063/1.1407851

[62] Li X, Hector AL, Owen JR, et al. Evaluation of nanocrystalline Sn_3_N_4_ derived from ammonolysis of Sn(NEt_2_)_4_ as a negative electrode material for Li-ion and Na-ion batteries. J Mater Chem A. 2016;4(14):5081–5087. doi: 10.1039/C5TA08287K

[63] Nand SV, Ankur K, Brijesh K, et al. Synthesis of single phase cubic tin nitride nanoparticles by atmospheric pressure–halide vapor phase epitaxy. Solid State Sci. 2008;10(5):569–572. doi: 10.1016/j.solidstatesciences.2007.10.007

[64] Shemkunas MP, Wolf GH, Leinenweber K, et al. Rapid synthesis of crystalline spinel tin nitride by a solid-state metathesis reaction. J Am Ceram Soc. 2002;85(1):101–104. doi: 10.1111/j.1151-2916.2002.tb00047.x

[65] Pradhan GK, Kumar A, Deb SK, et al. Elastic and structural instability of cubic Sn_3_N_4_ and C_3_N_4_ under pressure. Phys Rev B. 2010;82(14):144112. doi: 10.1103/PhysRevB.82.144112

[66] Qu FD, Yuan Y, Yang MH. Programmed synthesis of Sn_3_N_4_ nanoparticles via a soft chemistry approach with urea: application for ethanol vapor sensing. Chem Mat. 2017;29(3):969–974.

[67] Fitch SDS, Cibin G, Hepplestone SP, et al. Solvothermal synthesis of Sn_3_N_4_ as a high capacity sodium-ion anode: theoretical and experimental study of its storage mechanism. J Mater Chem A. 2020;8(32):16437. doi: 10.1039/D0TA04034G

[68] Zipkat M, Koldemir A, Block T, et al. Scalable bulk synthesis of phase-pure γ-Sn_3_N_4_ as a model for an argon-flow-mediated metathesis reaction. Chem Eur J. 2025;31(10):e202403745. doi: 10.1002/chem.202403745 PMC11833219 39556403

[69] Nishiyama N, Kitani S, Ohta Y, et al. Low temperature heat capacity measurements of β-Si_3_N_4_ and γ-Si_3_N_4_: determination of the equilibrium phase boundary between β-Si_3_N_4_ and γ-Si_3_N_4_. J Eur Ceram Soc. 2020;40(16):6309–6315. doi: 10.1016/j.jeurceramsoc.2019.11.025

[70] Nishiyama N, Langer J, Sakai T, et al. Phase relations in silicon and germanium nitrides up to 98GPa and 2400 degrees C. J Am Ceram Soc. 2019;102(4):2195–2202. doi: 10.1111/jace.16063

[71] Bhat S, Wiehl L, Haseen S, et al. A novel high-pressure tin oxynitride Sn_2_N_2_O. Chem Eur J. 2020;26(10):2187–2194. doi: 10.1002/chem.201904529 PMC7065226 31671223

[72] Lima RS, Dionisio PH, Schreiner WH, et al. Magnetron sputtered tin nitride. Solid State Commun. 1991;79(5):395–398. doi: 10.1016/0038-1098(91)90491-D

[73] Maruyama T, Morishita T. Tin nitride thin films prepared by radio-frequency reactive sputtering. J Appl Phys. 1995;77(12):6641–6645. doi: 10.1063/1.359075

[74] Inoue Y, Nomiya M, Takai O. Physical properties of reactive sputtered tin-nitride thin films. Vacuum. 1998;51(4):673–676. doi: 10.1016/S0042-207X(98)00271-1

[75] Soignard E, McMillan PF, Leinenweber K. Solid solutions and ternary compound formation among Ge_3_N_4_-Si_3_N_4_ nitride spinels synthesized at high pressure and high temperature. Chem Mater. 2004;16(25):5344–5349. doi: 10.1021/cm048930v

[76] Schwarz M. High pressure synsthesis of novel hard materials: spinel-Si_3_N_4_ and derivates. Kassel: Kassel University Press, TU Darmstadt; 2003.

[77] Schwarz MR, Antlauf M, Schmerler S, et al. Formation and properties of rocksalt-type AlN and implications for high pressure phase relations in the system Si-al-O-N. High Press Res. 2014;34(1):22–38.

[78] Holleman AF, Wiberg E, Wiberg N. Lehrbuch der anorganischen Chemie. Berlin: Walter de Gruyter; 1995.

[79] Sekine T, He H, Kobayashi T, et al. Cubic Si_6-z_Al_z_O_z_N_8-z_ (z = 1.8 and 2.8) spinels formed by shock compresison. Chem Phys Lett. 2001;344(3–4):395–399. doi: 10.1016/S0009-2614(01)00789-8

[80] Tatsumi K, Mizoguchi T, Yoshioka S, et al. Distribution of solute atoms in β- and spinel Si_6-z_Al_z_O_z_N_8-z_ by Al *K*-edge x-ray absorption near-edge structure. Phys Rev B. 2005;71(3):033202. doi: 10.1103/PhysRevB.71.033202

[81] Bhadram VS, Liu HY, Xu ES, et al. Semiconducting cubic titanium nitride in the Th_3_P_4_ structure. Phys Rev Mater. 2018;2(1):011602(R). doi:10.1103/PhysRevMaterials.2.011602

[82] Dzivenko D, Zerr A, Miehe G, et al. Synthesis and properties of oxygen bearing c-Zr_3_N_4_ and c-Hf_3_N_4_. J Alloys Comp. 2009;480(1):46–49. doi: 10.1016/j.jallcom.2008.10.011

[83] Kawamura F, Shibazaki Y, Yusa H, et al. High-pressure synthesis of Th_3_P_4_-type c-Zr_3_N_4_ using a metathesis reaction and in situ X-ray diffraction studies of the formation process. Cryst Growth Des. 2023;23(4):2504–2510. doi: 10.1021/acs.cgd.2c01433

[84] Dzivenko DA, Zerr A, Bulatov VK, et al. High-pressure multianvil synthesis and structure refinement of oxygen-bearing cubic zirconium(IV) nitride. Adv Mater. 2007;19(14):1869–1873. doi: 10.1002/adma.200602610

[85] Yajima A, Segawa Y, Matsuzaki R, et al. Reaction process of zirconium tetrachloride with ammonia in the vapor phase and properties of the zirconium nitride formed. Bull Chem Soc Jpn. 1983;56(9):2638–2642. doi: 10.1246/bcsj.56.2638

[86] Yajima A, Akiyama M, Saeki Y. Formation process of hafnium nitride by the reaction of hafnium tetrachloride with ammonia in the vapor phase and properties of the hafnium nitride formed. Nippon Kagaku Kaishi. 1986;1986(9):1175–1180. doi: 10.1246/nikkashi.1986.1175

[87] Lerch M, Füglein E, Wrba J. Synthesis, crystal structure, and high temperature behavior of Zr_3_N_4_. Z Anorg Allg Chem. 1996;622(2):367–372. doi: 10.1002/zaac.19966220227

[88] Li J, Dzivenko D, Zerr A, et al. Synthesis of nanocrystalline Zr_3_N_4_ and Hf_3_N_4_ powders from metal dialkylamides. Z Anorg Allg Chem. 2005;631(8):1449–1455. doi: 10.1002/zaac.200400539

[89] Taniguchi T, Dzivenko D, Riedel R, et al. Synthesis of cubic zirconium(IV) nitride, c-Zr_3_N_4_, in the 6–8 GPa pressure region. Ceram Int. 2019;45(16):20028–20032.

[90] Anzellini S, Monteseguro V, Bandiello E, et al. In situ characterization of the high pressure – high temperature melting curve of platinum. Sci Rep. 2019;9(1):13034. doi: 10.1038/s41598-019-49676-y PMC6736956 31506567

[91] Brauer G, Zapp KH. Die Nitride des Tantals. Z Anorg Allg Chem. 1954;277(3–4):129–139. doi: 10.1002/zaac.19542770304

[92] Brauer G, Kern W. Zersetrungsdrücke und Phasengrenzenvon Niobnitriden. Z Anorg Allg Chem. 1983;507(12):127–141. doi: 10.1002/zaac.19835071216

[93] Fix R, Gordon RG, Hoffman DM. Chemical vapor deposition of vanadium, niobium, and tantalum nitride thin films. Chem Mater. 1993;5(5):614–619. doi: 10.1021/cm00029a007

[94] Ganin AY, Kienle L, Vajenine GV. Plasma-enhanced CVD synthesis and structural characterization of Ta_2_N_3_. Eur J Inorg Chem. 2004;2004(16):3233–3239. doi: 10.1002/ejic.200400227

[95] Moureu H, Hamblet CH. The ammonolysis of tantalum pentachloride. J Am Chem Soc. 1937;59(1):33–40. doi: 10.1021/ja01280a009

[96] Funk H, Bohland H. Zur Darstellung von Metallnitriden aus Ammoniumfluormetallaten und Ammoniak. Z Anorg Allg Chem. 1964;334(3–4):155–162.

[97] Tessier F, Assabaa R, Marchand R. Mixed valent niobium nitrides and oxynitrides resulting from ammonolysis of alkaline niobates. J Alloys Compd. 1997;262–263:512–515. doi: 10.1016/S0925-8388(97)00365-4

[98] Gilles JC. Sur deux nouveaux nitrures de tantale. C R Acad Sc Paris. 1968;266:546–547.

[99] Brauer G, Weidlein J, Strähle J. Über das Tantalnitrid Ta_3_N_5_ und das Tantaloxidnitrid TaON. Z Anorg Allg Chem. 1966;348(5–6):298–308. doi: 10.1002/zaac.19663480511

[100] Brese NE, O’Keeffe M, Rauch P, et al. Structure of Ta_3_N_5_ at 16 K by time-of-flight neutron diffraction. Acta Cryst C. 1991;47(11):2291–2294. doi: 10.1107/S0108270191005231

[101] Strähle J. Die Kristallstruktur des Tantal(V)-nitrids Ta_3_N_5_. Z Anorg Allg Chem. 1973;402(1):47–57. doi: 10.1002/zaac.19734020107

[102] Hitoki G, Ishikawa A, Takata T, et al. Ta3N5 as a novel visible light-driven photocatalyst (λ<600>. Chem Lett. 2002;31(7):736–737. doi: 10.1246/cl.2002.736

[103] Ishikawa A, Takata T, Kondo JN, et al. Electrochemical behavior of thin Ta_3_N_5_ semiconductor film. J Phys Chem B. 2004;108(30):11049–11053. doi: 10.1021/jp048802u

[104] Higashi M, Domen K, Abe R. Fabrication of efficient TaON and Ta_3_N_5_ photoanodes for water splitting under visible light irradiation. Energy Environ Sci. 2011;4(10):4138–4147. doi: 10.1039/c1ee01878g

[105] Yusa H, Kawamura F, Taniguchi T, et al. High-pressure synthesis and compressive behavior of tantalum nitrides. J Appl Phys. 2014;115(10):103520. doi: 10.1063/1.4867986

[106] Terao N. Structure of tantalum nitrides. Jpn J Appl Phys. 1971;10(2):248–259. doi: 10.1143/JJAP.10.248

[107] Terao N. New phases of niobium nitride. J Less-Common Met. 1971;23(2):159–169. doi: 10.1016/0022-5088(71)90076-2

[108] Zerr A, Miehe G, Li J, et al. High-pressure synthesis of tantalum nitride having orthorhombic U_2_S_3_ structure. Adv Funct Mater. 2009;19(14):2282–2288. doi: 10.1002/adfm.200801923

[109] Asano S, Niwa K, Sasaki T, et al. Thermal expansion of incompressible U_2_S_3_-type Nb_2_N_3_ synthesized in a diamond anvil cell. Inorg Chem. 2020;59(12):7915–7918. doi: 10.1021/acs.inorgchem.0c01057 32463665

[110] Friedrich A, Winkler B, Bayarjargal L, et al. In situ observation of the reaction of tantalum with nitrogen in a laser heated diamond anvil cell. J Alloys Comp. 2010;502(1):5–12. doi: 10.1016/j.jallcom.2010.04.113

[111] Bykov M, Bykova E, Ponomareva AV, et al. Stabilization of polynitrogen anions in tantalum–nitrogen compounds at high pressure. Angew Chem Int Ed. 2021;60(16):9003–9008. doi: 10.1002/anie.202100283 33556216

[112] Salamat A, Woodhead K, Shah SIU, et al. Synthesis of U_3_Se_5_ and U_3_Te_5_ type polymorphs of Ta_3_N_5_ by combining high pressure-temperature pathways with a chemical precursor approach. Chem Commun. 2014;50(70):10041–10044. 10.1039/c4cc05147e 25051155

[113] Chen H, Bykov M, Batyrev IG, et al. Synthesis and stability of high-energy-density niobium nitrides under high-pressure conditions. Inorg Chem. 2025;64(1):692–700. doi: 10.1021/acs.inorgchem.4c03331 39707975

[114] Asano S, Niwa K, Sasaki T, et al. High pressure synthesis and the valence state of vanadium ions for the novel transition metal pernitride, CuAl_2_-type VN_2_. Dalton Trans. 2022;51(7):2656–2659. doi: 10.1039/D1DT04310B 35106526

[115] Ming L, Bassett WA. Laser heating in the diamond anvil press up to 2000°C sustained and 3000°C pulsed at pressures up to 260 kilobars. Rev Sci Instrum. 1974;45(9):1115–1118. doi: 10.1063/1.1686822

[116] Heinz DL, Sweeney JS, Miller P. A laser heating system that stabilizes and controls the temperature: diamond anvil cell applications. Rev Sci Instrum. 1991;62(6):1568–1575. doi: 10.1063/1.1142434

[117] Boehler R, Chopelas A. A new approach to laser heating in high pressure mineral physics. Geophys Res Lett. 1991;18(6):1147–1150. doi: 10.1029/91GL01144

[118] Zerr A, Serghiou G, Boehler R. CO_2_-laser heating in a diamond cell to 80 GPa and 5000 K: phase transitions and synthesis under controlled conditions. In: Kamo M, editor. The 5th NIRIM International Symposium on Advanced Materials (ISAM’98); Tsukuba, Japan: NIRIM; 1998 Mar 1–5; p. 5–8.

[119] Shen G, Rivers ML, Wang Y, et al. Laser heated diamond cell system at the advanced photon source for in situ X-ray measurements at high pressure and temperature. Rev Sci Instrum. 2001;72(2):1273–1282. doi: 10.1063/1.1343867

[120] Kavner A, Panero WR. Temperature gradients and evaluation of thermoelastic properties in the synchrotron-based laser-heated diamond cell. Phys Earth Planet Inter. 2004;143–144:527–539. doi: 10.1016/j.pepi.2003.12.016

[121] Salamat A, Fischer RA, Briggs R, et al. In situ synchrotron X-ray diffraction in the laser-heated diamond anvil cell: melting phenomena and synthesis of new materials. Coord Chem Rev. 2014;277:15–30.

[122] Anzellini S, Boccato S. A practical review of the laser-heated diamond anvil cell for university laboratories and synchrotron applications. Crystals. 2020;10(6):459. doi: 10.3390/cryst10060459

[123] Morard G, Boccato S, Rosa AD, et al. Solving controversies on the iron phase diagram under high pressure. Geophys Res Lett. 2018;45(20):11074–11082. doi: 10.1029/2018GL079950

[124] Prakapenka VB, Shen GY, Dubrovinsky LS. Carbon transport in diamond anvil cells. High Temp-High Press. 2003;35–6(2):237–249.

[125] Liermann HP, Konopkova Z, Appel K, et al. Novel experimental setup for megahertz X-ray diffraction in a diamond anvil cell at the high energy density (HED) instrument of the European X-ray free-electron laser (EuXFEL). J Synchrot Radiat. 2021;28(3):688–706. doi: 10.1107/S1600577521002551 PMC8127375 33949979

[126] Hong C, Deng Y, Xu F, et al. Pressure dependence of sound velocity, shear modulus, and yield strength of the amorphous 4: 1 methanol-ethanol mixture to 78 GPa. Phys Rev B. 2025;111(18):184110. doi: 10.1103/PhysRevB.111.184110

[127] Gandolfi G. Discussion upon methods to obtain X-ray «powder patterns» from a single crystal. Min Petrogr Acta. 1967;13:67–74.

[128] Kawamura F, Yusa H, Taniguchi T. Synthesis of rhenium nitride crystals with MoS_2_ structure. Appl Phys Lett. 2012;100(25):251910. doi: 10.1063/1.4729586

[129] Yusa H, Hirao N, Mori Y, et al. A multi-axis diamond anvil cell oscillation system for powder X-ray diffraction to improve statistics and analyze deviatoric stress. In: Saraiva J, chairman. 56th European High Pressure Research Group Meeting; Aveiro, Portugal: Universidade de Aveiro; 2018. p. 338.

[130] Laniel D, Bykov M, Fedotenko T, et al. Supplementary materials. High pressure investigation of the S-N_2_ system up to the megabar range: synthesis and characterization of the SN_2_ solid. Inorg Chem. 2019;58(14):S1–S4. doi: 10.1021/acs.inorgchem.9b00830 31247816

[131] Zerr A, Eschnauer H, Kny E. Hard materials. In: Elvers B, editor. Ullmann’s encyclopedia of industrial chemistry, electronic release. Weinheim/Germany: Wiley-VCH; 2012. p. 1–21.

[132] Hall HT. Ultra-high-pressure, high-temperature apparatus: the “Belt”. Rev Sci Instrum. 1960;31(2):125–131.

[133] Fukunaga O, Ko Y, Konoue M, et al. Pressure and temperature control in flat-belt type high pressure apparatus for reproducible diamond synthesis. Diamond Relat Mater. 1999;8(11):2036–2042. doi: 10.1016/S0925-9635(99)00171-5

[134] Taniguchi T, Akaishi M, Kanke Y, et al. TiC-diamond composite disk-heater cell assembly to generate temperature of 2000 °C in a large-volume belt-type high-pressure apparatus at 10GPa. Rev Sci Instrum. 2004;75(6):1959–1962. doi: 10.1063/1.1753092

[135] Hall HT. Anvil guide device for multiple-anvil high pressure apparatus. Rev Sci Instrum. 1962;33(11):1278–1280. doi: 10.1063/1.1717748

[136] Kawai N, Endo S. The generation of ultrahigh hydrostatic pressures by a split sphere apparatus. Rev Sci Instrum. 1970;41(8):1178–1181. doi: 10.1063/1.1684753

[137] Walker D, Carpenter MA, Hitch CM. Some simplifications to multianvil devices for high pressure experiments. Am Mineral. 1990;75:1020–1028.

[138] Ito E. 2.08 - theory and practice - multianvil cells and high-pressure experimental methods. In: Schubert G Price GD, editors. Treatise on geophysics. Vol. 2. Amsterdam: Elsevier B.V.; 2007. p. 197–230.

[139] Liebermann RC. Multi-anvil, high pressure apparatus: a half-century of development and progress. High Press Res. 2011;31(4):493–532. doi: 10.1080/08957959.2011.618698

[140] Klotz S, Gauthier M, Besson J, et al. Techniques for neutron diffraction on solidified gases to 10 GPa and above: applications to ND_3_ phase IV. Appl Phys Lett. 1995;67(9):1188–1190. doi: 10.1063/1.115002

[141] Lipp MJ, Evans WJ, Yoo CS. Cryogenic loading of large volume presses for high-pressure experimentation and synthesis of novel materials. Rev Sci Instrum. 2005;76(5):053903. doi: 10.1063/1.1900644

[142] Voigt W. Lehrbuch der Kristallphysik. Leipzig: Teubner; 1928.

[143] Reuss A. Berechnung der Fließgrenze von Mischkristallen auf Grund der Plastizitätsbedingung für Einkristalle. Z Angew Math Mech. 1929;9(1):49–58. doi: 10.1002/zamm.19290090104

[144] Hill R. The elastic behaviour of a crystalline aggregate. Proc Phys Soc A. 1952;65(5):349–354. doi: 10.1088/0370-1298/65/5/307

[145] Hashin Z, Shtrikman S. On some variational principles in anisotropic and nonhomogeneous elastisity. J Mech Phys Solids. 1962;10(4):335–342. doi: 10.1016/0022-5096(62)90004-2

[146] Hashin Z, Shtrikman S. A variational approach to the theory of the elastic behavior of polycrystals. J Mech Phys Solids. 1962;10(4):343–352. doi: 10.1016/0022-5096(62)90005-4

[147] Angel RJ. Equations of state. Rev Mineral Geochem. 2000;41(1):35–59. doi: 10.2138/rmg.2000.41.2

[148] Angel RJ, Alvaro M, Nestola F. 40 years of mineral elasticity: a critical review and a new parameterisation of equations of state for mantle olivines and diamond inclusions. Phys Chem Miner. 2018;45(2):95–113. doi: 10.1007/s00269-017-0900-7

[149] Birch F. Finit-strain isotherm and velocities for single-crystal and polycrystalline NaCl at high pressure and 300° K. J Geophys Res. 1978;75(B3):1257–1268. doi: 10.1029/JB083iB03p01257

[150] He H, Sekine T, Kobayashi T, et al. Shock-induced phase transition of β-Si_3_N_4_ to c-Si_3_N_4_. Phys Rev B. 2000;62(17):11412–11417. doi: 10.1103/PhysRevB.62.11412

[151] Zhang XQ, Cui SW, Ma SL, et al. Hardness, elastic modulus and their correlations in the transparent silicon nitrides. Mater Today Commun. 2024;38:108320. doi: 10.1016/j.mtcomm.2024.108320

[152] Kearney JSC, Grauzinyte M, Smith D, et al. Pressure-tuneable visible-range band gap in the ionic spinel tin nitride. Angew Chem Int Edit. 2018;57(36):11623–11628. doi: 10.1002/anie.201805038 PMC6221123 30022577

[153] Niwa K, Inagaki T, Ohsuna T, et al. Crystal structures and electronic properties of Sn_3_N_4_ polymorphs synthesized via high-pressure nitridation of tin. Cryst Eng Comm. 2020;22(20):3531–3538. doi: 10.1039/D0CE00210K

[154] Dzivenko DA, Zerr A, Schweitzer E, et al. Elastic moduli and hardness of c-Zr_2.86_(N_0.88_O_0.12_)_4_ having Th_3_P_4_-type structure. Appl Phys Lett. 2007;90(19):191910. doi: 10.1063/1.2738373

[155] Zerr A, Chigarev N, Brenner R, et al. Elastic moduli of hard c-Zr_3_N_4_ from laser ultrasonic measurements. Phys Status Solidi-Rapid Res Lett. 2010;4(12):353–355. doi: 10.1002/pssr.201004345

[156] Dzivenko DA, Zerr A, Boehler R, et al. Equation of state of cubic hafnium(IV) nitride having Th_3_P_4_-type structure. Solid State Commun. 2006;139(6):255–258. doi: 10.1016/j.ssc.2006.06.020

[157] Zerr A, Chigarev N, Brinza O, et al. Elastic moduli of η-Ta_2_N_3_, a tough self-healing material, via laser ultrasonics. Phys Status Solidi-Rapid Res Lett. 2012;6(12):484–486. doi: 10.1002/pssr.201206419

[158] Bourguille J, Brinza O, Tetard F, et al. Elastic moduli and hardness of η-Ta_2_N_3_ from nanoindentation measurements. EPL. 2015;111(1):18006. doi: 10.1209/0295-5075/111/18006

[159] Zerr A. Comments on “hardness, elasticity, and fracture toughness of polycrystalline spinel germanium nitride and tin nitride,” by MP Shemkunas, WT petuskey, AVG Chizmeshya, K. Leinenweber, and GH wolf J. Mater. Res. 19, 1392 (2004): reestablishing of elastic moduli for γ-Ge_3_N_4_ [editorial material]. J Mater Res. 2008;23(12):3273–3274.

[160] Eremets M. High pressure experimental methods. Oxford, UK: Oxford University Press; 1996.

[161] Xu F, Belliard L, Li C, et al. Single-crystal elastic moduli, anisotropy, and the B1–B2 phase transition of NaCl at high pressures. Appl Phys Lett. 2024;125(16):164102. doi: 10.1063/5.0231544

[162] Deng Y, Zhang J, Xu F, et al. Sound velocities, shear modulus, and yield strength of tungsten to 141 GPa. Appl Phys Lett. 2026;128(12). doi: 10.1063/5.0315148

[163] Andearson OL. The role of experimental physical acoustics in geophysics. In: Ritsema AR, editor. The upper Mantle. Tectonophysics. Vol. 13(1–4). Amsterdam: Elsevier Publishing Company; 1972. p. 521–540.

[164] Angel RJ, Jackson JM, Reichmann HJ, et al. Elasticity measurements on minerals: a review. Eur J Mineral. 2009;21(3):525–550. doi: 10.1127/0935-1221/2009/0021-1925

[165] Wolff C, Smith MJA, Stiller B, et al. Brillouin scattering—theory and experiment: tutorial. J Opt Soc Am B. 2021;38(4):1243–1269. doi: 10.1364/JOSAB.416747

[166] Sinogeikin S, Bass J, Prakapenka V, et al. Brillouin spectrometer interfaced with synchrotron radiation for simultaneous x-ray density and acoustic velocity measurements. Rev Sci Instrum. 2006;77(10):103905. doi: 10.1063/1.2360884

[167] Speziale S, Marquardt H, Duffy TS. Brillouin scattering and its application in geosciences. Rev Mineral Geochem. 2014;78(1):543–603. doi: 10.2138/rmg.2014.78.14

[168] Bridoux E, Rouvaen JM, Moriamez M, et al. Optoacoustic testing of microsound devices. J Appl Phys. 1974;45(12):5156–5159. doi: 10.1063/1.1663209

[169] Gusev V, Karabutov A. Laser optoacoustics. NY: AIP; 1993.

[170] Lomonosov A, Mayer AP, Hess P. 3. Laser-based surface acoustic waves in materials science. In: Levy M, Bass HE Stern R, editors. Experimental methods in the physical sciences. Vol. 39. Cambridge, Massachusetts: Academic Press; 2001. p. 65–134.

[171] Pawbake A, Zerr A, Rosuel A, et al. A new superhard nitride superconductor. In: 25ème Congrès Général de la Société Française de Physique. Contribution ID: 130. Nantes, France: Société Française de Physique. 2019 July 8–12.

[172] Torquato S. Random heterogeneous materials. NY: Springer; 2002.

[173] Kuriakose M, Raetz S, Hu QM, et al. Longitudinal sound velocities, elastic anisotropy, and phase transition of high-pressure cubic H_2_O ice to 82 GPa. Phys Rev B. 2017;96(13):134122. doi: 10.1103/PhysRevB.96.134122

[174] Raetz S, Kuriakose M, Djemia P, et al. Elastic anisotropy and single-crystal moduli of solid argon up to 64 GPa from time-domain Brillouin scattering. Phys Rev B. 2019;99(22):224102. doi: 10.1103/PhysRevB.99.224102

[175] Kuriakose M, Chigarev N, Raetz S, et al. *In situ* imaging of the dynamics of photo-induced structural phase transition at high pressures by picosecond acoustic interferometry. New J Phys. 2017;19(5):053026. doi: 10.1088/1367-2630/aa6b3d

[176] Kuriakose M, Raetz S, Chigarev N, et al. Picosecond laser ultrasonics for imaging of transparent polycrystalline materials compressed to megabar pressures. Ultrasonics. 2016;69:259–267. doi: 10.1016/j.ultras.2016.03.007 27026585

[177] Sathyan S, Threard T, De Lima Savi E, et al. Time-domain Brillouin scattering by quasi-longitudinal and quasi-shear acoustic pulses for 3D characterization of individual ice grains at high pressure. to be submitted. 2020.

[178] Hoermann K, Vilser A. Einsatz neuer Werkzeugwerkstoffe - wichtiges Element zur Leistungssteigerung der technologischen Prozesse. Technik. 1979;34:560–561.

[179] Wilks J, Wilks E. Properties and applications of diamond. 1st. ed. Oxford: Butterworth-Heinemann; 1991.

[180] Schuh F, editor. Enzyklopädie Naturwissenschaft und Technik. München: Verlag Moderne Industrie; 1979.

[181] Bewilogua K, Richter F. Vapor phase deposition of cubic boron nitride films. In: Riedel R, editor. Handbook of ceramic hard materials. Vol. 1. Weinheim: Wiley-VCH; 2000. p. 420–445.

[182] Field JE. Appendix: tables of properties. In: Field JE, editor. The properties of natural and synthetic diamond. San Diego: Academic Press; 1992. p. 667–699.

[183] Taniguchi T, Akaishi M, Yamaoka S. Machanical properties of polycrystalline translucent cubic boton nitride as characterized by the Vickers indentation method. J Am Ceram Soc. 1996;79(2):547–549. doi: 10.1111/j.1151-2916.1996.tb08165.x

[184] Sumiya H, Uesaka S, Satoh S. Mechanical properties of high purity polycrystalline cBN synthesized by direct conversion sintering method. J Mater Sci. 2000;35(5):1–6. doi: 10.1023/A:1004780218732

[185] Röhrig K. Gefüge und Beständigkeit gegen Mineralverschleiß von carbidischem Gußeisen. Giesserei. 1971;58:697–705.

[186] Thévenot F. Bororn carbide - a comprehensive review. J Europ Ceram Soc. 1990;6(4):205–225. doi: 10.1016/0955-2219(90)90048-K

[187] Rice RW, Wu CC, Borchelt F. Hardness-grain-size relations in ceramics. J Am Ceram Soc. 1994;77(10):2539–2553. doi: 10.1111/j.1151-2916.1994.tb04641.x

[188] Komeya K, Matsui M. High temperature engeneering ceramics. In: Swain MV, editor. Structure and properties of ceramics. Materials science and technology. Vol. 11. Weinheim: VCH; 1994. p. 517–565.

[189] Bhaumik SK, Divakar C, Usha Devi S, et al. Synthesis and sintering of SiC under high pressure and high temperature. J Mater Res. 1999;14(3):906–911. doi: 10.1557/JMR.1999.0121

[190] Lee HC, Gurland J. Hardness and deformation of cemented tungstn carbide. Mater Sci Eng. 1978;33(1):125–133. doi: 10.1016/0025-5416(78)90163-5

[191] Telle R. Borides. In: Bloor D, Brook RJ Flemings MC, et al., editors. The encyclopedia of advanced materials. Vol. 1. Oxford: Elsevier Science Ltd.; 1994. p. 287–292.

[192] Niihara K, Hirai T. Chemical vapour-deposited silicon nitride. Part 4 hardness characterisation. J Mater Sci. 1977;12(6):1243–1252. doi: 10.1007/BF02426863

[193] Ziegler G, Heinrich J, Wötting G. Review: relationships between processing, microstructure and properties of dense and reaction-bonded silicon nitride. J Mater Sci. 1987;22(9):3041–3086. doi: 10.1007/BF01161167

[194] Dusza J, Eschner T, Rundgren K. Hardness anisotropy in bimodal grained gas pressure sintred Si_3_N_4_. J Mater Sci Lett. 1997;16(20):1664–1667. doi: 10.1023/A:1018521930114

[195] Tsukuma K, Shimada M, Koizumi M. Thermal conductivity and microhardness of Si_3_N_4_ with and without additives. Am Ceram Soc Bull. 1981;60:910–912.

[196] Petzow G, Herrmann M. Silicon nitride ceramics. In: Jansen M, editor. High performance non-oxide ceramics II. Structure and bonding. Berlin, Heidelberg: Springer; 2002. p. 47–167.

[197] Lengauer W. Properties of bulk d-TiN1-x prpared by nitrogen diffusion into titanium metal. J Alloys Comp. 1992;186(2):293–307. doi: 10.1016/0925-8388(92)90016-3

[198] Chen X-J, Struzhkin VV, Wu Z, et al. Hard superconducting nitrides. Proc Natl Acad Sci U S A. 2005;102(9):3198–3201. doi: 10.1073/pnas.0500174102 PMC552926 15728352

[199] Zakutayev A, Perkins CL. Influence of protection layers on thermal stability of nitride thin films. Phys Status Solidi RRL. 2021;15(8):2100178. doi: 10.1002/pssr.202100178

[200] Krell A, Bakun OV. High-temperature hardness of Al2O3-base ceramics. Acta Metall. 1986;34(7):1315–1319. doi: 10.1016/0001-6160(86)90018-0

[201] Bai J, Yang X, Xu S, et al. Fabrication of highly dense Al_2_O_3_ ceramics. Scr Materialia. 2013;68(6):393–395. doi: 10.1016/j.scriptamat.2012.10.054

[202] Patnaik P. Handbook of inorganic chemicals. Vol. 529. NY Chicago: McGraw-Hill; 2003.

[203] Ma SL, Zhao YS, Tang RL, et al. Transparent β-Si_3_N_4_ and γ-Si_3_N_4_ compacts synthesized with mixed-size precursor under high pressure and high temperature. Appl Phys Lett. 2021;119(17):171904. doi: 10.1063/5.0070380

[204] Zerr A. Elastic moduli of γ-Sn3N4. 2026.

[205] Chhowalla M, Unalan HE. Thin films of hard cubic Zr_3_N_4_ stabilized by stress. Nat Mater. 2005;4(4):317–322. doi: 10.1038/nmat1338 15765108

[206] Dzivenko DA, Zerr A, Mezouar M, et al. Multi-anvil high-pressure synthesis of cubic hafnium(IV) nitride, c-Hf_3_N_4_. In: (unpublished) presentation at 46th EHPRG International conference. Valencia; 2008 [2008 Sep 7–12].

[207] Gu ZQ, Hu CQ, Huang HH, et al. Identification and thermodynamic mechanism of the phase transition in hafnium nitride films. Acta Mater. 2015;90:59–68. doi: 10.1016/j.actamat.2015.02.026

[208] Bourguille J, Brinza O, Zerr A. Vickers microhardness and indentation fracture toughness of tantalum sesquinitride, η-Ta_2_N_3_. Ceram Int. 2016;42(1):982–985. doi: 10.1016/j.ceramint.2015.08.130

[209] Šajgalik P, Dusza J, Hoffmann MJ. Relationship between microstructure, toughening mechanisms, and fracture toughness of reinforced silicon nitride ceramics. J Am Ceram Soc. 1995;78(10):2619–2624. doi: 10.1111/j.1151-2916.1995.tb08031.x

[210] Feldbach E, Museur L, Krasnenko V, et al. Defects induced by He^+^ irradiation in γ-Si_3_N_4_. J Lumines. 2021;237:118132. doi: 10.1016/j.jlumin.2021.118132

[211] Sickafus KE, Minervini L, Grimes RW, et al. Radiation tolerance of complex oxides. Science. 2000;289(5480):748–751. doi: 10.1126/science.289.5480.748 10926530

[212] Sickafus KE, Grimes RW, Valdez JA, et al. Radiation-induced amorphization resistance and radiation tolerance in structurally related oxides. Nat Mater. 2007;6(3):217–223. doi: 10.1038/nmat1842 17322869

[213] Gilbert CA, Smith R, Kenny SD, et al. A theoretical study of intrinsic point defects and defect clusters in magnesium aluminate spinel. J Phys-Condes Matter. 2009;21(27):275406. doi: 10.1088/0953-8984/21/27/275406 21828490

[214] Navrotsky A. Repeating patterns in mineral energetics. Am Min. 1994;79(7–8):589–605.

[215] Pellerin N, Dodane-Thiriet C, Montouillout V, et al. Cation sublattice disorder induced by swift heavy ions in MgAL_2_O_4_ and ZnAl_2_O_4_ spinels: Al-27 solid-state NMR study. J Phys Chem B. 2007;111(44):12707–12714. 10.1021/jp072620t 17935313

[216] Moreno M, Andersson JM, M’Saoubi R, et al. Adhesive wear of TiAlN coatings during low speed turning of stainless steel 316L. Wear. 2023;524:204838. doi: 10.1016/j.wear.2023.204838

[217] Tatarko P, Kasiarova M, Dusza J, et al. Effect of various grain boundary phases and SiC addition on the fracture characteristics of Si_3_N_4_ based ceramics. Chem Listy. 2011;105(S5):S850–S851.

[218] Balog M, Keckes J, Schoberl T, et al. Nano/macro-hardness and fracture resistance of Si_3_N_4_/SiC composites with up to 13 wt.% of SiC nano-particles. J Eur Ceram Soc. 2007;27(5):2145–2152. doi: 10.1016/j.jeurceramsoc.2006.08.010

[219] Kovalcikova A, Dusza J, Sajgalik P. Thermal shock resistance and fracture toughness of liquid-phase-sintered SiC-based ceramics. J Eur Ceram Soc. 2009;29(11):2387–2394. doi: 10.1016/j.jeurceramsoc.2009.01.021

[220] Mo S-D, Ouyang L, Ching WY, et al. Interesting physical properties of the new spinel phase of Si_3_N_4_ and C_3_N_4_. Phys Rev Lett. 1999;83(24):5046–5049. doi: 10.1103/PhysRevLett.83.5046

[221] Bagayoko D, Zhao GL. Predicted electronic properties of cubic Si_3_N_4_. Physica C. 2001;364–365:261–264. doi: 10.1016/S0921-4534(01)00768-7

[222] Ching WY, Mo S-D, Tanaka I, et al. Prediction of spinel structure and properties of single and double nitrides. Phys Rev B. 2001;63(6):064102. doi: 10.1103/PhysRevB.63.064102

[223] Ching WY, Mo S-D, Ouyang L. Electronic and optical properties of the cubic spinel phase of Si_3_N_4_, Ge_3_N_4_, SiGe_2_N_4_, and GeSi_2_N_4_. Phys Rev B. 2001;63(24):245110. doi: 10.1103/PhysRevB.63.245110

[224] Ching WY, Rulis P. *Ab initio* calculation of the electronic structure and spectroscopic properties of spinel γ-Sn_3_N_4_. Phys Rev B. 2006;73(4):045202. doi: 10.1103/PhysRevB.73.045202

[225] Chu IH, Kozhevnikov A, Schulthess TC, et al. All-electron GW quasiparticle band structures of group 14 nitride compounds. J Chem Phys. 2014;141(4):044709. doi: 10.1063/1.4890325 25084939

[226] Hu H, Peslherbe GH. Accurate mechanical and electronic properties of spinel nitrides from density functional theory. J Phys Chem C. 2021;125(17):8927–8937. doi: 10.1021/acs.jpcc.0c09896

[227] Oba F, Tatsumi K, Adachi S, et al. *N*- and *p*-type dopants for cubic silicon nitride. Appl Phys Lett. 2001;78(11):1577–1579. doi: 10.1063/1.1354667

[228] Oba F, Tatsumi K, Tanaka I, et al. Effective doping in cubic Si_3_N_4_ and Ge_3_N_4_: a first-principles study. J Am Ceram Soc. 2002;85(1):97–100. doi: 10.1111/j.1151-2916.2002.tb00046.x

[229] Dong JJ, Deslippe J, Sankey OF, et al. Theoretical study of the ternary spinel nitride system Si_3_N_4_-Ge_3_N_4_. Phys Rev B. 2003;67(9):094104. doi: 10.1103/PhysRevB.67.094104

[230] Zerr A, Schwarz M, Schmechel R, et al. New high-pressure nitrides. Acta Cryst A. 2002;58(suppl.):C47.

[231] Nishiyama N. Fabrication of transparent polycrystalline cubic silicon nitride: physical properties and phase boundary. Rev High Press Sci Technol. 2020;30(3):210–215.

[232] Schmechel R. Personal communication. 2002.

[233] Museur L, Zerr A, Kanaev A. Photoluminescence and electronic transitions in cubic silicon nitride. Sci Rep. 2016;6(1):18523. doi: 10.1038/srep18523 PMC4698759 26725937

[234] Leitch S, Moewes A, Ouyang L, et al. Properties of non-equivalent sites and bandgap of spinel-phase silicon nitride. J Phys Condens Matter. 2004;16(36):6469–6476. doi: 10.1088/0953-8984/16/36/012

[235] Boyko TD, Bailey E, Moewes A, et al. Class of tunable wide band gap semiconductors γ-(Ge_x_Si_1-x_)_3_N_4_. Phys Rev B. 2010;81(15):155207.

[236] Boyko TD, Hunt A, Zerr A, et al. Electronic structure of spinel-type nitride compounds Si_3_N_4_, Ge_3_N_4_, and Sn_3_N_4_ with tuneable band gaps: application to light emitting diodes. Phys Rev Lett. 2013;111(9):097402. 10.1103/PhysRevLett.111.097402 24033070

[237] Blakemore JS. Semiconducting and other major properties of gallium arsenide. J Appl Phys. 1982;53(10):R123–R181. doi: 10.1063/1.331665

[238] Nam SB, Reynolds DC, Litton CW, et al. Free-exciton energy spectrum in GaAs. Phys Rev B. 1976;13(2):761–767. doi: 10.1103/PhysRevB.13.761

[239] Viswanath AK, Lee JI, Kim D, et al. Exciton-phonon interactions, exciton binding energy, and their importance in the realization of room-temperature semiconductor lasers based on GaN. Phys Rev B. 1998;58(24):16333–16339. doi: 10.1103/PhysRevB.58.16333

[240] Clark CD, Dean PJ, Harris PV. Intrinsic edge absorption in diamond. Proc R Soc Lond A-Math Phys Sci. 1964;277(1370):312–329. doi: 10.1098/rspa.1964.0025

[241] Kroll P. Hafnium nitride with thorium phosphide structure: physical properties and an assessment of the Hf-N, Zr-N, and Ti-N phase diagrams at high pressures and temperatures. Phys Rev Lett. 2003;90(12):125501. doi: 10.1103/PhysRevLett.90.125501 12688881

[242] Xu M, Wang S, Yin G, et al. Optical properties of cubic Ti_3_N_4_, Zr_3_N_4_, and Hf_3_N_4_. Appl Phys Lett. 2006;89(15):151908. doi: 10.1063/1.2360937

[243] Chihi T, Fatmi M, Ghebouli B, et al. Theoretical prediction of the structural, elastic, electronic and optical properties of Zr(3)N(4) and Hf(3)N(4) compounds. Solid State Sci. 2011;13(7):1414–1419. doi: 10.1016/j.solidstatesciences.2011.04.014

[244] Feng L-P, Wang Z-Q, Liu Z-T. First-principles calculations on electronic, chemical bonding and optical properties of cubic Hf_3_N_4_. Commun Theor Phys. 2013;59(1):105–109. doi: 10.1088/0253-6102/59/1/19

[245] Chen Y, Cheng W, Liao B, et al. Structural, elastic and electronic properties of oxygen substitution in cubic Zr_3_N_4_ and Ti_3_N_4_. Int J Mod Phys B. 2013;27(18):1350095. doi: 10.1142/S0217979213500951

[246] Zheng F, Fang Y, Yu S, et al. Exploration of crystal structures and phase transitions in Hf_3_N_4_. Cryst Eng Comm. 2017;19(19):2608–2613. doi: 10.1039/C7CE00524E

[247] Yablonskikh M, Dzivenko D, Bourguille J, et al. Electronic structure and band gap of oxygen bearing c-Zr_3_N_4_ and of c-Hf_3_N_4_ by soft X-ray spectroscopy. Phys Status Solidi A. 2014;211(4):835–842. doi: 10.1002/pssa.201330338

[248] Boyko TD, Zerr A, Moewes A. Tuning the electronic band gap of oxygen-bearing cubic zirconium nitride: cZr_3−x_(N_1−x_O_x_)_4_. ACS Appl Electron Mater. 2021;3(11):4768–4773. doi: 10.1021/acsaelm.1c00632

[249] Liu YH, Wang KL, Zhang XS, et al. Experimental and theoretical study of the electronic structure and optical and mechanical properties of Th_3_P_4_-Type Hf_3_N_4_ thin films for applications in infrared windows. ACS Appl Nano Mater. 2025;8(13):6671–6678.

[250] Richter A, Hermle M, Glunz SW. Reassessment of the limiting efficiency for crystalline silicon solar cells. IEEE J Photovolt. 2013;3(4):1184–1191. doi: 10.1109/JPHOTOV.2013.2270351

[251] Müller A, Friedrich L, Reichel C, et al. A comparative life cycle assessment of silicon PV modules: impact of module design, manufacturing location and inventory. Sol Energy Mater Sol Cells. 2021;230:111277. doi: 10.1016/j.solmat.2021.111277

[252] Al-Ashouri A, Kohnen E, Li B, et al. Monolithic perovskite/silicon tandem solar cell with >29% efficiency by enhanced hole extraction. Science. 2020;370(6522):1300–1309. doi: 10.1126/science.abd4016 33303611

[253] Ewing SM. Tin. Mineral commodity summaries 2026. Reston (VA): U.S. Department of the Interior, U. S. Geological Survey; 2026. p. 194–195.

[254] Marschall R. 50 years of materials research for photocatalytic water splitting. Eur J Inorg Chem. 2021;2021(25):2435–2441. doi: 10.1002/ejic.202100264

[255] Ng KH, Lai SY, Cheng CK, et al. Photocatalytic water splitting for solving energy crisis: myth, fact or busted? Chem Eng J. 2021;417:128847. doi: 10.1016/j.cej.2021.128847

[256] Khaselev O, Turner JA. A monolithic photovoltaic-photoelectrochemical device for hydrogen production via water splitting. Science. 1998;280(5362):425–427. doi: 10.1126/science.280.5362.425 9545218

[257] Caskey CM, Seabold JA, Stevanovic V, et al. Semiconducting properties of spinel tin nitride and other IV_3_N_4_ polymorphs. J Mater Chem C. 2015;3(6):1389–1396. doi: 10.1039/C4TC02528H

[258] Sanchez-Palencia P, Garcia G, Conesa JC, et al. Spinel-type nitride compounds with improved features as solar cell absorbers. Acta Mater. 2020;197:316–329. doi: 10.1016/j.actamat.2020.07.034

[259] Bikowski A, Siol S, Gu J, et al. Design of metastable tin titanium nitride semiconductor alloys. Chem Mat. 2017;29(15):6511–6517. doi: 10.1021/acs.chemmater.7b02122

[260] Zerr A, Boehler R. Constraints on the melting temperature of the lower mantle from high-pressure experiments on MgO and magnesiowustite. Nature. 1994;371(6497):506–508. doi: 10.1038/371506a0

[261] Zerr A, Serghiou G, Boehler R. Melting of CaSiO_3_ perovskite to 430 kbar and first *in-situ* measurements of lower mantle eutectic temperatures. Geophys Res Lett. 1997;24(8):909–912. doi: 10.1029/97GL00829

[262] Bridgman PW. Recent work in the field of high pressures. Rev Mod Phys. 1946;18(1):1–93. doi: 10.1103/RevModPhys.18.1

[263] Bradley RS, Munro DC. High pressure chemistry. Oxford (NY): Pergamon Press; 1965. (Robinson R, editor. The Commonwealth and international library of science, technology, engineering, and liberal studies. Chemistry division).

[264] Range K-J. Festkörperchemie bei hohen Drücken. Chem unserer Zeit. 1976;10(6):180–188. doi: 10.1002/ciuz.19760100604

[265] Yoo CS, Akella J, Cynn H. Direct elementary reactions of boron and nitrogen at high pressures and temperatures. Phys Rev B. 1997;56(1):140–146. doi: 10.1103/PhysRevB.56.140

[266] Yoo C-S, Akella J, Nicol M. Chemistry at high pressures and temperatures: in-situ synthesis and characterization of β-Si_3_N_4_ by DAC X-ray/laser heating studies. In: Akaishi M, editor. The 3rd NIRIM International Symposium on Advanced Materials (ISAM’96). Tsukuba, Japan. NIRIM; 1996 Mar 4–8; p. 175–179.

[267] Nesting DC, Badding JV. High-pressure synthesis of sp2-bonded carbon nitrides. Chem Mater. 1996;8(7):1535–1539. doi: 10.1021/cm9601289

[268] Serghiou G, Zerr A, Boehler R. (MgFe)SiO_3_ -perovskite stability under lower mantle conditions. Science. 1998;280(5372):2093–2095. doi: 10.1126/science.280.5372.2093 9641909

